# Unravelling the γ-butyrolactone network in *Streptomyces coelicolor* by computational ensemble modelling

**DOI:** 10.1371/journal.pcbi.1008039

**Published:** 2020-07-10

**Authors:** Areti Tsigkinopoulou, Eriko Takano, Rainer Breitling

**Affiliations:** 1 DTU Biosustain, Novo Nordisk Foundation Center for Biosustainability, Technical University of Denmark, Kgs. Lyngby, Denmark; 2 Manchester Institute of Biotechnology, School of Natural Sciences, University of Manchester, Manchester, United Kingdom; King’s College London, UNITED KINGDOM

## Abstract

Antibiotic production is coordinated in the *Streptomyces coelicolor* population through the use of diffusible signaling molecules of the γ-butyrolactone (GBL) family. The GBL regulatory system involves a small, and not completely defined two-gene network which governs a potentially bi-stable switch between the “on” and “off” states of antibiotic production. The use of this circuit as a tool for synthetic biology has been hampered by a lack of mechanistic understanding of its functionality. We here present the creation and analysis of a versatile and adaptable ensemble model of the *Streptomyces* GBL system (detailed information on all model mechanisms and parameters is documented in http://www.systemsbiology.ls.manchester.ac.uk/wiki/index.php/Main_Page). We use the model to explore a range of previously proposed mechanistic hypotheses, including transcriptional interference, antisense RNA interactions between the mRNAs of the two genes, and various alternative regulatory activities. Our results suggest that transcriptional interference alone is not sufficient to explain the system’s behavior. Instead, antisense RNA interactions seem to be the system's driving force, combined with an aggressive *scbR* promoter. The computational model can be used to further challenge and refine our understanding of the system’s activity and guide future experimentation.

## Author summary

*Streptomyces* species are Gram-positive soil-dwelling bacteria, which are known as a prolific source of secondary metabolites, such as antibiotics. Antibiotic production is coordinated in the bacterial population through the use of diffusible signalling molecules of the γ-butyrolactone (GBL) family. The GBL regulatory system involves a small, yet complex two-gene network, the mechanism of which has not yet been completely defined. The complete elucidation of this system could potentially lead to the ability to design reliable and sensitive engineered cellular switches. We therefore designed a versatile model of the GBL system in order to investigate the feasibility of various hypothesized mechanisms. The ensemble modelling analysis that we performed revealed that antisense RNA interactions seem to be the system’s driving force, together with an aggressive *scbR* promoter. Transcriptional interference is also significant; however, it is not sufficient to explain the system’s behavior by itself. Finally, the model indicates key experiments, which could completely elucidate the role of the system and the interactions of its components and potentially lead to the design of reliable and sensitive systems with significant applications as orthologous regulatory circuits in synthetic biology and biotechnology.

## Introduction

The core aim of synthetic biology is the design and engineering of complex biological systems with functionalities that do not exist in nature. In order to accomplish this, reliable regulatory circuits are required, which enable the precise control of gene expression over a wide range of conditions.[1] The “quorum sensing” (QS) system of the bacterium *Vibrio fischeri* is a prominent example of such a circuit.[2] However, although the quorum sensing circuit has been widely employed in synthetic biology with numerous successful applications,[3–5] QS-derived regulatory systems have some important limitations, such as potential crosstalk between different circuits due to the promiscuity of the signaling molecules or the promoters,[6] and the problematic implementation in eukaryotic organisms.[7] Novel orthogonal circuits would therefore be very welcome.

A good candidate for this purpose could be the γ-butyrolactone (GBL) signaling circuits of *Streptomyces coelicolor*[1], which have been used in proof-of-concept studies in mammalian and bacterial systems [8, 9]. Streptomycetes are Gram-positive, filamentous, soil bacteria, which produce antibiotics to eliminate their competitors in unfavorable environmental conditions. As the antibiotic compounds can be toxic even to the producing strains, their biosynthesis needs to be carefully regulated in a population. This is achieved via the SCB1γ-butyrolactones, a group of signaling molecules associated with the regulation of antibiotic production and some aspects of bacterial morphology. The structure of the circuit also has similarities to the quorum sensing system, as it involves two genes and their respective proteins (ScbR and ScbA). ScbR belongs to the TetR family of repressors and inhibits its own transcription, as well as the transcription of the divergently encoded ScbA, which is the synthase of the butyrolactone signaling molecule SCB1. Furthermore, it represses *cpkO*, a regulatory gene for the CPK antibiotic biosynthesis gene cluster.[10, 11] SCB1 binds to ScbR, effectively deactivating the DNA binding activity and thus leading to the further production of butyrolactones. The CPK cluster is also activated, leading to the production of antibiotics.

Apart from this general scheme, little further mechanistic detail is known about the GBL circuits, although various hypotheses have been put forward. The two genes are transcribed in opposite directions and their promoters overlap by 53 base pairs. In previous studies it has been generally reported that sometimes divergent overlapping promoters are responsible for regulating the expression of genes.[12–14] This topology has been suggested to also be important in the GBL circuit, determining the precise switch of the system at relatively low concentrations.[1, 15] Another scenario that has been suggested is the formation of a putative complex between ScbA and ScbR proteins which acts in a similar manner as the LuxR–AHL complex in quorum sensing and further enhances the transcription of the *scbA* gene.[16] Alternatively, ScbR protein alone has also been hypothesized to have both repressor and activator functions (repressing itself and activating *scbA*).[17] Finally, studies in different bacteria and eukaryotes have shown that, generally, small RNA (antisense RNA) interactions can play an important role in cellular processes (e.g. transcription, translation, gene regulation).[18, 19] This has also been suggested to occur in the GBL system in *S*. *coelicolor*, where RNA transcripts from genes with overlapping promoters might interfere with each other’s activity by binding to each other and thus induce a form of internal regulation.[15, 20]

Previous computational modelling work on the GBL system is limited to two published models which investigated some of these scenarios. Mehra *et al*.[16] proposed a model based on the scenario of the ScbA–ScbR complex formation and Chatterjee *et al*.[15] focused on the effects of the overlapping promoters and antisense interaction. Both previously published models of the GBL circuit were focusing mostly on testing different parameter values and detecting the optimal combination for bistable behaviour. This approach was useful as a proof-of-concept application attempting to reproduce the qualitative behavior of the system. However, there is still doubt about whether the outcomes of the models are realistic and biologically plausible representations of the behavior expected for this circuit topology, rather than successful outliers. On top of all that, Mehra *et al*. reported that under no parameter set did the behavior of *scbR* manage to accurate predict the main qualitative features of the experimental data. Undoubtedly, in both previous studies, the limited availability of quantitative, precise parameter information has been hampering the modelling effort. This system therefore provides a good opportunity for the application of ensemble modelling strategies [21–24] that are able to cope with this limitation. In ensemble modelling entire ranges of plausible parameter values are considered, and a consensus regarding the possible behaviour of the circuit is achieved; this can potentially allow us to discriminate between the various proposed mechanisms, using the available experimental data on circuit behavior.

An ensemble modelling approach will therefore allow us to attempt to more clearly define the elusive regulatory mechanism of the GBL system while at the same time establishing a comprehensive computational model of the system, with sufficient predictive power to guide synthetic biology engineering strategies and future experimental work. This approach combined with the use of the same nomenclature as employed in key previous publications and the meticulous documentation of our modelling methods, parameters, assumptions and background information in a Media Wiki resource enables us to revisit, update and compare existing models. In this way, a principled evaluation of the different mechanistic proposals can be achieved, and we can move forward by rejecting previous assumptions *a posteriori*, on the basis of new evidence; this has previously been very challenging and has long been a desideratum of the modelling community.

More detailed explanations on the theoretical background of the GBL system and on the previous modelling work, can be found in the relevant MediaWiki page: http://www.systemsbiology.ls.manchester.ac.uk/wiki/index.php/Background_Information_on_GBL_system

## Methods and models

### Modelling of the *scbR/scbA* gene regulatory network

As the regulatory interactions in the GBL system have not yet been fully elucidated, our aim was to explore all the previously proposed mechanisms [15, 16] under the scope of realistic parameter values retrieved from the literature. In order to achieve this, we designed a unified meta-model that includes all the potential mechanisms and enables their individual or combined use by switching certain reactions “on” and “off”. Many aspects of the model are adapted from the quorum sensing model by Weber *et al*.[25]

**Fig 1:**
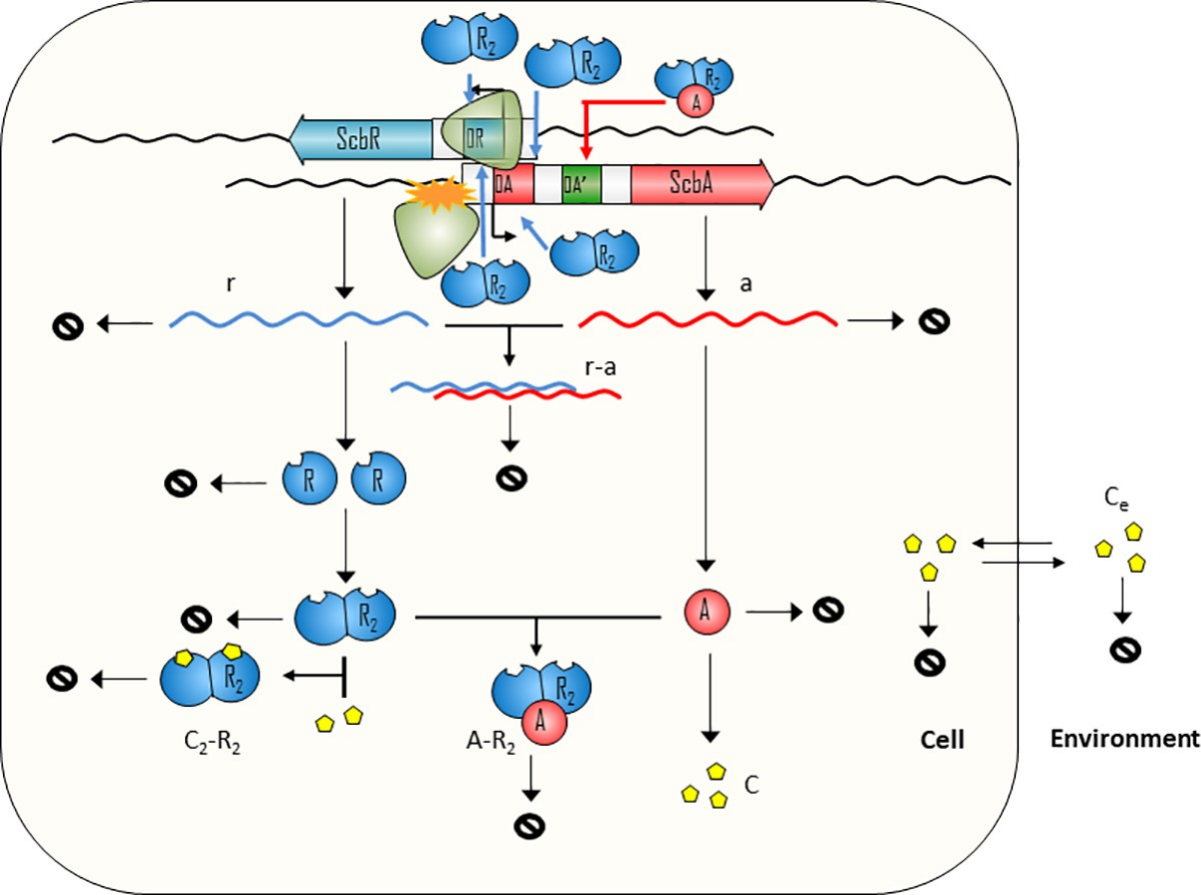
Schematic representation of the potential mechanisms of the ScbA/ScbR system. The *scbR* and *scbA* genes are divergently encoded and their promoter regions (O_R_ and O_A_ operators respectively) overlap by 53bp. Due to this promoter structure, RNA polymerase collisions prevent the transcription from both promoters at the same time. The mRNAs transcribed from *scbR* (*r*) and *scbA* (*a*) may also form a complex (*r•a*) which rapidly degrades, thus resulting in translational inhibition. The ScbR protein forms a homo-dimer (R_2_) which represses its own transcription as well as the transcription of *scbA*. The ScbA protein (A) is responsible for the production of the γ-butyrolactones (C) which bind to R_2_ and prevent it from binding to the O_R_ and O_A_ operators. Additionally, A may form a complex with R_2_ which simultaneously prevents R_2_ inhibition and activates the transcription of *a* by binding to a hypothetical O_A_’ operator. Alternatively, R_2_ may bind to its own operator and act as a repressor while at the same time activate the transcription of *a*. Finally, C diffuses freely from the cell to the external environment where it accumulates and can potentially diffuse into other neighbouring cells. In the modelled scenarios, some of the proposed regulatory mechanisms are selectively removed. The figure shown here corresponds to Scenario H; see ***Supplementary Fig Sp1 in S4 Appendix*** for the resulting circuits for scenarios A-G.

A schematic representation of the regulatory interactions considered in our model is shown in Fig 1. The ScbR homo-dimer binds to the operators of both *scbR* and *scbA* genes and represses their activity. As reported by Bhukya *et al*.,[26] two ScbR homo-dimers can bind to the operator. When one homo-dimer is bound, the mRNA transcription is already being repressed. As the concentration of ScbR rises, a second homo-dimer may bind to the already suppressed operator and further enhance the suppression of the transcription. ScbA protein (A), through an enzymatic reaction with glycerol derivatives and β-keto acid derivative precursors (S), produces the γ-butyrolactones (C). Our model considers the production of C to be proportional to the concentration of A. γ-butyrolactone (C) then creates a complex with the ScbR protein (C_2_•R_2_) and thus effectively deactivates it, enabling further production of ScbA. The signaling molecules (C) diffuse passively between the cells and the environment (C_e_) and thus accumulate in the culture medium. The model assumes that internal and external SCBs degrade at the same rate. Additionally, we assume that all molecules are homogeneously distributed both in the cytoplasm and in the medium. DNA duplication, degradation of chemical species and their dilution due to cellular growth are also considered.

The following alternative scenarios are investigated:

1. TI–The effect of transcriptional interference (collisions between the elongating RNAPs which leads to transcriptional termination) due to the overlap of the two genes’ promoter regions by 53 bp and by the convergent transcription of the two genes. This results in a decrease in expression of full-length mRNAs from both promoters and production of truncated mRNAs. *Note*: The transcriptional interference mechanism is considered to be present in all subsequent scenarios due to the gene topology in the native GBL system.
2. AS–The antisense effect conferred by convergent transcription of the *scbR* and *scbA* genes. In this case, transcripts with a segment of complementary sequence may lead to interactions between sense-antisense full length transcripts of the two genes, thus leading to the formation of a fast degrading complex of the two mRNAs and subsequent inhibition of translation.
3. RA–The formation of a complex between ScbA and ScbR proteins (ScbA–ScbR), which relieves ScbR repression, while at the same time activating the transcription of *scbA* and, in effect, the production of SCBs.
4. R_act_−The potential dual role of ScbR protein, which acts as a repressor for its own gene and as an activator for *scbA*[17)
5. Different combinations of the above scenarios (see **Table 1** and **Fig 1**), in order to evaluate both the effect of each isolated mechanism (except for Transcriptional Interference which was present in all scenarios) and their cumulative influence on the system’s behaviour.

**Table 1:** The simulated scenarios that include different combinations of the four investigated mechanisms.

	Mechanisms			
Scenarios	TI	RA	AS	R_act_
**A**	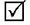			
**B**	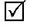	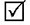		
**C**	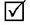		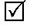	
**D**	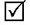			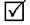
**E**	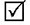	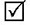	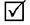	
**F**	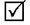	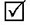		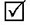
**G**	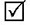		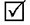	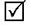
**H**	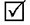	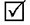	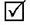	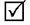

The full model comprises two compartments (cell and environment), 41 chemical reactions and 51 parameters. The initial concentrations for all species are zero; the only exceptions are the operators O_R_ and O_A_: for these one copy of each gene and, therefore, one copy of its corresponding promoter/operator are assumed for each cell. The list of the model species and the complete set of reactions for all scenarios are listed in **Supplementary Tables St1** and **St2 in S1 Appendix**. The corresponding differential equations for each species are shown in **Supplementary Table St3 in S1 Appendix**.

### State of promoters and transcription

In order to describe the overlapping promoter effects, the transcription reactions of each gene need to take into account the strength and the state of the gene’s promoter (free or occupied), as well as the potential interference from the transcription of the opposite gene. In order to accomplish this, a mathematical model for overlapping promoters proposed by Bendtsen *et al*.[27] was employed as described in **S1 Appendix** and in the Media Wiki page (http://www.systemsbiology.ls.manchester.ac.uk/wiki/index.php/Background_Information_on_GBL_system#Assumptions_in_the_improved_model)

*Note*: In the model, the activity of the two promoters is inferred by their corresponding parameters of promoter occupancy, promoter aspect ratio and promoter firing rates. In the ODEs, only the gene operators (O_R_ and O_A_) appear as species, in order to comply with the modelling nomenclature of the previous works and to reduce unnecessary complexity in the model.

### Cell growth and division

**Fig 2:**
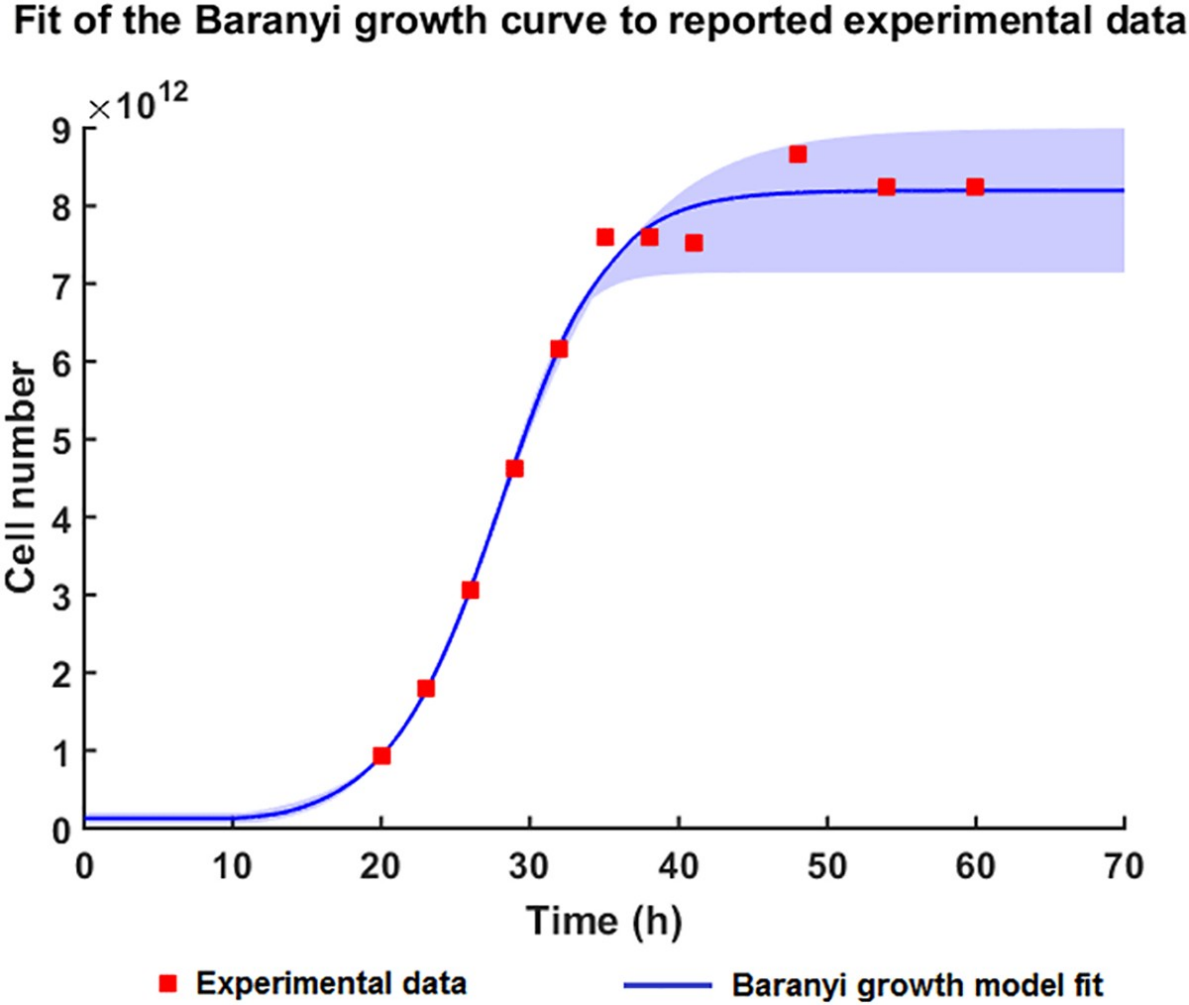
Fitting the Baranyi growth curve (Equation (9)-S1 Appendix) to the reported experimental data by Nieselt *et al*.[30] The carrying capacity (K) is the maximum plateau reached after 50h of growth. μ_max_ is the maximal growth rate achieved during the exponential phase of the growth. The range of parameter values used for fitting the experimental data to the equation are summarized in Table 2.

As the experimental time simulated is over 60 hours, the effects of cell growth must be taken into consideration in addition to the various regulatory mechanisms. In our model, the number of cells is described by a six-parameter Baranyi–Roberts model,[28, 29] (**Fig 2**) which takes into account the lag phase by using an adjustment function (**S1 Appendix**).

### Parameters

For each parameter of the model, a probability distribution was defined according to the available information from literature and experiments. In order to achieve this, a dedicated Media Wiki-based website was created (http://www.systemsbiology.ls.manchester.ac.uk/wiki/index.php/Welcome_to_the_In-Silico_Model_of_butyrolactone_regulation_in_Streptomyces_coelicolor) with the purpose of documenting parameter values along with explicit information on their sources and subsequent justification of conclusions about the most plausible values. By using this information, the log-normal probability distributions describing each parameter were inferred, according to a standardized protocol (https://doi.org/10.1038/s41596-018-0056-z) that systematically ranks the parameter information collected from all available sources (experiments, literature, databases etc.) and thus derives log-normal distributions that can be used as priors for sampling in an ensemble modelling framework.[22]

The parameter information of the probability distributions generated by this protocol is summarized in **Supplementary Table St4 in S1 Appendix**. The full information on the parameter values retrieved from the literature and the design of the corresponding probability distributions is included in the Wiki page.

The parameters for the cellular growth were derived from the experimental data for *Streptomyces* growth reported by Nieselt *et al*.[30] by performing a nonlinear least squares curve-fitting. By fitting the six-parameter Baranyi–Roberts equation (Equation (9)-**S1** Appendix) for bacterial growth to the number of cells (approximately calculated from the reported biomass values), the carrying capacity, the initial cell number and the maximum growth rate were estimated. As there were only 11 available experimental data points, a number of different parameter sets that all fitted the Baranyi–Roberts equation were identified, thus defining a confidence interval around the fitted data (**Fig 2**). The prediction of the carrying capacity by the logistic equation was 7.14∙10^12^–9∙10^12^ cells, which is very close to the 8.24∙10^12^ cells calculated from the final biomass. The parameter values for cellular growth are summarised in Table 2.

**Table 2:** Parameters for cell culture growth.

Parameter	Description	Range of values	Units
**K**	Carrying capacity	7.14∙10^12^ − 9∙10^12^	cells
**N_o_**	Initial number of cells	5∙10^10^ − 2∙10^11^	cells
**μ_max_**	Maximum growth rate	0.003368 − 0.007499	min^−1^
**v**	Curvature of the growth curve during the transition from lag to the exponential phase	0.001482 − 0.5814	min^−1^
**m**	Curvature of the growth curve during the transition from exponential to the stationary phase	0.47 − 2.46	n.a.
**λ**	Lag phase	330.52 − 883	min

### Ensemble modelling–Prior predictive check

Values for all parameters were sampled independently from the defined priors, and used for the simulation of the time course of all molecular species over 60 hours in each of the 8 scenarios. Possible correlations between parameters were not taken into account, as the relevant experimental information was not available. In the case of enzymatic reactions, where the kinetic parameter values are correlated as a result of thermodynamic constraints, the method introduced by Tsigkinopoulou et al.[22] could be used to account for the these correlations. A total of 10,000 parameter sets were examined in the ensemble, each representing a unique combination of plausible parameter values (the same parameter sets were used for all 8 scenarios). This means we were able to conduct a prior predictive check on the models to evaluate whether they can accommodate the available experimental data. This is a recommended and standard method for Bayesian model analysis, which recently has seen resurgent interest[31], although the underlying rationale was already eloquently presented by Box[32], who formally argued for the central importance of this approach as part of the model construction process. Model scenarios that added more complex molecular mechanisms, without improving the parametric robustness of the ensemble of models, could be rejected based on a parsimony argument. The more complex mechanisms might still be active, but do not appear to substantially influence the model behaviour within the range of plausible parameter combinations. The simulations were conducted by using the stiff ordinary differential equation solver ode15s in MATLAB R2016A. The Matlab files for all modelling scenarios are included in **S3 Appendix.**

## Results

The prior predictive check of the improved model was based on comparing the simulation results with transcriptomics data (**Supplementary Fig Sp2** in *S4* **Appendix**) reported in the publication by Nieselt *et al*.[30], as described in **Supplementary Table St5** in **S1 Appendix**. The behavioral features that formed the basis of the comparison were the profiles of the mRNA transcripts of *scbR* and *scbA* genes, and the activation threshold of the GBL system in terms of butyrolactone concentration. The choice of these features was based on the availability of experimental measurements of the system’s components and on the fundamental interest in the mRNA oscillatory behavior, which makes the circuit interesting as a target for applications in biotechnology. [8]

The focus of the analysis was not to find the “best fit” model, but to conduct a prior predictive check and assess the performance of all models under the full range of biologically feasible parameters. We use the parametric robustness of the models as an indicator to decide whether a potential mechanism is plausible (or influential) based on the number of models that seem to better accommodate the available experimental data. The parametric robustness here serves as an easily calculated proxy for the posterior probability of a particular model scenario. Each ensemble of models represents a specific hypothesis about the molecular mechanism of the biological system. An ensemble where many models (plausible parameter combinations) have a high total-log likelihood has a higher predictive density associated with this particular data set (in the sense of Box [32]), i.e. is a more plausible description of the biological system than alternative descriptions that are *a priori* equally credible, but result in a poorer overall fit or are more complex. In this way, we avoid the pitfall of overfitting and creating a model that primarily captures the features of experimental noise, but instead survey the entire lanscape of solutions and evaluate alternative options that may explain the experimental data equally well. The criterion for accommodating the data was set as a model having a total log-likelihood (TLL) >−140.

**Fig 3:**
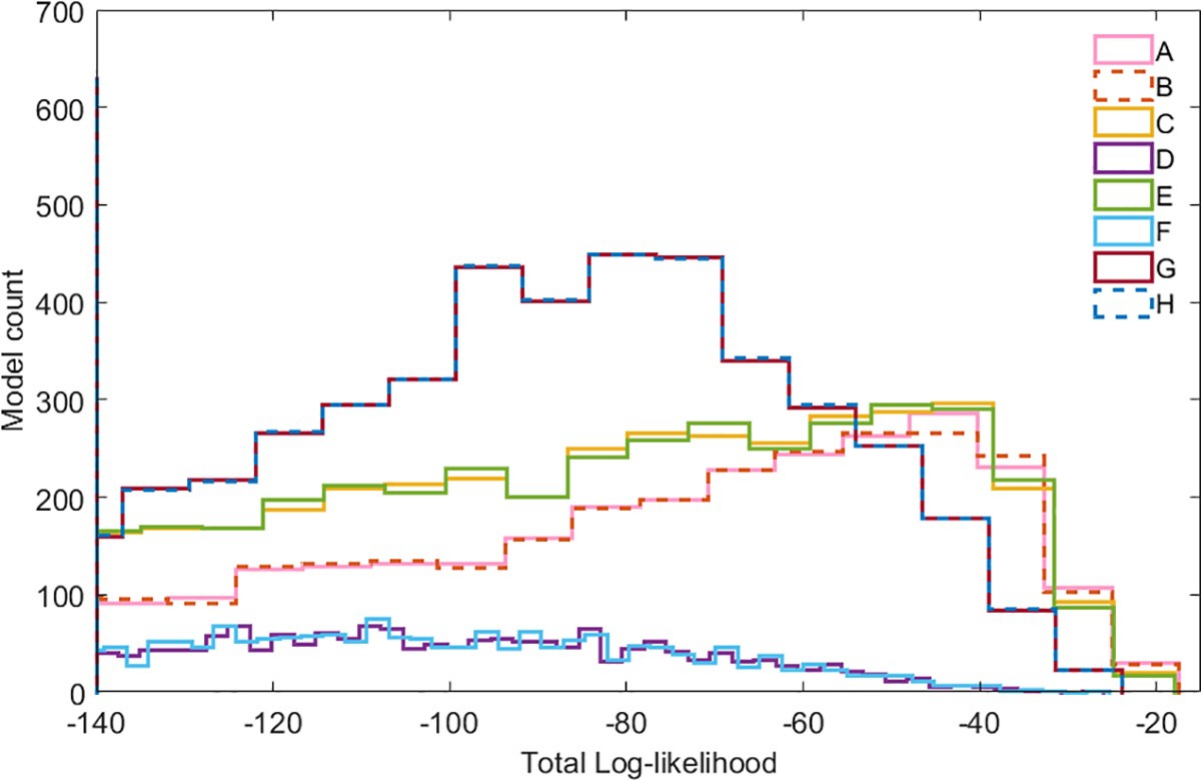
Total log-likelihood profiles for the 8 scenarios of the GBL model. Log-likelihood values closer to 0 (less negative) indicate a better overall fit to the experimental observations. Only the likelihood profiles of the highest ranked models, TLL >−140, are shown. Scenarios G and H (identical, overlapping curves) have the largest number of models with high TLL (>−140); however, it should be noted that scenarios A, B, C and E have a larger number of successful models in the highest ranks. Only scenarios D and F result in very low log likelihood profiles according to the experimental observations. The complete likelihood profiles are presented in **S4 Appendix** (Supplementary Figs Sp3(iii), Sp4(iii) and Sp5(iii)).

The total log-likelihood for the ensemble of 10,000 models in each of the 8 scenarios is shown in **Fig 3**. The complete likelihood profiles can be found in **Supplementary Figures Sp3-Sp5 in S4 Appendix**.

The log-likelihood analysis showed that the assumption of an AR complex did not improve any of the scenarios (B, E, F and H), as the number of models with high log likelihood scores essentially remained the same to the ones achieved by the rest of the mechanisms alone. Thus, this assumption can be rejected based on a parsimony argument. Similarly, ScbR being an activator achieved very low scoring results (scenarios D and F) unless it was combined with antisense RNA (scenarios G and H) where it showed a small but real improvement. However, even in those cases, the models do not manage to achieve high likelihood scores (models with TLL > −50) and the best results are lost. The best mechanisms seem to be A (transcriptional interference, TI) and C (TI combined with antisense RNA), with the latter resulting a slightly more robust ensemble of models, i.e. showing a consistently larger number of highly scoring models under different promoter strengths (i.e. different RNAP firing rates; **S2 Appendix**). These results are also supported by comparison of the average likelihood of each ensemble, i.e. its predictive density (**S2 Appendix**), based on the rationale presented by Box [32]. It therefore seems that among the three optional alternative mechanisms (AR complex, antisense RNA interaction, dual role of ScbR protein), only the presence of the antisense interaction had a consistent positive influence on parametric robustness, i.e. the quality of the fit of the model to the experimental observations across the range of plausible parameter values; its inclusion in the model is therefore most strongly supported by the experimental evidence.

**Fig 4:**
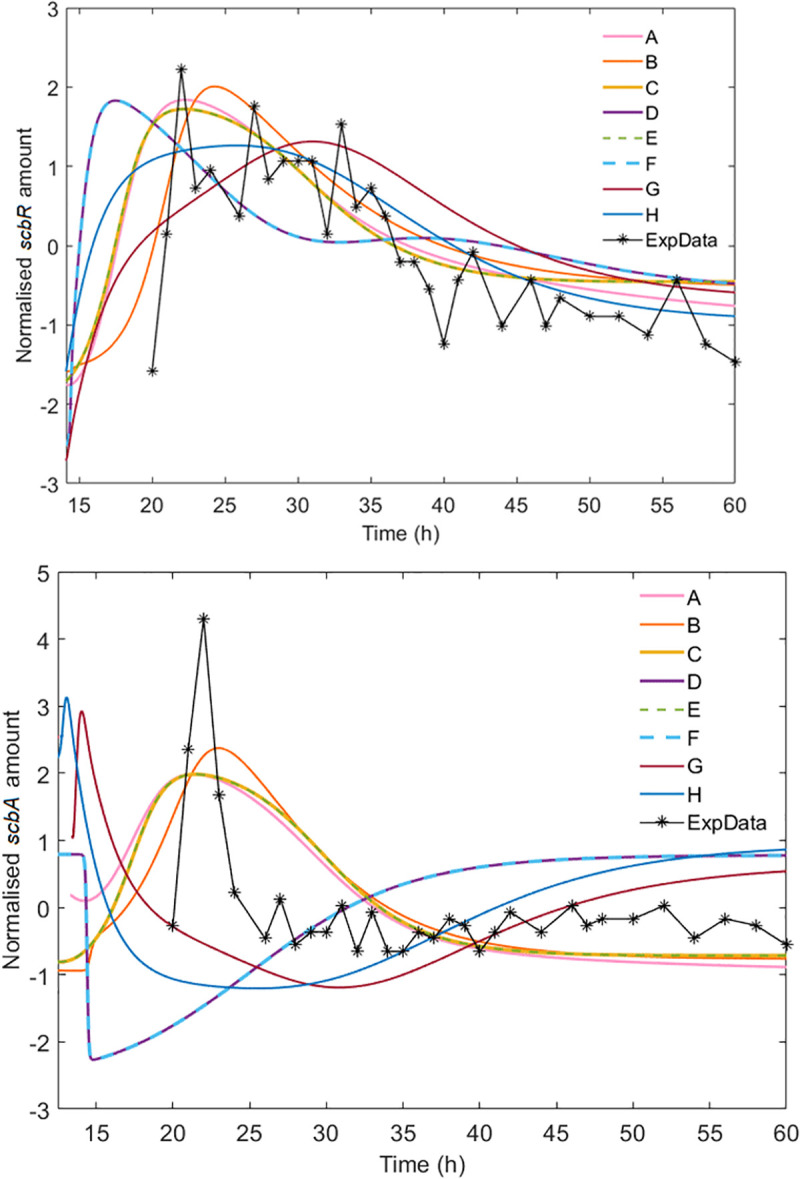
Comparison of the highest ranked models of the eight scenarios with the transcriptomics data. While all scenarios are able to explain *scbR* transcript dynamics reasonably well, only models A, B, C and E achieve a high log likelihood score for the *scbA* transcript. The scenarios C and E overlap completely, as do models D and F. None of the scenarios seems able to explain why the two peaks are so different in width.

The time course of the models in each ensemble that achieved the highest log-likelihood score against the experimental data (**Fig 4**) shows that the defined quality criteria were successful in capturing the features of interest in the two molecular species (*scbA* and *scbR* transcript). Furthermore, the models that best describe the experimental data seem to be scenarios A, B, C and E which do not include the ScbR activator mechanism (R_act_), although these very good matches (highest likelihood scores) are achieved only for a small part of the plausible parameter range. Furthermore, the fact that the curves for the scenarios C and E completely overlap, reinforces our belief that the AR complex and the transcriptional interference individually have a minimal effect on the model’s behavior once they are acting alongside the antisense RNA mechanism. Another interesting point is that none of the models were able to explain the difference in the width of the *scbR* and *scbA* peaks, although the R_act_ scenarios combined with antisense RNA (G and H) seem to better approximate the sharp decrease of *scbA*, albeit with incorrect timing. This might indicate that there is an additional regulatory effect that is not considered in our models (e.g., an additional undiscovered activator).

**Fig 5:**
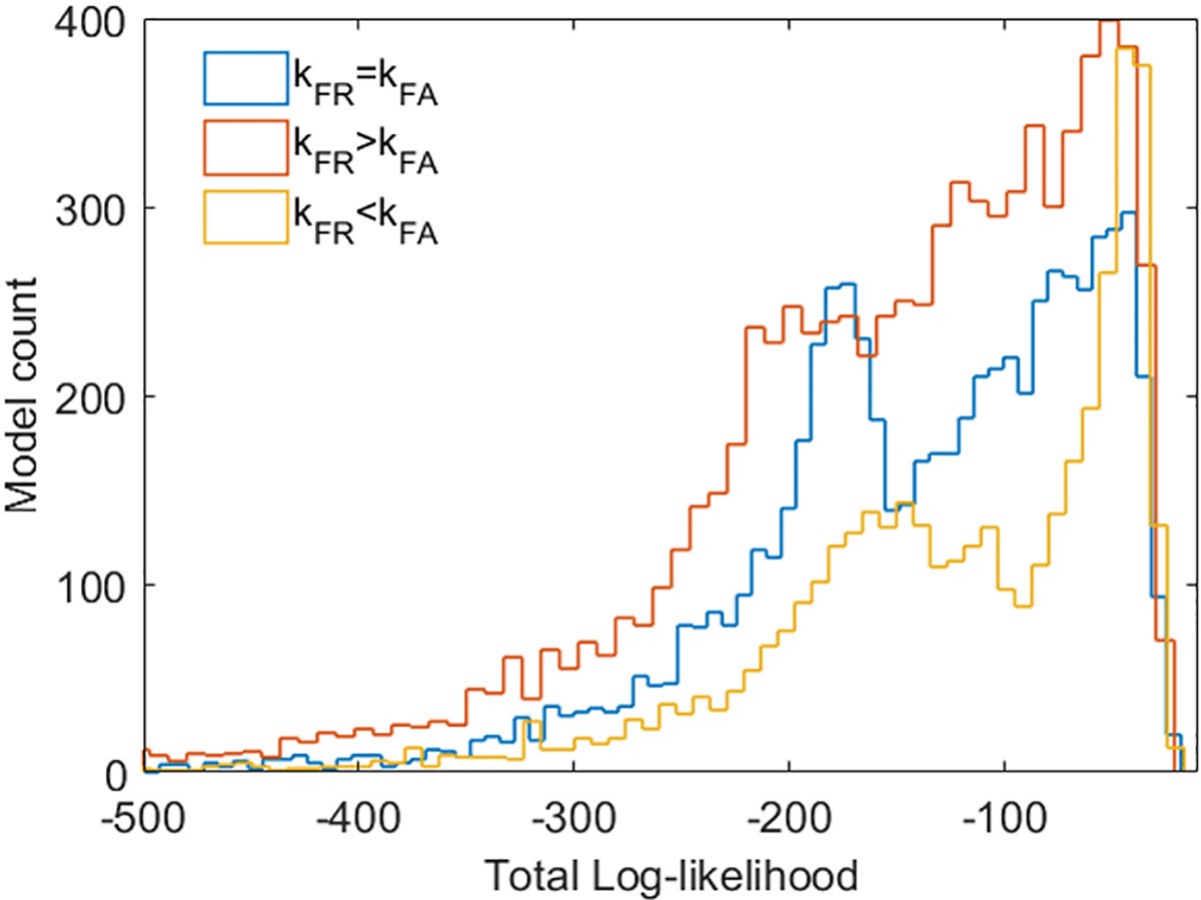
Comparison between the log-likelihood profiles of scenario C with varying promoter strengths. The amount of models that achieve highest rankings decrease when k_FR_ < k_FA_ and increase when k_FR_ > k_FA_. Furthermore, a stronger *scbR* promoter seems to significantly improve the behavior of *scbA*.

We also explored the effect of different promoter strengths on the quality of the model predictions. As can be seen in **Fig 5**, models with a stronger *scbR* promoter (k_FR_>k_FA_) had a substantially improved overall performance (increase in the number of highly ranked models; **S2 Appendix**).

**Fig 6:**
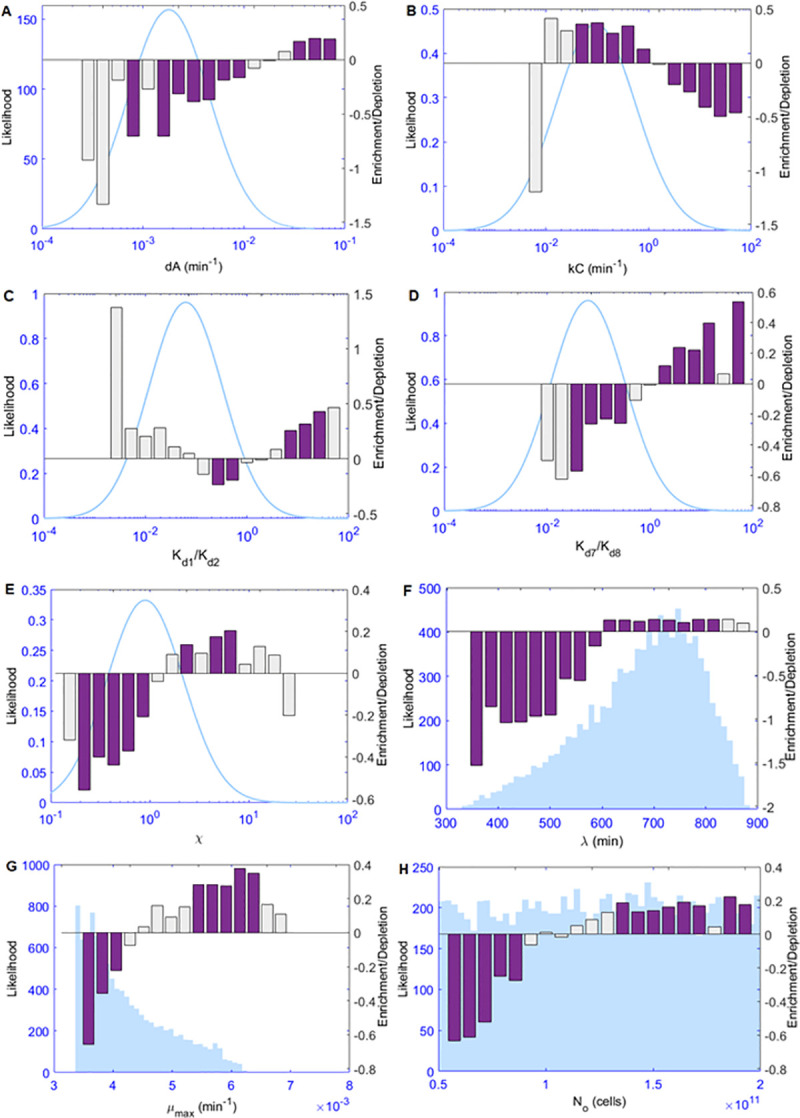
Comparison between the expected parameter values according to the defined priors and the actual parameters of models that had a TLL > −140. The bar plots represent the log-ratio of the actual parameter values over the expected ones in each bin, and the light blue lines in the background shows the original distribution of the sampled values. Purple bar plots correspond to statistically significant differences between the expected and the actual parameter values (two-tailed binomial test p-value < 0.05 corrected for multiple testing according to the number of bins), and the grey bar plots represent statistically insignificant deviations. The parameters with the most pronounced differences were the degradation rate of ScbA (6A), the synthesis rate of GBLs (6B), the ratio of K_d1_ (dissociation constant of binding of ScbR to O_R_) over K_d2_ (dissociation constant of binding of ScbR to O_A_) (6C), the ratio of K_d7_ (dissociation constant of binding of ScbR to ScbR-O_R_) over K_d8_ (dissociation constant of binding of ScbR to ScbR-O_A_) (6D) and the heterogeneity factor χ. With regards to the cellular growth, the most significant differences were observed in the lag phase λ (6F), the maximum growth rate μ_max_ (6G) and the initial number of cells N_o_ (6H).

In order to investigate which parameters are significantly affecting the model behavior in each scenario, we applied a Kolmogorov–Smirnov test[33] (K–S test) to those models from each scenario that had a TLL >−140, to see if the distribution of parameter values in the high-performing models differs significantly from the sampled values in the entire ensemble of models. The family-wise error rate was controlled by dividing the significance level by the number of tests performed (i.e., 44) to achieve a strict Bonferroni correction[34] for multiple testing. The complete K–S analysis results are included in **S2 Appendix**. The parameters that most prominently stood out in this analysis were the degradation rate of ScbA protein, the synthesis rate of SCB, the affinity parameters of the two promoters, and the heterogeneity factor describing the difference in promoter strength (most notably in the cases where *χ*<1). In order to more deeply investigate the regions of the parameter distributions which were more commonly encountered in the highly ranked models (TLL >−140), comparison plots between the defined priors and the actual parameters that belonged to the best models were designed (**Fig 6** and **Supplementary Figs Sp6-Sp29 in S4 Appendix**). In order to ensure that the observed enrichment or depletion of specific regions of the distributions was statistically significant, a two-tailed binomial test was performed to compare the theoretically expected and the actually observed parameter values in each bin. The p-values were corrected according to Benjamini and Hochberg to control the false discovery rate at 0.05.[35]. The resulting plots indicate the direction in which our prior beliefs about the parameter values should shift, i.e. they describe the updated beliefs that would be represented in the posterior distribution for each parameter. The parameter analysis revealed that a fast degradation of ScbA protein (**Fig 6A**) improved the behaviour of the models in all scenarios (particularly for dA> 0.01 min^-1^). The synthesis rate of GBLs (k_C_) also seemed to consistently be significant in all scenarios. In this case however, the extreme values were not preferred in the models with the highest log probability density, with the region of 0.01–1 min^-1^ being enriched (**Fig 6B**, **Supplementary Figs Sp6-Sp29 in S4 Appendix**). Furthermore, ratios of K_d1_ (dissociation constant of binding of ScbR to O_R_) over K_d2_ (dissociation constant of binding of ScbR to O_A_) which were larger than 1 were preferred over smaller ratios (**Fig 6C)**. The same applied for ratios of K_d7_ (dissociation constant of binding of ScbR to ScbR-O_R_) over K_d8_ (dissociation constant of binding of ScbR to ScbR-O_A_) (**Fig 6D)**. These findings suggest that O_A_ has a higher affinity than O_R_ for the ScbR protein. Finally, an investigation of the heterogeneity factor *χ*, revealed that in the simulations where the *scbA* promoter was stronger than the *scbR* promoter (k_FR_<K_FA_; *χ* < 1), the parameters of the optimal result models seem to cluster in the larger value region of *χ* (0.7 < *χ* < 0.9; **Supplementary Figs Sp11G, Sp14H and Sp20H in S4 Appendix**), meaning that the highest rankings in this group were achieved when the difference between the strengths of the two promoters was minimized. This, combined with the fact that in the simulations where *scbR* promoter was stronger than *scbA* (*χ* > 1), the heterogeneity factor was not influential any more, suggested that relative promoter strength is a defining factor for the model’s behaviour. Additional simulations on the scenarios where the value of *χ* was varied between 0.1 and 10 further supported this hypothesis, as the region between 1 and 10 was clearly enriched in the highly ranked models (most notably the values between 2 and 8; **Fig 6E**).

An investigation on the parameters of the growth curve showed that a longer lag phase was important for the quality of the models (**Fig 6F**), with the region between 10–14h being highly preferred in contrast to shorter lag phases (5–9 h). The maximum growth rate seemed to also affect the models’ performance on a secondary level, as faster growth rates (0.005–0.0065 min^-1^) seemed to be preferred (**Fig 6G**). As the growth parameters were not sampled from priors but were generated during the fitting of the experimental data to the growth equation, a two-sample Kolmogorov–Smirnov test (with an appropriate Bonferroni correction) was used to compare the initial parameters and the selected parameters in the highest ranked models (TLL >−140).

## Discussion

**Fig 7:**
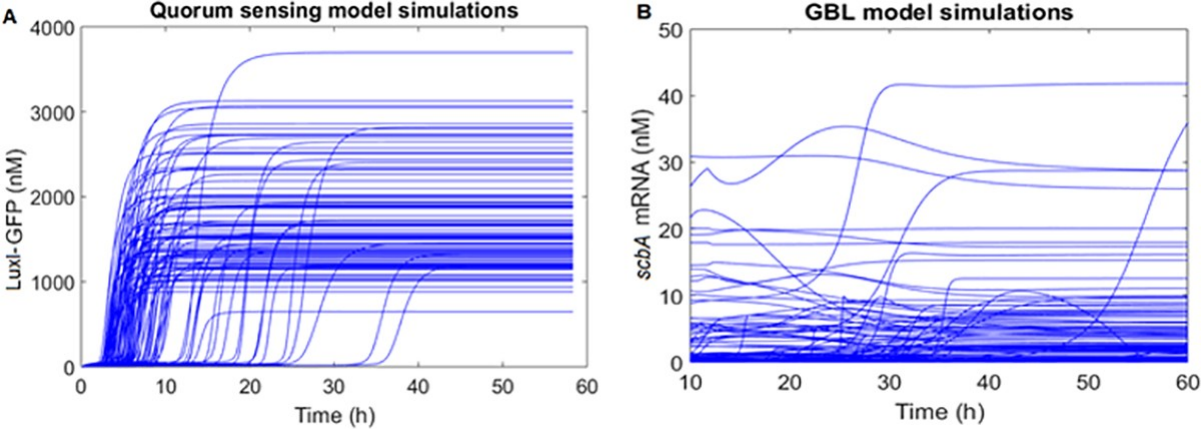
Outputs from ensemble modelling simulations on the LuxI protein from the quorum sensing (QS) model (A) by Weber *et al*.[25] and the *scbA* mRNA of the GBL model (B). While QS produced consistent and robust modelling results, the GBL model was very unpredictable and different sets of parameters led to completely different behaviours.

The simulation of the GBL model in all 8 regulatory scenarios revealed a surprisingly volatile system (**Fig 7**) with different sets of plausible parameters leading to completely different behaviors (e.g., a peak and decline vs. a smooth increase). A large number of models in all scenarios achieved very low to non-calculable likelihood scores, i.e. their predictions had no resemblance to the experimental observations. This is in striking contrast to the quorum sensing circuits[25] which, with a very similar topology achieve a remarkably robust model behavior when modelled computationally (**Fig 7**). The rather obvious explanation for this difference in behavior is that in this kind of complex non-linear system small variations in topology (and parameter values) can lead to major differences in emergent behavior. Although the reason for the different behavior of the GBL model is not entirely clear, it is biologically plausible: the filamentous nature of the actinomycetes entails a different signaling paradigm, and consequently both the experimental data and the model predictions indicate that GBL signaling shows more similarity with an endocrine signaling mechanism, rather than an AHL-like quorum sensing system. It therefore becomes obvious that our understanding of the molecular mechanisms underlying the GBL system is still incomplete, and that none of the proposed mechanism can fully and satisfactorily explain the circuit behavior, alone or in combination.

Nevertheless, the systematic investigation of the different scenarios elucidated some important features of the system and revealed behaviors that cannot be explained by any combination of plausible parameter values. The hypothesis of a putative ScbR–ScbA complex playing an important regulatory role is not supported by the available evidence, as it only adds to the complexity of the model without actually contributing to its quality. Of course that does not exclude the possibility that it plays an important role in other conditions or with respect to systems variables that were not measured in the experiments available. Similarly, transcriptional interference by itself or combined with the ScbR protein being an activator (R_act_ mechanism), does not sufficiently explain the experimental results. However, when any of the scenarios is combined with the antisense RNA mechanism, the number of successful models is clearly enhanced, suggesting that this mechanism is critical for the observed behavior of the system in the experimental conditions analysed.

Additionally, including the R_act_ mechanism in the models improved the prediction of *scbA* transcript dynamics, but at the cost of the *scbR* mRNA predictions. This suggests that, although the modelling results do not support the idea that ScbR is activating *scbA* transcription, there probably is an unidentified activator involved; this unknown activator could also explain the difference in the peak width of the two mRNAs, which none of the scenarios so far managed to sufficiently reproduce.

The relative promoter strengths seem to also significantly contribute to the log likelihood scores of the models, with the *scbR* promoter seemingly being 3–8 times more aggressive (${k_{F}}_{R}>{k_{F}}_{A}$) in the most successful models than its *scbA* counterpart. This result is supported by recent experimental data[8], which show that *scbR* has the strongest promoter in the GBL system, followed by the promoter of the CPK cluster and finally of *scbA*. Furthermore, the model suggests that the affinity of ScbR for the *scbA* promoter, seems to be higher than for *scbR*, in agreement with the results of a previously published DNase protection assay.[17]

The parameter analysis revealed that diffusion does not seem to majorly affect the GBL model. On the other hand, the importance of the degradation of ScbA protein and the synthesis of γ-butyrolactones seemed to be a recurring issue in most groups of simulations. Finally, growth seemed to also significantly affect the model, with the lag phase (λ) playing a prominent role in all scenarios, followed by the maximum growth rate (μ_max_).

These findings suggest that the GBL system behavior does not stem from a population-wide regulation (despite the similarities of the circuitry to well-known quorum sensing systems), but from a growth-dependent response of the system to its external environment. If the diffusion of the SCBs is not an important factor for the model behavior, it might be possible that intercellular bacterial communication is not actually involved and there is very limited coordination within the colony to trigger the antibiotic production, but the transition is performed by the cells individually once they reach their stationary phase, at least under the laboratory conditions used in our reference experiments. Additional experiments and simulations will need to be performed in order to fully clarify the role of the two genes and their interactions, as well as the existence of another activating agent and the stability of ScbR and ScbA proteins. An option to test the existence and significance of the antisense RNA and transcriptional interference mechanisms would be to conduct a series of experiments (and simulations) using synthetic genetic circuits, with the *scbR* and *scbA* promoters uncoupled and coupled, and with either one or both of the genes being replaced by reporter constructs that lack the scbA/R functionality.

Unquestionably, the availability of more experimental data would also greatly assist in the further validation and improvement of the model (within the limitations imposed by the inherent “sloppiness” of the system;[36] **S5 Appendix**). Additional quantitative transcriptomics results for *scbA* and *scbR* genes could validate the difference in the width of the two peaks, and more precise measurements on the degradation rates of the ScbA and ScbR proteins would help to fine-tune the probability distributions for these parameters and assess the biological plausibility of the previously suggested mechanisms. Finally, quantitative proteomics results from an experiment where cells do not produce γ-butyrolactones but are added externally in different concentrations would also be of interest, as it would assist with the validation of the model in a protein level additional to the mRNA level.

Previous studies have shown that in a system involving a small number of molecules, such as a regulatory or signaling system, stochasticity (fluctuations in transcription and translation or randomness in the autoinducer diffusion from the cell to the environment) can have a significant impact on the switch induction.[37, 38] Therefore, the small-size GBL system provides a good opportunity for stochastic modelling, in order to study the sensitivity of this system to internal or external fluctuations in the future. Furthermore, the stochastic analysis could reveal more information on the type of communication (if any) that takes place within a *Streptomyces* colony. The stochastic modelling should be able to represent the heterogeneity arising from intrinsic or extrinsic noise and thus achieve a more realistic description of key properties of the system, such as population-wide bet hedging.

The improved GBL model clarified some aspects of the system, but also raised some interesting questions. However, most importantly, it became clear that the model can now be used as a versatile and adaptable tool which will challenge and refine our understanding of the proposed functioning of this system, and perhaps even suggest a different biological role than originally envisaged. The developed framework of analysis with the explicit consideration and documentation of uncertainty will now form the basis for a further extension of the model using alternative topologies and will allow us to quantify our posterior belief about the model’s parameters in the face of new experimental data. Finally, the model indicates key experiments, which could more completely elucidate the role of the system and the interactions of its components and potentially lead to the design of reliable and sensitive systems with significant applications as orthologous regulatory circuits in synthetic biology and biotechnology.

## Supporting information

S1 Appendix Model design and analysis. (PDF)

S2 Appendix Model results data. (XLSX)

S3 Appendix GBL Models. (PDF)

S4 Appendix Supplementary Figures. (PDF)

S5 Appendix Model uncertainty in sloppy models. (PDF)
