# Supplementary material for: Unravelling the γ-butyrolactone network in Streptomyces coelicolor by computational ensemble modelling
Source: PLoS Comput Biol. 2020 Jul 10;16(7):e1008039. doi: 10.1371/journal.pcbi.1008039 (PMC7384680; doi:10.1371/journal.pcbi.1008039)
Supplement: S1 Appendix — (PDF) [file pcbi.1008039.s001.pdf]

S1 Appendix

Supplementary Tables

Supplementary Table St1: List of species for the ScbR/ScbA model

Name	Description	Compartment
O_R	Operator site of ScbR upstream *scbR* promoter	Cell
O_A	Operator site of ScbR within *scbA* promoter as well as putative operator site O_A' of AR_2 complex	Cell
O_R•R_2	Complex of R protein and operator site upstream *scbR* promoter	Cell
O_R•2R_2	Complex of two R proteins and operator site upstream *scbR* promoter	Cell
O_A•R_2	Complex of R protein and operator site within *scbA* promoter	Cell
O_A•2R_2	Complex of two R proteins and operator site within *scbA* promoter	Cell
O_A•AR_2	Complex of putative operator site presumed to activate *scbA* gene and AR_2	Cell
O_A•R_2•AR_2	Complex of operator site within *scbA* promoter with AR_2 and R_2	Cell
O_A•2R_2•AR_2	Complex of operator site within *scbA* promoter with AR_2 and two R_2 molecules	Cell
r	mRNA transcript of *scbR* gene	Cell
a	mRNA transcript of *scbA* gene	Cell
r•a	Complex of full length *scbR* and *scbA* mRNAs due to antisense effect	Cell
R	ScbR protein	Cell
R_2	ScbR homo-dimer	Cell
A	ScbA protein	Cell
C	SCB (γ-butyrolactone)	Cell
AR_2	Putative ScbA-ScbR complex	Cell
S	Glycerol derivative and β-keto acid derivative precursors	Cell
C_2•R_2	SCBs-ScbR complex	Cell
C_e	Extracellular SCB (γ-butyrolactone)	Environment

Supplementary Table St2: Chemical reactions for the ScbR/ScbA model

$O_R + R_2 \rightleftharpoons O_R \bullet R_2$	(1)
$O_R \bullet R_2 + R_2 \rightleftharpoons O_R \bullet 2R_2$	(2)
$O_A + R_2 \rightleftharpoons O_A \bullet R_2$	(3)
$O_A \bullet R_2 + R_2 \rightleftharpoons O_A \bullet 2R_2$	(4)
$A + R_2 \rightleftharpoons AR_2$	(5)[*1]
$2C + R_2 \rightleftharpoons C_2 \bullet R_2$	(6)
$O_A + AR_2 \rightleftharpoons O_A \bullet AR_2$	(7)[*1]
$O_A \bullet R_2 + AR_2 \rightleftharpoons O_A \bullet R_2 \bullet AR_2$	(8)[*1]
$O_A \bullet 2R_2 + AR_2 \rightleftharpoons O_A \bullet 2R_2 \bullet AR_2$	(9)[*1]
$O_A \bullet AR_2 + R_2 \rightleftharpoons O_A \bullet R_2 \bullet AR_2$	(10)[*1]

$O_A{\bullet}R_2{\bullet}AR_2+R_2 \rightleftharpoons O_A{\bullet}2R_2{\bullet}AR_2$	(11)[*1]
$\mathbf{A}\ (\mathbf{S}) \rightarrow \mathbf{A} + \mathbf{C}$	(12)
$O_R \rightarrow O_R + r$	(13)
$O_A \rightarrow O_A + a$	(14)
$O_A{\bullet}AR_2 \rightarrow O_A{\bullet}AR_2 + a$	(15)[*1]
$O_A{\bullet}R_2 \rightarrow O_A{\bullet}R_2 + a$	(16)[*3]
$O_A{\bullet}2R_2 \rightarrow O_A{\bullet}2R_2 + a$	(17)[*3]
$O_A{\bullet}R_2{\bullet}AR_2 \rightarrow O_A{\bullet}R_2{\bullet}AR_2 + a$	(18)[*3]
$O_A{\bullet}2R_2{\bullet}AR_2 \rightarrow O_A{\bullet}2R_2{\bullet}AR_2 + a$	(19)[*3]
$a + r \rightleftharpoons r{\bullet}a$	(20)[*2]
$r \rightarrow r + R$	(21)
$a \rightarrow a + A$	(22)
$2R \rightleftharpoons R_2$	(23)
$\mathbf{C} \rightleftharpoons \mathbf{C_e}$	(24)
$r \rightarrow \emptyset$	(25)
$R \rightarrow \emptyset$	(26)
$R_2 \rightarrow \emptyset$	(27)
$a \rightarrow \emptyset$	(28)
$A \rightarrow \emptyset$	(29)
$C \rightarrow \emptyset$	(30)
$C_2{\bullet}R_2 \rightarrow \emptyset$	(31)
$AR_2 \rightarrow \emptyset$	(32)[*1]
$r{\bullet}a \rightarrow \emptyset$	(33)[*2]
$C_e \rightarrow \emptyset$	(34)
$O_R{\bullet}2R_2 \rightarrow O_R{\bullet}2R_2 + O_R$	(35)
$O_R{\bullet}R_2 \rightarrow O_R{\bullet}R_2 + O_R$	(36)
$O_A{\bullet}R_2 \rightarrow O_A{\bullet}R_2 + O_A$	(37)
$O_A{\bullet}2R_2 \rightarrow O_A{\bullet}2R_2 + O_A$	(38)
$O_A{\bullet}AR_2 \rightarrow O_A{\bullet}AR_2 + O_A$	(39)[*1]
$O_A{\bullet}R_2{\bullet}AR_2 \rightarrow O_A{\bullet}R_2{\bullet}AR_2 + O_A$	(40)[*1]
$O_A{\bullet}2R_2{\bullet}AR_2 \rightarrow O_A{\bullet}2R_2{\bullet}AR_2 + O_A$	(41)[*1]

*1 Reactions (5), (7)–(11), (15) , (32) and (39)–(41) occur only in scenarios B, E, F and H, which include the activating complex AR_2.

*2 Reactions (20) and (33) occur only in scenarios C, E, G and H, which include the antisense RNA interactions

*3 Reactions (16)–(19) occur only in scenarios D, F, G and H, which include the activation of *scbA* by R_2

Supplementary Table St3: Differential equations for the ScbR/ScbA model

$$\frac{d[O_R]}{dt} = \mu \cdot [O_R \bullet R_2] - \frac{k_1^-}{K_{d1}} \cdot [O_R] \cdot [R_2] + k_1^- \cdot [O_R \bullet R_2] + \mu \cdot [O_R \bullet 2R_2]$$

$$\frac{d[O_A]}{dt} = \mu \cdot [O_A \bullet R_2] + \mu \cdot [O_A \bullet AR_2] + \mu \cdot [O_A \bullet 2R_2] + \mu \cdot [O_A \bullet R_2 \bullet AR_2] + \mu \cdot [O_A \bullet 2R_2 \bullet AR_2] - \frac{k_2^-}{K_{d2}} \cdot [O_A] \cdot [R_2] + k_2^- \cdot [O_A \bullet R_2] - \frac{k_5^-}{K_{d5}} \cdot [O_A] \cdot [AR_2] + k_5^- \cdot [O_A \bullet AR_2]$$

$$\frac{d[O_R \bullet 2R_2]}{dt} = \frac{k_7^-}{K_{d7}} \cdot [O_R \bullet R_2] \cdot [R_2] - k_7^- \cdot [O_R \bullet 2R_2] - \mu \cdot [O_R \bullet 2R_2]$$

$$\frac{d[O_R \bullet R_2]}{dt} = \frac{k_1^-}{K_{d1}} \cdot [O_R] \cdot [R_2] - k_1^- \cdot [O_R \bullet R_2] - \frac{k_7^-}{K_{d7}} \cdot [O_R \bullet R_2] \cdot [R_2] + k_7^- \cdot [O_R \bullet 2R_2] - \mu \cdot [O_R \bullet R_2]$$

$$\frac{d[O_A \bullet R_2]}{dt} = \frac{k_2^-}{K_{d2}} \cdot [O_A] \cdot [R_2] - k_2^- \cdot [O_A \bullet R_2] - \mu \cdot [O_A \bullet R_2] - \frac{k_8^-}{K_{d8}} \cdot [O_A \bullet R2] \cdot [R_2] + k_8^- \cdot [O_A \bullet 2R_2] - \frac{k_9^-}{K_{d9}} \cdot [O_A \bullet R_2] \cdot [AR_2] + k_9^- \cdot [O_A \bullet R_2 \bullet AR_2]$$

$$\frac{d[O_A \bullet AR_2]}{dt} = \frac{k_5^-}{K_{d5}} \cdot [O_A] \cdot [AR_2] - k_5^- \cdot [O_A \bullet AR_2] - \mu \cdot [O_A \bullet AR_2] - \frac{k_{11}^-}{K_{d11}} \cdot [O_A \bullet AR_2] \cdot [R_2] + k_{11}^- \cdot [O_A \bullet R_2 \bullet AR_2]$$

$$\frac{d[r]}{dt} = T_R \cdot [O_R] - \frac{k_{ar}^-}{K_{ar}} [r] \cdot [a] + k_{ar}^- \cdot [r \bullet a] - d_{mR} \cdot [r] - \mu \cdot [r]$$

$$\frac{d[a]}{dt} = T_{A_{basal}} \cdot [O_A] + T_A \cdot [O_A] + T_A \cdot [O_A \bullet AR_2] + T_A \cdot [O_A \bullet R_2] + T_A \cdot [O_A \bullet 2R_2] + T_A \cdot [O_A \bullet R_2 \bullet AR_2] + T_A \cdot [O_A \bullet 2R_2 \bullet AR_2] - \frac{k_{ar}^-}{K_{ar}} \cdot [r] \cdot [a] + k_{ar}^- \cdot [r \bullet a] - d_{mA} \cdot [a] - \mu \cdot [a]$$

$$\frac{d[r \bullet a]}{dt} = \frac{k_{ar}^-}{K_{ar}} \cdot [r] \cdot [a] - k_{ar}^- \cdot [r \bullet a] - d_{mRA} \cdot [r \bullet a] + \mu \cdot [r \bullet a]$$

$$\frac{d[R]}{dt} = P_R \cdot [r] - \frac{k_6^-}{K_{d6}} \cdot [R]^2 + k_6^- \cdot [R_2] - d_R \cdot [R] - \mu \cdot [R]$$

$$\frac{d[R_2]}{dt} = \frac{k_6^-}{K_{d6}} \cdot [R]^2 - k_6^- \cdot [R_2] - \frac{k_1^-}{K_{d1}} \cdot [O_R] \cdot [R_2] + k_1^- \cdot [O_R \bullet R_2] - \frac{k_2^-}{K_{d2}} \cdot [O_A] \cdot [R_2] + k_2^- \cdot [O_A \bullet R_2] - \frac{k_3^-}{K_{d3}} \cdot [A] \cdot [R_2] + k_3^- \cdot [AR_2] - \frac{k_4^-}{K_{d4}} \cdot [R_2] \cdot [C]^2 + k_4^- \cdot [C_2 \bullet R_2] - \frac{k_7^-}{K_{d7}} \cdot [O_R \bullet R_2] \cdot [R_2] + k_7^- \cdot [O_R \bullet 2R_2] - d_{R2} \cdot [R_2] - \mu \cdot [R_2] - \frac{k_8^-}{K_{d8}} \cdot [O_A \bullet R_2] \cdot [R_2] + k_8^- \cdot [O_A \bullet 2R_2] - \frac{k_{11}^-}{K_{d11}} \cdot [O_A \bullet AR_2] \cdot [R_2] + k_{11}^- \cdot [O_A \bullet R_2 \bullet AR_2] - \frac{k_{12}^-}{K_{d12}} \cdot [O_A \bullet R_2 \bullet AR_2] \cdot [R_2] + k_{12}^- \cdot [O_A \bullet 2R_2 \bullet AR_2]$$

$$\frac{d[A]}{dt} = P_A \cdot [a] - \frac{k_3^-}{K_{d3}} \cdot [A] \cdot [R_2] + k_3^- \cdot [AR_2] - d_A \cdot [A] - \mu \cdot [A]$$

$$\frac{d[C]}{dt} = K_C \cdot [A] - \frac{k_4^-}{K_{d4}} \cdot [R_2] \cdot [C]^2 + k_4^- \cdot [C_2 \bullet R_2] - d_C \cdot [C] - \mu \cdot [C] + D \cdot ([C_e] - [C])$$

$$\frac{\mathbf{d}[\mathbf{AR}_2]}{dt} = \frac{k_3^-}{K_{d3}} \cdot [A] \cdot [\mathbf{R}_2] - k_3^- \cdot [\mathbf{AR}_2] - \frac{k_5^-}{K_{d5}} \cdot [\mathbf{O}_A] \cdot [\mathbf{AR}_2] + k_5^- \cdot [\mathbf{O}_A \bullet \mathbf{AR}_2] - \mathbf{d}_{AR} \cdot [\mathbf{AR}_2] - \mu \cdot [\mathbf{AR}_2] - \frac{k_9^-}{K_{d9}} \cdot [\mathbf{O}_A \bullet \mathbf{R}_2] \cdot [\mathbf{AR}_2] + k_9^- \cdot [\mathbf{O}_A \bullet \mathbf{R}_2 \bullet \mathbf{AR}_2] - \frac{k_{10}^-}{K_{10}} \cdot [\mathbf{O}_A \bullet 2\mathbf{R}_2] \cdot [\mathbf{AR}_2] + k_{10}^- \cdot [\,\mathbf{O}_A \bullet 2\mathbf{R}_2 \bullet \mathbf{AR}_2]$$

$$\frac{\mathbf{d}[C_2 \bullet \mathbf{R}_2]}{dt} = \frac{k_4^-}{K_{d4}} \cdot [\mathbf{R}_2] \cdot [C]^2 - k_4^- \cdot [C_2 \bullet \mathbf{R}_2] - \mathbf{d}_{CR} \cdot [C_2 \bullet \mathbf{R}_2] - \mu \cdot [C_2 \bullet \mathbf{R}_2]$$

$$\frac{\mathbf{d}[\mathbf{C_e}]}{dt} = \varrho \cdot D \cdot ([C] - [C_e]) - \mathbf{d}_C \cdot [C_e] + \varrho \cdot \mu \cdot [C_e]$$

$$\frac{\mathbf{d}[\mathbf{O}_A \bullet 2\mathbf{R}_2]}{dt} = \frac{k_8^-}{K_{d8}} \cdot [\mathbf{O}_A \bullet \mathbf{R2}] \cdot [\mathbf{R}_2] - k_8^- \cdot [\mathbf{O}_A \bullet 2\mathbf{R}_2] - \frac{k_{10}^-}{K_{10}} \cdot [\mathbf{O}_A \bullet 2\mathbf{R}_2] \cdot [\mathbf{AR}_2] + k_{10}^- \cdot [\,\mathbf{O}_A \bullet 2\mathbf{R}_2 \bullet \mathbf{AR}_2] - \mu \cdot [\mathbf{O}_A \bullet 2\mathbf{R}_2]$$

$$\frac{\mathbf{d}[\mathbf{O}_A \bullet \mathbf{R}_2 \bullet \mathbf{AR}_2]}{dt} = \frac{k_9^-}{K_{d9}} \cdot [\mathbf{O}_A \bullet \mathbf{R}_2] \cdot [\mathbf{AR}_2] - k_9^- \cdot [\mathbf{O}_A \bullet \mathbf{R}_2 \bullet \mathbf{AR}_2] + \frac{k_{11}^-}{K_{d11}} \cdot [\mathbf{O}_A \bullet \mathbf{AR}_2] \cdot [\mathbf{R}_2] - k_{11}^- \cdot [\mathbf{O}_A \bullet \mathbf{R}_2 \bullet \mathbf{AR}_2] - \frac{k_{12}^-}{K_{d12}} \cdot [\mathbf{O}_A \bullet \mathbf{R}_2 \bullet \mathbf{AR}_2] \cdot [R_2] + k_{12}^- \cdot [\mathbf{O}_A \bullet 2\mathbf{R}_2 \bullet \mathbf{AR}_2] - \mu \cdot [\mathbf{O}_A \bullet \mathbf{R}_2 \bullet \mathbf{AR}_2]$$

$$\frac{\mathbf{d}[\mathbf{O}_A \bullet 2\mathbf{R}_2 \bullet \mathbf{AR}_2]}{dt} = \frac{k_{10}^-}{K_{10}} \cdot [\mathbf{O}_A \bullet 2\mathbf{R}_2] \cdot [\mathbf{AR}_2] - k_{10}^- \cdot [\,\mathbf{O}_A \bullet 2\mathbf{R}_2 \bullet \mathbf{AR}_2] + \frac{k_{12}^-}{K_{d12}} \cdot [\mathbf{O}_A \bullet \mathbf{R}_2 \bullet \mathbf{AR}_2] \cdot [R_2] - k_{12}^- \cdot [\mathbf{O}_A \bullet 2\mathbf{R}_2 \bullet \mathbf{AR}_2] - \mu \cdot [\mathbf{O}_A \bullet 2\mathbf{R}_2 \bullet \mathbf{AR}_2]$$

Supplementary Table St4: Information on the probability distributions for the model parameters

Parameter	Description	Units	Mode	Spread	Location parameter (μ)	Scale parameter (σ)
K_{d1}	Dissociation constant for binding of R_2 to O_R	nM	0.299	6.8	0.15471	1.1661
K_{d7}	Dissociation constant for binding of R_2 to O_R•R_2	nM	0.299	6.8	0.15471	1.1661
k^-_1	Dissociation rate for binding of R_2 to O_R	min^{-1}	0.489	1.98	−0.37642	0.58225
k^-_7	Dissociation rate for binding of R_2 to O_R•R_2	min^{-1}	0.489	1.98	−0.37642	0.58225
K_{d2}	Dissociation constant for binding of R_2 to O_A	nM	0.299	6.8	0.15471	1.1661
K_{d8}	Dissociation constant for binding of R_2 to O_A•R_2	nM	0.299	6.8	0.15471	1.1661
k^-_2	Dissociation rate for binding of R_2 to O_A	min^{-1}	0.489	1.98	−0.37642	0.58225
k^-_8	Dissociation rate for binding of R_2 to O_A•R_2	min^{-1}	0.489	1.98	−0.37642	0.58225

K_{d3}	Dissociation constant for binding of R_2 to A	nM	$9.9 \cdot 10^4$	10.02	13.192	1.2977
k^-_3	Dissociation rate for binding of R_2 to A	min^{-1}	1879.7	23.13	9.9423	1.5503
K_{d4}	Dissociation constant for binding of C to R_2	nM	1.58	730.63	5.9389	2.3412
k^-_4	Dissociation rate for binding of C to R_2	min^{-1}	0.816	244	4.2872	2.119
K_{d5}	Dissociation constant for binding of AR_2 to O_A	nM	0.299	6.8	0.15471	1.1661
K_{d9}	Dissociation constant for binding of AR_2 to $O_A \bullet R_2$	nM	0.299	6.8	0.15471	1.1661
K_{d10}	Dissociation constant for binding of AR_2 to $O_A \bullet 2R_2$	nM	0.299	6.8	0.15471	1.1661
K_{d11}	Dissociation constant for binding of R_2 to $O_A \bullet AR_2$	nM	0.299	6.8	0.15471	1.1661
K_{d12}	Dissociation constant for binding of R_2 to $O_A \bullet R_2 \bullet AR_2$	nM	0.299	6.8	0.15471	1.1661
k^-_5	Dissociation rate for binding of AR_2 to O_A	min^{-1}	0.489	1.98	−0.37642	0.58225
k^-_9	Dissociation rate for binding of AR_2 to $O_A \bullet R_2$	min^{-1}	0.489	1.98	−0.37642	0.58225
k^-_{10}	Dissociation rate for binding of AR_2 to $O_A \bullet 2R_2$	min^{-1}	0.489	1.98	−0.37642	0.58225
k^-_{11}	Dissociation rate for binding of R_2 to $O_A \bullet AR_2$	min^{-1}	0.489	1.98	−0.37642	0.58225
k^-_{12}	Dissociation rate for binding of R_2 to $O_A \bullet R_2 \bullet AR_2$	min^{-1}	0.489	1.98	−0.37642	0.58225
K_C	Synthesis rate of C	min^{-1}	0.094	60.49	0.87914	1.8017
Ω_R	Strength of the O_R promoter/ Transcription rate constant of the isolated O_R promoter	min^{-1}	0.8346	3.55	0.63056	0.90076
k_F	Firing rate (elongation initiation rate) constant	min^{-1}	20.7	1.34	3.1107	0.28276
k_{onR}	Rate of RNA polymerase binding to the O_R promoter	min^{-1}	0.867	4.09	0.78694	0.96428
Ω_{Abasal}	Basal transcription rate constant of the isolated O_A promoter	min^{-1}	0.0037	10.93	−3.8324	1.3259
Ω_A	Strength of the O_A promoter/ Transcription rate constant of the isolated O_A promoter	min^{-1}	0.572	3.5	0.2514	0.89956
k_{onA}	Rate of RNA polymerase binding to the O_A promoter	min^{-1}	0.587	3.875	0.35255	0.94054
$k_{onAbasal}$	Basal rate of RNA polymerase binding to the O_A promoter	min^{-1}	0.00373	10.97	−3.8297	1.3269

K_{ar}	Dissociation rate for binding of *r* to *a*	nM	7.8	7.27	3.4665	1.1882
k^{-}_{ar}	Dissociation rate for binding of *r* to *a*	min^{-1}	0.223	4.8	−0.43359	1.0326
P_R	Translation rate of R	min^{-1}	0.744	3.2	0.42952	0.85176
P_A	Translation rate of A	min^{-1}	0.5	3.2	0.053356	0.85327
K_{d6}	Dissociation constant for R_2 (homo-dimer) formation	nM	3.89	1.9	1.6716	0.55779
k^{-}_{6}	Dissociation rate for R_2 (homo-dimer) formation	min^{-1}	1.1997	21.7	2.5303	1.5324
d_{mR}	Degradation rate of *r*	min^{-1}	0.14	2.06	−1.5916	0.60824
d_R	Degradation rate of R	min^{-1}	0.00144	1.78	−5.4676	0.9222
d_{R2}	Degradation rate of R_2	min^{-1}	0.00144	1.78	−5.4676	0.9222
d_{mA}	Degradation rate of *a*	min^{-1}	0.14	2.06	−1.5916	0.60824
d_A	Degradation rate of A	min^{-1}	0.00144	1.78	−5.4676	0.9222
d_C	Degradation rate of C	min^{-1}	0.0032	5.1	−4.6234	1.0539
d_{CR}	Degradation rate of $C_2{\bullet}R_2$	min^{-1}	0.00144	1.78	−6.2837	0.50981
d_{AR}	Degradation rate of AR_2	min^{-1}	0.00144	1.78	−6.2837	0.50981
d_{mRA}	Degradation rate of antisense RNA complex *r•a*	min^{-1}	4.9	14.1	2.5669	0.98835
D	Diffusion rate of C through the cell membrane	min^{-1}	2.8	4.27	1.9947	0.98233

Supplementary Table St5: Quantification of the quality criteria for the comparison between simulations and transcriptomics data

Criteria	Quantification formula	Value (μ)	Uncertainty (σ)
T_{max}	Time at peak (minutes)	1320	150
Relative gain of *scbR*	$[R_{peak}]-[R_{min^a}]$	4.4	1.51
Relative gain of *scbA*	$[A_{peak}]-[A_{min^a}]$	5.27	1.678
Relative loss of *scbR*	$\frac{[R_{min^b}]-[R_{min^a}]}{[R_{peak}]-[R_{min^a}]}$	0.251	0.13
Relative loss of *scbA*	$[A_{peak}]-[A_{min^b}]$	4.567	0.433
Slope of increase of *scbR*	$\frac{[R_{peak}]-[R_{min^a}]}{[Time_{R_{peak}}]-[Time_{R_{min^a}}]}$	2.51	1.565
Slope of increase of *scbA*	$\frac{[A_{peak}]-[A_{min^a}]}{[Time_{A_{peak}}]-[Time_{A_{min^a}}]}$	3.13	1.835
Slope of decrease of *scbR*	$\frac{[R_{peak}]-[R_{min^b}]}{2}$	1.144	0.653
Slope of decrease of *scbA*	$\frac{[A_{min^b}]-[A_{peak}]}{[Time_{A_{min^b}}]-[Time_{A_{peak}}]}$	−2.748	0.681
Production of butyrolactones	Maximum amount produced (nM)	Mode=250 μ=5.8687	Spread=2 σ=0.5892

R_{min}^{a} : minimum concentration of *scbR* before the peak
A_{min}^{a} : minimum concentration of *scbA* before the peak
R_{min}^{b} : minimum concentration of *scbR* after the peak
A_{min}^{a} : minimum concentration of *scbA* after the peak

State of promoters and transcription

The mechanisms involved in the transcription of *scbA* and *scbR* are defined in reactions (1)–(4), (7)–(11) and (13)–(19) of **Supplementary Table St2**. In order to describe the overlapping promoter effects, the transcription reactions of each gene need to take into account the strength and the state of the gene's promoter (free or occupied), as well as the potential interference from the transcription of the opposite gene. In order to accomplish this, the mathematical model for overlapping promoters proposed by Bendtsen *et al.*[24] was employed. In our reactions, the transcription process depends on the transcription rates T_R and T_A. These are derived via the equations (1)–(8) by employing the promoter firing rates (k_{FR}, k_{FA}), the strength of the promoters (Ω_R, Ω_A), the occupancy of the promoters (θ_R, θ_A) and the rate of RNP binding on the promoters (k_{on_R}, k_{on_A}), as described below.

The model assumes that the strength of each promoter in the isolated state (uncoupled promoters) is equal to the number of RNA transcripts produced per unit of time. Therefore, the parameters Ω_R and Ω_A are set to be equal to the transcription rate constants of the isolated promoters. In order to describe the difference in the promoter strengths (i.e. RNAP firing rates), the general firing rate k_F is initially set to be common for both promoters, in accordance with the literature values. Afterwards, a heterogeneity factor (χ) is employed to define the difference between the two individual firing rates, as per: $k_{F_R} = k_F \cdot \chi$ and $k_{F_A} = \frac{k_F}{\chi}$. The promoters can be assumed to have equal strength in the isolated state (χ = 1), so their firing rates are equal ($k_{F_R} = k_{F_A} = k_F$). Alternatively, χ can have values in the range between 0.1 and 1 in order for *scbA* promoter to be more aggressive, or the reciprocal values (1 to 10) in order to reinforce *scbR* promoter. In order to include the strength of the promoter in the derivation of the transcription rate, the following formulas are used:

$$\frac{1}{k_{on_R}} = \frac{1}{\Omega_R} - \frac{1}{k_{F_R}} \quad (1)$$

$$\frac{1}{k_{on_A}} = \frac{1}{\Omega_A} - \frac{1}{k_{F_A}} \quad (2)$$

The maximal occupancy for the isolated *scbR* promoter can be calculated as per:

$$\theta_R^o = \frac{k_{onR}}{k_{onR} + k_{F_R}} \quad (3)$$

$$\theta_A^o = \frac{k_{onA}}{k_{onA} + k_{F_A}} \quad (4)$$

The promoter aspect ratios α_R and α_A can then be derived from the equations:

$$\alpha_R = \frac{k_{onR}}{k_{F_R}} = \frac{\theta_R^o}{1 - \theta_R^o} \quad (5)$$

$$\alpha_A = \frac{k_{onA}}{k_{F_A}} = \frac{\theta_A^o}{1 - \theta_A^o} \quad (6)$$

In our case, as the two promoters are overlapping, there will be three possibilities: a) The *scbR* promoter is occupied by the RNAP (probability=θ_R); b) the *scbA* promoter is occupied by the RNAP (probability=θ_A); c) both promoters are unoccupied (probability=$1-\theta_R-\theta_A$). Therefore, the occupancy for the coupled *scbR* and *scbA* promoters (θ_R, θ_A) is:

$$\theta_R = \frac{\alpha_R + 1}{\alpha_R + \alpha_A + 1} \quad (7)$$

$$\theta_A = \frac{\alpha_A + 1}{\alpha_R + \alpha_A + 1} \quad (8)$$

and the final transcription rate constant that is used for the model ODEs can be calculated as per: $T_R = k_{F_R} \cdot \theta_R \cdot \theta_R^o = \Omega_R \cdot \theta_R$ and $T_A = k_{F_A} \cdot \theta_A \cdot \theta_A^o = \Omega_A \cdot \theta_A$.

As shown in reactions (13) and (14), in the general case, the two genes are transcribed at the maximal rate when they are not repressed by the ScbR homo-dimer (species O_A and O_R). The transcription stops when even one homo-dimer binds to the gene operator ($O_R \bullet R_2$ and $O_A \bullet R_2$) and the suppression is further ensured by the binding of a second homo-dimer ($O_R \bullet 2R_2$ and $O_A \bullet 2R_2$) at a different time point (reactions (1)–(4)). The opposite occurs for *scbA* transcription in the scenarios involving ScbR being an activator for the gene. In these cases reaction (14) only accounts for transcription at a basal rate. Once either a single or two ScbR homodimers bind to the operator, maximal transcription is activated (reactions (16)–(17)).

In the mechanisms that include the putative ScbA–ScbR complex (AR_2), the maximal rate can also be achieved by having the AR_2 complex bind to O_A' (O_A•AR_2) (reactions (7) and (15)). In our model, O_A and O_A' are considered together under the general name O_A, which can be found in different states. The transcription is again suppressed when the ScbR homo-dimers bind to O_A (O_A•R_2•AR_2 and O_A•2R_2•AR_2), even though the activating complex may also be bound (reactions (8)-(11)). The suppression does not occur in the scenarios where ScbR is an activator, in which the complexes O_A•R_2•AR_2 and O_A•2R_2•AR_2 also lead to maximal transcription (reactions (18)–(19)).

Cell growth and division

As the experimental time simulated is over 60 hours, the effects of cell growth must be taken into consideration in addition to the various regulatory mechanisms. In our model, the number of cells is described by a six-parameter Baranyi–Roberts model,[25,26] which takes into account the lag phase by using an adjustment function. In this model, the natural logarithm of the cell concentration is:

$$y(t) = y_0 + \mu_{max}A(t) - \frac{1}{m}\ln\left(1 + \frac{e^{m\cdot\mu_{max}\cdot A(t)}-1}{e^{m(y_{max}-y_0)}}\right) \quad (9)$$

where $A(t) = t - \lambda + \frac{\ln(1-e^{-v\cdot t}+e^{-v(t-\lambda)})}{v}$, y_0 is the logarithm of the initial number of cells (inoculum), μ_{max} is the maximal growth rate, *m* is the curvature parameter (*m*=1 for logistic growth), λ is the lag phase and *v* is the rate limiting growth (usually assumed to be equal to μ_{max}). The specific growth rate (μ) is the first derivative of equation (9) and describes the change in the growth rate according to the cell population status. Additionally, we have to take into consideration the fact that the cells are still adapting to their new environment during the lag phase, so they do not produce any of the intracellular species of interest in a significant amount, and that the initial conditions defined in our model where all species except the DNA are set to zero, do not correspond to a natural state. For these reasons and in order to avoid over-fitting our model, we assume that the system is adjusting to the physiological conditions for the first 10-15 hours.

Cellular growth causes the dilution of the intracellular species concentrations at a rate of $-\mu \cdot C_x(t)$, where $C_x(t)$ is the concentration of a species *X* at time *t*. On the other hand, the genetic material is duplicated during cell division. This is taken into account in our model through reactions (35)–(41), where DNA concentration is increased at a rate of $+\mu \cdot (\text{Gene\_Operators}_{\text{bound}} + \text{Gene\_Operators}_{\text{free}})$, so that for DNA the cell growth dilution is compensated and the total DNA concentration is kept constant.

The volume of the culture medium is also affected by cellular growth. As the number of cells increases, the volume of the external compartment decreases. So, by defining V_{tot} as the total volume of the initial culture (medium and cells), which remains constant, the volume of the medium will be $V_{ext} = V_{tot} - V_{c,tot}$. The ratio of total cell volume to environment volume is $\rho = \frac{V_{c,tot}}{V_{ext}}$. The decrease in the external volume leads to an increase of the concentration of C_e at a rate of $+\mu \cdot \rho \cdot [C_e](t)$. This also affects the diffusion of the external butyrolactones back to the cytoplasm. For this reason, the diffusion rate constant (D) of the reverse part of reaction (24) is additionally multiplied by the variable ρ.

Analysis of simulation results

The analysis of the improved model was based on comparing the simulation results with transcriptomics data (**Supplementary Figure Sp2**) reported in the publication by Nieselt *et al.*[27] Since the transcriptomics data and the simulations results are reported in different (and, in the case of the transcriptomics results, arbitrary) units, and in order to compare their qualitative behaviour, the values for both were normalised as per: $X_{i,norm} = \frac{X_i - \bar{X}}{SD}$

As the GBL system exhibited very different and non-consistent behaviours under various parameter sets (**Figure 5**) the comparison with the experimental data by ranking the distance between each experimental time point and the corresponding simulation time point was not effective (e.g. models got discarded for reaching the peak with an hour difference from the experimental data, despite showing qualitatively very similar behavior). Therefore, in order to enable a meaningful comparison of predictions and experimental results, and to ensure that the model captures the key aspects of the behaviour of the two genes, a set of specific criteria was defined. The similarity between the behaviour of the *scbR* and *scbA* simulations and the transcriptomics data was determined by comparing five different aspects of the transcript profiles:

1. The time point where the maximum mRNA abundance is reached
2. The relative gain (distance between the initial concentration and the peak)
3. The slope of increase (how fast the concentration reaches the peak)
4. The relative loss (distance between the peak and the subsequent minimum concentration)
5. The slope of decrease (how fast the concentration declines after the peak)

As the transcription data are noisy and were collected only at discrete time points, rather than continuously, each of these values can only be estimated with limited certainty (**Supplementary**

Table St5). We again used probability distributions to express on the plausible range of expected behaviours of these two species. These distributions were then used to compute the log-likelihood of each model in our ensemble for each one of the five criteria and to rank it based on how close it matched the experimental data, as per: $LL_i = \log(\frac{1}{\sqrt{2\pi\sigma^2}} e^{-\frac{(x-\mu)^2}{2\sigma^2}})$, where LL_i is the log-likelihood for one of the five criteria, μ is the maximum likelihood value for this criterion, σ is the standard deviation expressing the uncertainty around the value, and x is the simulation result being evaluated. Once the log-likelihood was determined for each of the quality criteria, the sum of the log-likelihoods was calculated for each species (SLL_R and SLL_A). As an additional criterion for the quality of the models, the maximum amount of butyrolactones produced was also considered. According to the literature, the activation threshold of the GBL circuit is around 250 nM, with the system becoming inhibited if much larger amounts than this accumulate (>1000 nM)[28]. A log-normal distribution with a mode of 250 nM and a multiplicative standard deviation of 2 was chosen to calculate the log-likelihood for this criterion (SLL_C), rather than a normal distribution as was done for the rest of the criteria. The total log-likelihood (TLL) for each model was derived by adding the total log-likelihoods of each of the two species and the log-likelihood for the GBL production (TLL= SLL_R+ SLL_A+ SLL_C).
