# Supplementary material for: Unravelling the γ-butyrolactone network in Streptomyces coelicolor by computational ensemble modelling
Source: PLoS Comput Biol. 2020 Jul 10;16(7):e1008039. doi: 10.1371/journal.pcbi.1008039 (PMC7384680; doi:10.1371/journal.pcbi.1008039)
Supplement: S3 Appendix — (PDF) [file pcbi.1008039.s003.pdf]

Scenario A

```
function dydt = GBLA(t, y)

% Units: nM, min, fl (um^3)
%Conversion coefficient molecules per cell -> nM
%nmolesV0 = 1*^9/6.02214*^23  *1/(V0*1*^-15);
%nmolesVext = 1*^9/6.02214*^23  *1/(Vext*1*^-15)
%Conversion of parameters from nM to molecules: 1.66054/V1 (cell),1.66054/Vext↙
(external)

%% first set Parameter values:

global kd1 kd2 kd3 kd4 kd5 kd6 kd7 kd8 kd9 kd10 kd11 kd12
global k_1 k_2 k_3 k_4 k_5 k_6 k_7 k_8 k_9 k_10 k_11 k_12
global konR WR;
global konA konA_basal WA WA_basal;
global kF chi chiset;
global Kar k_ar;
global dmR;
global dmA;
global PR;
global PA;
global dR dR2;
global dA;
global kC;
global dC;
global D;
global dCR;
global dAR;
global dmRA;

%% second set parameters values

global No N K l v m;
global u umax;
global r;
global Vo Vext Vtot Vc_tot Vx;
global TR TA TA_basal thetaR0 thetaR aR thetaA0 aA aA_basal thetaA thetaA0_basal↙
thetaA_basal kFA kFR;

%%

% chiset=1;
F=log(No)+umax.*(t-l+(log(1-exp(-v.*t)+exp(-v.*(t-l)))./v))-(1/m).*log(1+((exp(m.↙
*umax.*(t-l+(log(1-exp(-v.*t)+exp(-v.*(t-l)))./v)))-1)/(exp(m.*(log(K)-log(No))))));
N=exp(F);
u=umax.*((v.*exp(-t.*v) - v.*exp(v.*(l - t)))./(v.*(exp(v.*(l - t)) - exp(-t.*v) + 1))↙
+ 1) - (umax.*exp(m.*umax.*(t - l + log(exp(v.*(l - t)) - exp(-t.*v) + 1)./v))*exp(-m.↙
*(log(K) - log(No))).*((v.*exp(-t.*v) - v.*exp(v.*(l - t)))./(v.*(exp(v*(l - t)) - exp↙
(-t.*v) + 1)) + 1))./(exp(-m.*(log(K) - log(No))).*(exp(m.*umax.*(t - l + log(exp(v.*↙
(l - t)) - exp(-t.*v) + 1)./v)) - 1) + 1);

Vo=1.2;
Vc_tot=N*Vo;
Vtot=1.8e+15;
Vext=Vtot-Vc_tot;
Vx=Vext./Vc_tot;
r=1./Vx;
kFR=kF*chi;
% kFR=kF*chiset;
kFA=kF/chi;
```

```
% kFA=kF/chiset;
thetaR0=konR/(kFR+konR);
WR=kFR*thetaR0;
thetaA0=konA/(kFA+konA);
thetaA0_basal=konA_basal/(kFA+konA_basal);
WA=kFA*thetaA0;
WA_basal=kFA*thetaA0_basal;
aR=thetaR0/(1-thetaR0);
aA=thetaA0/(1-thetaA0);
aA_basal=thetaA0_basal/(1-thetaA0_basal);
thetaR=(aR+1)/(aR+aA+1);
thetaA=(aA+1)/(aR+aA+1);
thetaA_basal=(aA_basal+1)/(aR+aA_basal+1);
TR=kFR*thetaR*thetaR0;
TA=kFA*thetaA*thetaA0;
TA_basal=kFA*thetaA_basal*thetaA0_basal;

% Chemical Reactions and rate laws:
% OR = y(1)
% OA = y(2)
% OR-2R2 = y(3)
% OR-R2 = y(4)
% OA-R2 = y(5)
% OA-AR2 = y(6)
% r = y(7)
% a = y(8)
% r-a = y(9)
% R = y(10)
% R2 = y(11)
% A = y(12)
% C = y(13)
% AR2 = y(14)
% C2-R2 = y(15)
% Ce = y(16)
% OA-2R2 = y(17)
% OA-R2-AR2 = y(18)
% OA-2R2-AR2 = y(19)

%OR + R2 <-> OR-R2
r1 = (k_1/kd1).*y(1).*y(11)-k_1.*y(4);

%OA + R2 <-> OA-R2
r2 = (k_2/kd2).*y(2).*y(11)-k_2.*y(5);

%A + R2 <-> AR2
% r3 = (k_3/kd3).*y(12).*y(11)-k_3.*y(14);
r3=0;

%2C + R2 <-> C2-R2
r4 = (k_4/kd4).*y(11).*(y(13).^2)-k_4.*y(15);
% r4=0;

%OA + AR2 <-> OA-AR2
% r5 = (k_5/kd5).*y(2).*y(14)-k_5.*y(6);
r5=0;

%S (A) -> C
r6 = kC*y(12);

%OR -> OR + r
```

```
r7 = TR.*y(1);
% r7= WR*y(1);

%OR-R2 + R2 <-> OR-2R2
r8 = (k_7/kd7).*y(4).*y(11)-k_7.*y(3);
% r8=0;

%OA -> OA + a (max transcription 1 or basal transcription)
r9 = TA.*y(2);
% r9 = WA*y(2);
% r9 = 0;
% r9 = TA_basal*y(2);
% r9 = WA_basal*y(2);

%OA-AR2 -> OA-AR2 + a (max transcription 2)
% r10 = TA*y(6);
r10=0;
% r10 = WA*y(6);

%a + r <-> r-a
% r11 = (k_ar/Kar).*y(7).*y(8)-k_ar.*y(9);
r11=0;

%r -> r + R
r12 = PR.*y(7);

%a -> a + A
r13 = PA.*y(8);

%2R <-> R2
r14 = (k_6/kd6).*(y(10).^2)-k_6.*y(11);

%r ->
r15 = dmR.*y(7)+u.*y(7);

%R ->
r16 = dR.*y(10)+u.*y(10);

%R2 ->
r17 = dR2.*y(11)+u.*y(11);

%a ->
r18 = dmA.*y(8)+u.*y(8);

%A ->
r19 = dA.*y(12)+u.*y(12);

%C ->
r20 = dC.*y(13)+u.*y(13);

%C2-R2 ->
r21 = dCR.*y(15)+u.*y(15);

%AR2 ->
% r22 = dAR.*y(14)+u.*y(14);
r22=0;

%r-a ->
% r23 = dmRA.*y(9)+u.*y(9);
r23=0;
```

```
%Ce ->
r24 = dC.*y(16);

%C -> Ce
r25=D.*(y(16)-y(13));

%Ce -> C
r26=r.*D.*(y(13)-y(16));

%OR-2R2 -> OR-2R2 + OR
r29=u.*y(3);
% r29=0;

%OR-R2 -> OR-R2 + OR
r30=u.*y(4);
% r30=0;

%OA-R2 -> OA-R2 + OA
r31=u.*y(5);
% r31=0;

%OA-AR2 -> OA-AR2 + OA
% r32=u.*y(6);
r32=0;

% OA-R2 + R2 <-> OA-2R2
r33 = (k_8/kd8).*y(5).*y(11)-k_8.*y(17);
% r33=0;

% OA-R2 + AR2 <-> OA-R2-AR2
% r34 = (k_9/kd9).*y(5).*y(14)-k_9.*y(18);
r34=0;

% OA-2R2 + AR2 <-> OA-2R2-AR2
% r35 = (k_10/kd10).*y(17).*y(14)-k_10.*y(19);
r35=0;

% OA-AR2 + R2 <-> OA-R2-AR2
% r36 = (k_11/kd11).*y(6).*y(11)-k_11.*y(18);
r36=0;

% OA-R2-AR2 + R2 <-> OA-2R2-AR2
% r37 = (k_12/kd12).*y(18).*y(11)-k_12.*y(19);
r37=0;

% OA-2R2 -> OA-2R2 + OA
r38 = u.*y(17);
% r38=0;

% OA-R2-AR2 -> OA-R2-AR2 + OA
% r39 = u.*y(18);
r39=0;

% OA-2R2-AR2 -> OA-2R2-AR2 + OA
% r40 = u.*y(19);
r40=0;

% Correction for Ce: Negative dilution due to the total cells volume growth
r42=r.*u.*y(16);
```

```
%Differential equations:
dydt=[r30-r1+r29;... %OR
r31+r32+r38+r39+r40-r2-r5;... % OA
r8-r29;... % OR-2R2
r1-r8-r30;... % OR-R2
r2-r31-r33-r34;... % OA-R2
r5-r32-r36;... % OA-AR2
r7-r11-r15;... % r
r9+r10-r11-r18;... % a
r11-r23;... % r-a
r12-r14-r16;... % R
r14-r1-r2-r3-r4-r8-r17-r33-r36-r37;... % R2
r13-r3-r19;... % A
r6-r4-r20+r25;... % C
r3-r5-r22-r34-r35;... % AR2
r4-r21;... % C2-R2
r26-r24+r42;...  % Ce
r33-r35-r38;... % OA-2R2
r34+r36-r37-r39;... % OA-R2-AR2
r35+r37-r40]; %OA-2R2-AR2

end
```

Scenario B

```
function dydt = GBLB(t, y)

% Units: nM, min, fl (um^3)
%Conversion coefficient molecules per cell -> nM
%nmolesV0 = 1*^9/6.02214*^23  *1/(V0*1*^-15);
%nmolesVext = 1*^9/6.02214*^23  *1/(Vext*1*^-15)
%Conversion of parameters from nM to molecules: 1.66054/V1 (cell),1.66054/Vext↙
(external)

%% first set Parameter values:

global kd1 kd2 kd3 kd4 kd5 kd6 kd7 kd8 kd9 kd10 kd11 kd12
global k_1 k_2 k_3 k_4 k_5 k_6 k_7 k_8 k_9 k_10 k_11 k_12
global konR WR;
global konA konA_basal WA WA_basal;
global kF chi chiset;
global Kar k_ar;
global dmR;
global dmA;
global PR;
global PA;
global dR dR2;
global dA;
global kC;
global dC;
global D;
global dCR;
global dAR;
global dmRA;

%% second set parameters values

global No N K l v m;
global u umax;
global r;
global Vo Vext Vtot Vc_tot Vx;
global TR TA TA_basal thetaR0 thetaR aR thetaA0 aA aA_basal thetaA thetaA0_basal↙
thetaA_basal kFA kFR;

%%

chiset=1;
F=log(No)+umax.*(t-l+(log(1-exp(-v.*t)+exp(-v.*(t-l)))./v))-(1/m).*log(1+((exp(m.↙
*umax.*(t-l+(log(1-exp(-v.*t)+exp(-v.*(t-l)))./v)))-1)/(exp(m.*(log(K)-log(No))))));
N=exp(F);
u=umax.*((v.*exp(-t.*v) - v.*exp(v.*(l - t)))./(v.*(exp(v.*(l - t)) - exp(-t.*v) + 1))↙
+ 1) - (umax.*exp(m.*umax.*(t - l + log(exp(v.*(l - t)) - exp(-t.*v) + 1)./v))*exp(-m.↙
*(log(K) - log(No))).*((v.*exp(-t.*v) - v.*exp(v.*(l - t)))./(v.*(exp(v*(l - t)) - exp↙
(-t.*v) + 1)) + 1))./(exp(-m.*(log(K) - log(No))).*(exp(m.*umax.*(t - l + log(exp(v.*↙
(l - t)) - exp(-t.*v) + 1)./v)) - 1) + 1);

Vo=1.2;
Vc_tot=N*Vo;
Vtot=1.8e+15;
Vext=Vtot-Vc_tot;
Vx=Vext./Vc_tot;
r=1./Vx;
% kFR=kF*chi;
kFR=kF*chiset;
% kFA=kF/chi;
```

```
kFA=kF/chiset;
thetaR0=konR/(kFR+konR);
WR=kFR*thetaR0;
thetaA0=konA/(kFA+konA);
thetaA0_basal=konA_basal/(kFA+konA_basal);
WA=kFA*thetaA0;
WA_basal=kFA*thetaA0_basal;
aR=thetaR0/(1-thetaR0);
aA=thetaA0/(1-thetaA0);
aA_basal=thetaA0_basal/(1-thetaA0_basal);
thetaR=(aR+1)/(aR+aA+1);
thetaA=(aA+1)/(aR+aA+1);
thetaA_basal=(aA_basal+1)/(aR+aA_basal+1);
TR=kFR*thetaR*thetaR0;
TA=kFA*thetaA*thetaA0;
TA_basal=kFA*thetaA_basal*thetaA0_basal;

% Chemical Reactions and rate laws:
% OR = y(1)
% OA = y(2)
% OR-2R2 = y(3)
% OR-R2 = y(4)
% OA-R2 = y(5)
% OA-AR2 = y(6)
% r = y(7)
% a = y(8)
% r-a = y(9)
% R = y(10)
% R2 = y(11)
% A = y(12)
% C = y(13)
% AR2 = y(14)
% C2-R2 = y(15)
% Ce = y(16)
% OA-2R2 = y(17)
% OA-R2-AR2 = y(18)
% OA-2R2-AR2 = y(19)

%OR + R2 <-> OR-R2
r1 = (k_1/kd1).*y(1).*y(11)-k_1.*y(4);

%OA + R2 <-> OA-R2
r2 = (k_2/kd2).*y(2).*y(11)-k_2.*y(5);

%A + R2 <-> AR2
r3 = (k_3/kd3).*y(12).*y(11)-k_3.*y(14);
% r3=0;

%2C + R2 <-> C2-R2
r4 = (k_4/kd4).*y(11).*(y(13).^2)-k_4.*y(15);
% r4=0;

%OA + AR2 <-> OA-AR2
r5 = (k_5/kd5).*y(2).*y(14)-k_5.*y(6);
% r5=0;

%S (A) -> C
r6 = kC*y(12);

%OR -> OR + r
```

```
r7 = TR.*y(1);
% r7= WR*y(1);

%OR-R2 + R2 <-> OR-2R2
r8 = (k_7/kd7).*y(4).*y(11)-k_7.*y(3);
% r8=0;

%OA -> OA + a (max transcription 1 or basal transcription)
% r9 = TA.*y(2);
% r9 = WA*y(2);
% r9 = 0;
r9 = TA_basal*y(2);
% r9 = WA_basal*y(2);

%OA-AR2 -> OA-AR2 + a (max transcription 2)
r10 = TA*y(6);
% r10=0;
% r10 = WA*y(6);

%a + r <-> r-a
% r11 = (k_ar/Kar).*y(7).*y(8)-k_ar.*y(9);
r11=0;

%r -> r + R
r12 = PR.*y(7);

%a -> a + A
r13 = PA.*y(8);

%2R <-> R2
r14 = (k_6/kd6).*(y(10).^2)-k_6.*y(11);

%r ->
r15 = dmR.*y(7)+u.*y(7);

%R ->
r16 = dR.*y(10)+u.*y(10);

%R2 ->
r17 = dR2.*y(11)+u.*y(11);

%a ->
r18 = dmA.*y(8)+u.*y(8);

%A ->
r19 = dA.*y(12)+u.*y(12);

%C ->
r20 = dC.*y(13)+u.*y(13);

%C2-R2 ->
r21 = dCR.*y(15)+u.*y(15);

%AR2 ->
r22 = dAR.*y(14)+u.*y(14);
% r22=0;

%r-a ->
% r23 = dmRA.*y(9)+u.*y(9);
r23=0;
```

```
%Ce ->
r24 = dC.*y(16);

%C -> Ce
r25=D.*(y(16)-y(13));

%Ce -> C
r26=r.*D.*(y(13)-y(16));

%OR-2R2 -> OR-2R2 + OR
r29=u.*y(3);
% r29=0;

%OR-R2 -> OR-R2 + OR
r30=u.*y(4);
% r30=0;

%OA-R2 -> OA-R2 + OA
r31=u.*y(5);
% r31=0;

%OA-AR2 -> OA-AR2 + OA
r32=u.*y(6);
% r32=0;

% OA-R2 + R2 <-> OA-2R2
r33 = (k_8/kd8).*y(5).*y(11)-k_8.*y(17);
% r33=0;

% OA-R2 + AR2 <-> OA-R2-AR2
r34 = (k_9/kd9).*y(5).*y(14)-k_9.*y(18);
% r34=0;

% OA-2R2 + AR2 <-> OA-2R2-AR2
r35 = (k_10/kd10).*y(17).*y(14)-k_10.*y(19);
% r35=0;

% OA-AR2 + R2 <-> OA-R2-AR2
r36 = (k_11/kd11).*y(6).*y(11)-k_11.*y(18);
% r36=0;

% OA-R2-AR2 + R2 <-> OA-2R2-AR2
r37 = (k_12/kd12).*y(18).*y(11)-k_12.*y(19);
% r37=0;

% OA-2R2 -> OA-2R2 + OA
r38 = u.*y(17);
% r38=0;

% OA-R2-AR2 -> OA-R2-AR2 + OA
r39 = u.*y(18);
% r39=0;

% OA-2R2-AR2 -> OA-2R2-AR2 + OA
r40 = u.*y(19);
% r40=0;

% Correction for Ce: Negative dilution due to the total cells volume growth
r42=r.*u.*y(16);
```

```
%Differential equations:
dydt=[r30-r1+r29;... %OR
r31+r32+r38+r39+r40-r2-r5;... % OA
r8-r29;... % OR-2R2
r1-r8-r30;... % OR-R2
r2-r31-r33-r34;... % OA-R2
r5-r32-r36;... % OA-AR2
r7-r11-r15;... % r
r9+r10-r11-r18;... % a
r11-r23;... % r-a
r12-r14-r16;... % R
r14-r1-r2-r3-r4-r8-r17-r33-r36-r37;... % R2
r13-r3-r19;... % A
r6-r4-r20+r25;... % C
r3-r5-r22-r34-r35;... % AR2
r4-r21;... % C2-R2
r26-r24+r42;...  % Ce
r33-r35-r38;... % OA-2R2
r34+r36-r37-r39;... % OA-R2-AR2
r35+r37-r40]; %OA-2R2-AR2

end
```

Scenario C

```
function dydt = GBLC(t, y)

% Units: nM, min, fl (um^3)
%Conversion coefficient molecules per cell -> nM
%nmolesV0 = 1*^9/6.02214*^23  *1/(V0*1*^-15);
%nmolesVext = 1*^9/6.02214*^23  *1/(Vext*1*^-15)
%Conversion of parameters from nM to molecules: 1.66054/V1 (cell),1.66054/Vext↙
(external)

%% first set Parameter values:

global kd1 kd2 kd3 kd4 kd5 kd6 kd7 kd8 kd9 kd10 kd11 kd12
global k_1 k_2 k_3 k_4 k_5 k_6 k_7 k_8 k_9 k_10 k_11 k_12
global konR WR;
global konA konA_basal WA WA_basal;
global kF chi chiset;
global Kar k_ar;
global dmR;
global dmA;
global PR;
global PA;
global dR dR2;
global dA;
global kC;
global dC;
global D;
global dCR;
global dAR;
global dmRA;

%% second set parameters values

global No N K l v m;
global u umax;
global r;
global Vo Vext Vtot Vc_tot Vx;
global TR TA TA_basal thetaR0 thetaR aR thetaA0 aA aA_basal thetaA thetaA0_basal↙
thetaA_basal kFA kFR;

%%

chiset=1;
F=log(No)+umax.*(t-l+(log(1-exp(-v.*t)+exp(-v.*(t-l)))./v))-(1/m).*log(1+((exp(m.↙
*umax.*(t-l+(log(1-exp(-v.*t)+exp(-v.*(t-l)))./v)))-1)/(exp(m.*(log(K)-log(No))))));
N=exp(F);
u=umax.*((v.*exp(-t.*v) - v.*exp(v.*(l - t)))./(v.*(exp(v.*(l - t)) - exp(-t.*v) + 1))↙
+ 1) - (umax.*exp(m.*umax.*(t - l + log(exp(v.*(l - t)) - exp(-t.*v) + 1)./v))*exp(-m.↙
*(log(K) - log(No))).*((v.*exp(-t.*v) - v.*exp(v.*(l - t)))./(v.*(exp(v*(l - t)) - exp↙
(-t.*v) + 1)) + 1))./(exp(-m.*(log(K) - log(No))).*(exp(m.*umax.*(t - l + log(exp(v.*↙
(l - t)) - exp(-t.*v) + 1)./v)) - 1) + 1);

Vo=1.2;
Vc_tot=N*Vo;
Vtot=1.8e+15;
Vext=Vtot-Vc_tot;
Vx=Vext./Vc_tot;
r=1./Vx;
% kFR=kF*chi;
kFR=kF*chiset;
% kFA=kF/chi;
```

```
kFA=kF/chiset;
thetaR0=konR/(kFR+konR);
WR=kFR*thetaR0;
thetaA0=konA/(kFA+konA);
thetaA0_basal=konA_basal/(kFA+konA_basal);
WA=kFA*thetaA0;
WA_basal=kFA*thetaA0_basal;
aR=thetaR0/(1-thetaR0);
aA=thetaA0/(1-thetaA0);
aA_basal=thetaA0_basal/(1-thetaA0_basal);
thetaR=(aR+1)/(aR+aA+1);
thetaA=(aA+1)/(aR+aA+1);
thetaA_basal=(aA_basal+1)/(aR+aA_basal+1);
TR=kFR*thetaR*thetaR0;
TA=kFA*thetaA*thetaA0;
TA_basal=kFA*thetaA_basal*thetaA0_basal;

% Chemical Reactions and rate laws:
% OR = y(1)
% OA = y(2)
% OR-2R2 = y(3)
% OR-R2 = y(4)
% OA-R2 = y(5)
% OA-AR2 = y(6)
% r = y(7)
% a = y(8)
% r-a = y(9)
% R = y(10)
% R2 = y(11)
% A = y(12)
% C = y(13)
% AR2 = y(14)
% C2-R2 = y(15)
% Ce = y(16)
% OA-2R2 = y(17)
% OA-R2-AR2 = y(18)
% OA-2R2-AR2 = y(19)

%OR + R2 <-> OR-R2
r1 = (k_1/kd1).*y(1).*y(11)-k_1.*y(4);

%OA + R2 <-> OA-R2
r2 = (k_2/kd2).*y(2).*y(11)-k_2.*y(5);

%A + R2 <-> AR2
% r3 = (k_3/kd3).*y(12).*y(11)-k_3.*y(14);
r3=0;

%2C + R2 <-> C2-R2
r4 = (k_4/kd4).*y(11).*(y(13).^2)-k_4.*y(15);
% r4=0;

%OA + AR2 <-> OA-AR2
% r5 = (k_5/kd5).*y(2).*y(14)-k_5.*y(6);
r5=0;

%S (A) -> C
r6 = kC*y(12);

%OR -> OR + r
```

```
r7 = TR.*y(1);
% r7= WR*y(1);

%OR-R2 + R2 <-> OR-2R2
r8 = (k_7/kd7).*y(4).*y(11)-k_7.*y(3);
% r8=0;

%OA -> OA + a (max transcription 1 or basal transcription)
r9 = TA.*y(2);
% r9 = WA*y(2);
% r9 = 0;
% r9 = TA_basal*y(2);
% r9 = WA_basal*y(2);

%OA-AR2 -> OA-AR2 + a (max transcription 2)
% r10 = TA*y(6);
r10=0;
% r10 = WA*y(6);

%a + r <-> r-a
r11 = (k_ar/Kar).*y(7).*y(8)-k_ar.*y(9);
% r11=0;

%r -> r + R
r12 = PR.*y(7);

%a -> a + A
r13 = PA.*y(8);

%2R <-> R2
r14 = (k_6/kd6).*(y(10).^2)-k_6.*y(11);

%r ->
r15 = dmR.*y(7)+u.*y(7);

%R ->
r16 = dR.*y(10)+u.*y(10);

%R2 ->
r17 = dR2.*y(11)+u.*y(11);

%a ->
r18 = dmA.*y(8)+u.*y(8);

%A ->
r19 = dA.*y(12)+u.*y(12);

%C ->
r20 = dC.*y(13)+u.*y(13);

%C2-R2 ->
r21 = dCR.*y(15)+u.*y(15);

%AR2 ->
% r22 = dAR.*y(14)+u.*y(14);
r22=0;

%r-a ->
r23 = dmRA.*y(9)+u.*y(9);
% r23=0;
```

```
%Ce ->
r24 = dC.*y(16);

%C -> Ce
r25=D.*(y(16)-y(13));

%Ce -> C
r26=r.*D.*(y(13)-y(16));

%OR-2R2 -> OR-2R2 + OR
r29=u.*y(3);
% r29=0;

%OR-R2 -> OR-R2 + OR
r30=u.*y(4);
% r30=0;

%OA-R2 -> OA-R2 + OA
r31=u.*y(5);
% r31=0;

%OA-AR2 -> OA-AR2 + OA
% r32=u.*y(6);
r32=0;

% OA-R2 + R2 <-> OA-2R2
r33 = (k_8/kd8).*y(5).*y(11)-k_8.*y(17);
% r33=0;

% OA-R2 + AR2 <-> OA-R2-AR2
% r34 = (k_9/kd9).*y(5).*y(14)-k_9.*y(18);
r34=0;

% OA-2R2 + AR2 <-> OA-2R2-AR2
% r35 = (k_10/kd10).*y(17).*y(14)-k_10.*y(19);
r35=0;

% OA-AR2 + R2 <-> OA-R2-AR2
% r36 = (k_11/kd11).*y(6).*y(11)-k_11.*y(18);
r36=0;

% OA-R2-AR2 + R2 <-> OA-2R2-AR2
% r37 = (k_12/kd12).*y(18).*y(11)-k_12.*y(19);
r37=0;

% OA-2R2 -> OA-2R2 + OA
r38 = u.*y(17);
% r38=0;

% OA-R2-AR2 -> OA-R2-AR2 + OA
% r39 = u.*y(18);
r39=0;

% OA-2R2-AR2 -> OA-2R2-AR2 + OA
% r40 = u.*y(19);
r40=0;

% Correction for Ce: Negative dilution due to the total cells volume growth
r42=r.*u.*y(16);
```

```
%Differential equations:
dydt=[r30-r1+r29;... %OR
r31+r32+r38+r39+r40-r2-r5;... % OA
r8-r29;... % OR-2R2
r1-r8-r30;... % OR-R2
r2-r31-r33-r34;... % OA-R2
r5-r32-r36;... % OA-AR2
r7-r11-r15;... % r
r9+r10-r11-r18;... % a
r11-r23;... % r-a
r12-r14-r16;... % R
r14-r1-r2-r3-r4-r8-r17-r33-r36-r37;... % R2
r13-r3-r19;... % A
r6-r4-r20+r25;... % C
r3-r5-r22-r34-r35;... % AR2
r4-r21;... % C2-R2
r26-r24+r42;...  % Ce
r33-r35-r38;... % OA-2R2
r34+r36-r37-r39;... % OA-R2-AR2
r35+r37-r40]; %OA-2R2-AR2

end
```

Scenario D

```
function dydt = GBLD(t, y)

% Units: nM, min, fl (um^3)
%Conversion coefficient molecules per cell -> nM
%nmolesV0 = 1*^9/6.02214*^23  *1/(V0*1*^-15);
%nmolesVext = 1*^9/6.02214*^23  *1/(Vext*1*^-15)
%Conversion of parameters from nM to molecules: 1.66054/V1 (cell),1.66054/Vext↙
(external)

%% first set Parameter values:

global kd1 kd2 kd3 kd4 kd5 kd6 kd7 kd8 kd9 kd10 kd11 kd12
global k_1 k_2 k_3 k_4 k_5 k_6 k_7 k_8 k_9 k_10 k_11 k_12
global konR WR;
global konA konA_basal WA WA_basal;
global kF chi chiset;
global Kar k_ar;
global dmR;
global dmA;
global PR;
global PA;
global dR dR2;
global dA;
global kC;
global dC;
global D;
global dCR;
global dAR;
global dmRA;

%% second set parameters values

global No N K l v m;
global u umax;
global r;
global Vo Vext Vtot Vc_tot Vx;
global TR TA TA_basal thetaR0 thetaR aR thetaA0 aA aA_basal thetaA thetaA0_basal↙
thetaA_basal kFA kFR;

%%

chiset=1;
F=log(No)+umax.*(t-l+(log(1-exp(-v.*t)+exp(-v.*(t-l)))./v))-(1/m).*log(1+((exp(m.↙
*umax.*(t-l+(log(1-exp(-v.*t)+exp(-v.*(t-l)))./v)))-1)/(exp(m.*(log(K)-log(No))))));
N=exp(F);
u=umax.*((v.*exp(-t.*v) - v.*exp(v.*(l - t)))./(v.*(exp(v.*(l - t)) - exp(-t.*v) + 1))↙
+ 1) - (umax.*exp(m.*umax.*(t - l + log(exp(v.*(l - t)) - exp(-t.*v) + 1)./v))*exp(-m.↙
*(log(K) - log(No))).*((v.*exp(-t.*v) - v.*exp(v.*(l - t)))./(v.*(exp(v*(l - t)) - exp↙
(-t.*v) + 1)) + 1))./(exp(-m.*(log(K) - log(No))).*(exp(m.*umax.*(t - l + log(exp(v.*↙
(l - t)) - exp(-t.*v) + 1)./v)) - 1) + 1);

Vo=1.2;
Vc_tot=N*Vo;
Vtot=1.8e+15;
Vext=Vtot-Vc_tot;
Vx=Vext./Vc_tot;
r=1./Vx;
% kFR=kF*chi;
kFR=kF*chiset;
% kFA=kF/chi;
```

```
kFA=kF/chiset;
thetaR0=konR/(kFR+konR);
WR=kFR*thetaR0;
thetaA0=konA/(kFA+konA);
thetaA0_basal=konA_basal/(kFA+konA_basal);
WA=kFA*thetaA0;
WA_basal=kFA*thetaA0_basal;
aR=thetaR0/(1-thetaR0);
aA=thetaA0/(1-thetaA0);
aA_basal=thetaA0_basal/(1-thetaA0_basal);
thetaR=(aR+1)/(aR+aA+1);
thetaA=(aA+1)/(aR+aA+1);
thetaA_basal=(aA_basal+1)/(aR+aA_basal+1);
TR=kFR*thetaR*thetaR0;
TA=kFA*thetaA*thetaA0;
TA_basal=kFA*thetaA_basal*thetaA0_basal;

% Chemical Reactions and rate laws:
% OR = y(1)
% OA = y(2)
% OR-2R2 = y(3)
% OR-R2 = y(4)
% OA-R2 = y(5)
% OA-AR2 = y(6)
% r = y(7)
% a = y(8)
% r-a = y(9)
% R = y(10)
% R2 = y(11)
% A = y(12)
% C = y(13)
% AR2 = y(14)
% C2-R2 = y(15)
% Ce = y(16)
% OA-2R2 = y(17)
% OA-R2-AR2 = y(18)
% OA-2R2-AR2 = y(19)

%OR + R2 <-> OR-R2
r1 = (k_1/kd1).*y(1).*y(11)-k_1.*y(4);

%OA + R2 <-> OA-R2
r2 = (k_2/kd2).*y(2).*y(11)-k_2.*y(5);

%A + R2 <-> AR2
% r3 = (k_3/kd3).*y(12).*y(11)-k_3.*y(14);
r3=0;

%2C + R2 <-> C2-R2
r4 = (k_4/kd4).*y(11).*(y(13).^2)-k_4.*y(15);
% r4=0;

%OA + AR2 <-> OA-AR2
% r5 = (k_5/kd5).*y(2).*y(14)-k_5.*y(6);
r5=0;

%S (A) -> C
r6 = kC*y(12);

%OR -> OR + r
```

```
r7 = TR.*y(1);
% r7= WR*y(1);

%OR-R2 + R2 <-> OR-2R2
r8 = (k_7/kd7).*y(4).*y(11)-k_7.*y(3);
% r8=0;

%OA -> OA + a (max transcription 1 or basal transcription)
% r9 = TA.*y(2);
% r9 = WA*y(2);
% r9 = 0;
r9 = TA_basal*y(2);
% r9 = WA_basal*y(2);

%OA-AR2 -> OA-AR2 + a (max transcription 2)
% r10 = TA*y(6);
r10=0;
% r10 = WA*y(6);

%OA-2R2 -> OA-2R2 + a (max transcription 2)
r27 = TA*y(17);
% r27=0;

%OA-R2 -> OA-R2 + a (max transcription 2)
r43=TA*y(5);
% r43=0;

%OA-R2-AR2 -> OA-R2-AR2 + a (max transcription 2)
% r44=TA*y(18);
r44=0;

%OA-2R2-AR2 -> OA-2R2-AR2 + a (max transcription 2)
% r45=TA*y(19);
r45=0;

%a + r <-> r-a
% r11 = (k_ar/Kar).*y(7).*y(8)-k_ar.*y(9);
r11=0;

%r -> r + R
r12 = PR.*y(7);

%a -> a + A
r13 = PA.*y(8);

%2R <-> R2
r14 = (k_6/kd6).*(y(10).^2)-k_6.*y(11);

%r ->
r15 = dmR.*y(7)+u.*y(7);

%R ->
r16 = dR.*y(10)+u.*y(10);

%R2 ->
r17 = dR2.*y(11)+u.*y(11);

%a ->
r18 = dmA.*y(8)+u.*y(8);
```

```
%A ->
r19 = dA.*y(12)+u.*y(12);

%C ->
r20 = dC.*y(13)+u.*y(13);

%C2-R2 ->
r21 = dCR.*y(15)+u.*y(15);

%AR2 ->
% r22 = dAR.*y(14)+u.*y(14);
r22=0;

%r-a ->
% r23 = dmRA.*y(9)+u.*y(9);
r23=0;

%Ce ->
r24 = dC.*y(16);

%C -> Ce
r25=D.*(y(16)-y(13));

%Ce -> C
r26=r.*D.*(y(13)-y(16));

%OR-2R2 -> OR-2R2 + OR
r29=u.*y(3);
% r29=0;

%OR-R2 -> OR-R2 + OR
r30=u.*y(4);
% r30=0;

%OA-R2 -> OA-R2 + OA
r31=u.*y(5);
% r31=0;

%OA-AR2 -> OA-AR2 + OA
% r32=u.*y(6);
r32=0;

% OA-R2 + R2 <-> OA-2R2
r33 = (k_8/kd8).*y(5).*y(11)-k_8.*y(17);
% r33=0;

% OA-R2 + AR2 <-> OA-R2-AR2
% r34 = (k_9/kd9).*y(5).*y(14)-k_9.*y(18);
r34=0;

% OA-2R2 + AR2 <-> OA-2R2-AR2
% r35 = (k_10/kd10).*y(17).*y(14)-k_10.*y(19);
r35=0;

% OA-AR2 + R2 <-> OA-R2-AR2
% r36 = (k_11/kd11).*y(6).*y(11)-k_11.*y(18);
r36=0;

% OA-R2-AR2 + R2 <-> OA-2R2-AR2
% r37 = (k_12/kd12).*y(18).*y(11)-k_12.*y(19);
```

```
r37=0;

% OA-2R2 -> OA-2R2 + OA
r38 = u.*y(17);
% r38=0;

% OA-R2-AR2 -> OA-R2-AR2 + OA
% r39 = u.*y(18);
r39=0;

% OA-2R2-AR2 -> OA-2R2-AR2 + OA
% r40 = u.*y(19);
r40=0;

% Correction for Ce: Negative dilution due to the total cells volume growth
r42=r.*u.*y(16);

%Differential equations:
dydt=[r30-r1+r29;... %OR
r31+r32+r38+r39+r40-r2-r5;... % OA
r8-r29;... % OR-2R2
r1-r8-r30;... % OR-R2
r2-r31-r33-r34;... % OA-R2
r5-r32-r36;... % OA-AR2
r7-r11-r15;... % r
r9+r10-r11-r18+r27+r43+r44+r45;... % a
r11-r23;... % r-a
r12-r14-r16;... % R
r14-r1-r2-r3-r4-r8-r17-r33-r36-r37;... % R2
r13-r3-r19;... % A
r6-r4-r20+r25;... % C
r3-r5-r22-r34-r35;... % AR2
r4-r21;... % C2-R2
r26-r24+r42;...  % Ce
r33-r35-r38;... % OA-2R2
r34+r36-r37-r39;... % OA-R2-AR2
r35+r37-r40]; %OA-2R2-AR2

end
```

Scenario E

```
function dydt = GBLE(t, y)

% Units: nM, min, fl (um^3)
%Conversion coefficient molecules per cell -> nM
%nmolesV0 = 1*^9/6.02214*^23  *1/(V0*1*^-15);
%nmolesVext = 1*^9/6.02214*^23  *1/(Vext*1*^-15)
%Conversion of parameters from nM to molecules: 1.66054/V1 (cell),1.66054/Vext↙
(external)

%% first set Parameter values:

global kd1 kd2 kd3 kd4 kd5 kd6 kd7 kd8 kd9 kd10 kd11 kd12
global k_1 k_2 k_3 k_4 k_5 k_6 k_7 k_8 k_9 k_10 k_11 k_12
global konR WR;
global konA konA_basal WA WA_basal;
global kF chi chiset;
global Kar k_ar;
global dmR;
global dmA;
global PR;
global PA;
global dR dR2;
global dA;
global kC;
global dC;
global D;
global dCR;
global dAR;
global dmRA;

%% second set parameters values

global No N K l v m;
global u umax;
global r;
global Vo Vext Vtot Vc_tot Vx;
global TR TA TA_basal thetaR0 thetaR aR thetaA0 aA aA_basal thetaA thetaA0_basal↙
thetaA_basal kFA kFR;

%%

% chiset=1;
F=log(No)+umax.*(t-l+(log(1-exp(-v.*t)+exp(-v.*(t-l)))./v))-(1/m).*log(1+((exp(m.↙
*umax.*(t-l+(log(1-exp(-v.*t)+exp(-v.*(t-l)))./v)))-1)/(exp(m.*(log(K)-log(No))))));
N=exp(F);
u=umax.*((v.*exp(-t.*v) - v.*exp(v.*(l - t)))./(v.*(exp(v.*(l - t)) - exp(-t.*v) + 1))↙
+ 1) - (umax.*exp(m.*umax.*(t - l + log(exp(v.*(l - t)) - exp(-t.*v) + 1)./v))*exp(-m.↙
*(log(K) - log(No))).*((v.*exp(-t.*v) - v.*exp(v.*(l - t)))./(v.*(exp(v*(l - t)) - exp↙
(-t.*v) + 1)) + 1))./(exp(-m.*(log(K) - log(No))).*(exp(m.*umax.*(t - l + log(exp(v.*↙
(l - t)) - exp(-t.*v) + 1)./v)) - 1) + 1);

Vo=1.2;
Vc_tot=N*Vo;
Vtot=1.8e+15;
Vext=Vtot-Vc_tot;
Vx=Vext./Vc_tot;
r=1./Vx;
kFR=kF*chi;
% kFR=kF*chiset;
kFA=kF/chi;
```

```
% kFA=kF/chiset;
thetaR0=konR/(kFR+konR);
WR=kFR*thetaR0;
thetaA0=konA/(kFA+konA);
thetaA0_basal=konA_basal/(kFA+konA_basal);
WA=kFA*thetaA0;
WA_basal=kFA*thetaA0_basal;
aR=thetaR0/(1-thetaR0);
aA=thetaA0/(1-thetaA0);
aA_basal=thetaA0_basal/(1-thetaA0_basal);
thetaR=(aR+1)/(aR+aA+1);
thetaA=(aA+1)/(aR+aA+1);
thetaA_basal=(aA_basal+1)/(aR+aA_basal+1);
TR=kFR*thetaR*thetaR0;
TA=kFA*thetaA*thetaA0;
TA_basal=kFA*thetaA_basal*thetaA0_basal;

% Chemical Reactions and rate laws:
% OR = y(1)
% OA = y(2)
% OR-2R2 = y(3)
% OR-R2 = y(4)
% OA-R2 = y(5)
% OA-AR2 = y(6)
% r = y(7)
% a = y(8)
% r-a = y(9)
% R = y(10)
% R2 = y(11)
% A = y(12)
% C = y(13)
% AR2 = y(14)
% C2-R2 = y(15)
% Ce = y(16)
% OA-2R2 = y(17)
% OA-R2-AR2 = y(18)
% OA-2R2-AR2 = y(19)

%OR + R2 <-> OR-R2
r1 = (k_1/kd1).*y(1).*y(11)-k_1.*y(4);

%OA + R2 <-> OA-R2
r2 = (k_2/kd2).*y(2).*y(11)-k_2.*y(5);

%A + R2 <-> AR2
r3 = (k_3/kd3).*y(12).*y(11)-k_3.*y(14);
% r3=0;

%2C + R2 <-> C2-R2
r4 = (k_4/kd4).*y(11).*(y(13).^2)-k_4.*y(15);
% r4=0;

%OA + AR2 <-> OA-AR2
r5 = (k_5/kd5).*y(2).*y(14)-k_5.*y(6);
% r5=0;

%S (A) -> C
r6 = kC*y(12);

%OR -> OR + r
```

```
r7 = TR.*y(1);
% r7= WR*y(1);

%OR-R2 + R2 <-> OR-2R2
r8 = (k_7/kd7).*y(4).*y(11)-k_7.*y(3);
% r8=0;

%OA -> OA + a (max transcription 1 or basal transcription)
% r9 = TA.*y(2);
% r9 = WA*y(2);
% r9 = 0;
r9 = TA_basal*y(2);
% r9 = WA_basal*y(2);

%OA-AR2 -> OA-AR2 + a (max transcription 2)
r10 = TA*y(6);
% r10=0;
% r10 = WA*y(6);

%a + r <-> r-a
r11 = (k_ar/Kar).*y(7).*y(8)-k_ar.*y(9);
% r11=0;

%r -> r + R
r12 = PR.*y(7);

%a -> a + A
r13 = PA.*y(8);

%2R <-> R2
r14 = (k_6/kd6).*(y(10).^2)-k_6.*y(11);

%r ->
r15 = dmR.*y(7)+u.*y(7);

%R ->
r16 = dR.*y(10)+u.*y(10);

%R2 ->
r17 = dR2.*y(11)+u.*y(11);

%a ->
r18 = dmA.*y(8)+u.*y(8);

%A ->
r19 = dA.*y(12)+u.*y(12);

%C ->
r20 = dC.*y(13)+u.*y(13);

%C2-R2 ->
r21 = dCR.*y(15)+u.*y(15);

%AR2 ->
r22 = dAR.*y(14)+u.*y(14);
% r22=0;

%r-a ->
r23 = dmRA.*y(9)+u.*y(9);
% r23=0;
```

```
%Ce ->
r24 = dC.*y(16);

%C -> Ce
r25=D.*(y(16)-y(13));

%Ce -> C
r26=r.*D.*(y(13)-y(16));

%OR-2R2 -> OR-2R2 + OR
r29=u.*y(3);
% r29=0;

%OR-R2 -> OR-R2 + OR
r30=u.*y(4);
% r30=0;

%OA-R2 -> OA-R2 + OA
r31=u.*y(5);
% r31=0;

%OA-AR2 -> OA-AR2 + OA
r32=u.*y(6);
% r32=0;

% OA-R2 + R2 <-> OA-2R2
r33 = (k_8/kd8).*y(5).*y(11)-k_8.*y(17);
% r33=0;

% OA-R2 + AR2 <-> OA-R2-AR2
r34 = (k_9/kd9).*y(5).*y(14)-k_9.*y(18);
% r34=0;

% OA-2R2 + AR2 <-> OA-2R2-AR2
r35 = (k_10/kd10).*y(17).*y(14)-k_10.*y(19);
% r35=0;

% OA-AR2 + R2 <-> OA-R2-AR2
r36 = (k_11/kd11).*y(6).*y(11)-k_11.*y(18);
% r36=0;

% OA-R2-AR2 + R2 <-> OA-2R2-AR2
r37 = (k_12/kd12).*y(18).*y(11)-k_12.*y(19);
% r37=0;

% OA-2R2 -> OA-2R2 + OA
r38 = u.*y(17);
% r38=0;

% OA-R2-AR2 -> OA-R2-AR2 + OA
r39 = u.*y(18);
% r39=0;

% OA-2R2-AR2 -> OA-2R2-AR2 + OA
r40 = u.*y(19);
% r40=0;

% Correction for Ce: Negative dilution due to the total cells volume growth
r42=r.*u.*y(16);
```

```
%Differential equations:
dydt=[r30-r1+r29;... %OR
r31+r32+r38+r39+r40-r2-r5;... % OA
r8-r29;... % OR-2R2
r1-r8-r30;... % OR-R2
r2-r31-r33-r34;... % OA-R2
r5-r32-r36;... % OA-AR2
r7-r11-r15;... % r
r9+r10-r11-r18;... % a
r11-r23;... % r-a
r12-r14-r16;... % R
r14-r1-r2-r3-r4-r8-r17-r33-r36-r37;... % R2
r13-r3-r19;... % A
r6-r4-r20+r25;... % C
r3-r5-r22-r34-r35;... % AR2
r4-r21;... % C2-R2
r26-r24+r42;...  % Ce
r33-r35-r38;... % OA-2R2
r34+r36-r37-r39;... % OA-R2-AR2
r35+r37-r40]; %OA-2R2-AR2

end
```

Scenario F

```
function dydt = GBLF(t, y)

% Units: nM, min, fl (um^3)
%Conversion coefficient molecules per cell -> nM
%nmolesV0 = 1*^9/6.02214*^23  *1/(V0*1*^-15);
%nmolesVext = 1*^9/6.02214*^23  *1/(Vext*1*^-15)
%Conversion of parameters from nM to molecules: 1.66054/V1 (cell),1.66054/Vext↙
(external)

%% first set Parameter values:

global kd1 kd2 kd3 kd4 kd5 kd6 kd7 kd8 kd9 kd10 kd11 kd12
global k_1 k_2 k_3 k_4 k_5 k_6 k_7 k_8 k_9 k_10 k_11 k_12
global konR WR;
global konA konA_basal WA WA_basal;
global kF chi chiset;
global Kar k_ar;
global dmR;
global dmA;
global PR;
global PA;
global dR dR2;
global dA;
global kC;
global dC;
global D;
global dCR;
global dAR;
global dmRA;

%% second set parameters values

global No N K l v m;
global u umax;
global r;
global Vo Vext Vtot Vc_tot Vx;
global TR TA TA_basal thetaR0 thetaR aR thetaA0 aA aA_basal thetaA thetaA0_basal↙
thetaA_basal kFA kFR;

%%

% chiset=1;
F=log(No)+umax.*(t-l+(log(1-exp(-v.*t)+exp(-v.*(t-l)))./v))-(1/m).*log(1+((exp(m.↙
*umax.*(t-l+(log(1-exp(-v.*t)+exp(-v.*(t-l)))./v)))-1)/(exp(m.*(log(K)-log(No))))));
N=exp(F);
u=umax.*((v.*exp(-t.*v) - v.*exp(v.*(l - t)))./(v.*(exp(v.*(l - t)) - exp(-t.*v) + 1))↙
+ 1) - (umax.*exp(m.*umax.*(t - l + log(exp(v.*(l - t)) - exp(-t.*v) + 1)./v))*exp(-m.↙
*(log(K) - log(No))).*((v.*exp(-t.*v) - v.*exp(v.*(l - t)))./(v.*(exp(v*(l - t)) - exp↙
(-t.*v) + 1)) + 1))./(exp(-m.*(log(K) - log(No))).*(exp(m.*umax.*(t - l + log(exp(v.*↙
(l - t)) - exp(-t.*v) + 1)./v)) - 1) + 1);

Vo=1.2;
Vc_tot=N*Vo;
Vtot=1.8e+15;
Vext=Vtot-Vc_tot;
Vx=Vext./Vc_tot;
r=1./Vx;
kFR=kF*chi;
% kFR=kF*chiset;
kFA=kF/chi;
```

```
% kFA=kF/chiset;
thetaR0=konR/(kFR+konR);
WR=kFR*thetaR0;
thetaA0=konA/(kFA+konA);
thetaA0_basal=konA_basal/(kFA+konA_basal);
WA=kFA*thetaA0;
WA_basal=kFA*thetaA0_basal;
aR=thetaR0/(1-thetaR0);
aA=thetaA0/(1-thetaA0);
aA_basal=thetaA0_basal/(1-thetaA0_basal);
thetaR=(aR+1)/(aR+aA+1);
thetaA=(aA+1)/(aR+aA+1);
thetaA_basal=(aA_basal+1)/(aR+aA_basal+1);
TR=kFR*thetaR*thetaR0;
TA=kFA*thetaA*thetaA0;
TA_basal=kFA*thetaA_basal*thetaA0_basal;

% Chemical Reactions and rate laws:
% OR = y(1)
% OA = y(2)
% OR-2R2 = y(3)
% OR-R2 = y(4)
% OA-R2 = y(5)
% OA-AR2 = y(6)
% r = y(7)
% a = y(8)
% r-a = y(9)
% R = y(10)
% R2 = y(11)
% A = y(12)
% C = y(13)
% AR2 = y(14)
% C2-R2 = y(15)
% Ce = y(16)
% OA-2R2 = y(17)
% OA-R2-AR2 = y(18)
% OA-2R2-AR2 = y(19)

%OR + R2 <-> OR-R2
r1 = (k_1/kd1).*y(1).*y(11)-k_1.*y(4);

%OA + R2 <-> OA-R2
r2 = (k_2/kd2).*y(2).*y(11)-k_2.*y(5);

%A + R2 <-> AR2
r3 = (k_3/kd3).*y(12).*y(11)-k_3.*y(14);
% r3=0;

%2C + R2 <-> C2-R2
r4 = (k_4/kd4).*y(11).*(y(13).^2)-k_4.*y(15);
% r4=0;

%OA + AR2 <-> OA-AR2
r5 = (k_5/kd5).*y(2).*y(14)-k_5.*y(6);
% r5=0;

%S (A) -> C
r6 = kC*y(12);

%OR -> OR + r
```

```
r7 = TR.*y(1);
% r7= WR*y(1);

%OR-R2 + R2 <-> OR-2R2
r8 = (k_7/kd7).*y(4).*y(11)-k_7.*y(3);
% r8=0;

%OA -> OA + a (max transcription 1 or basal transcription)
% r9 = TA.*y(2);
% r9 = WA*y(2);
% r9 = 0;
r9 = TA_basal*y(2);
% r9 = WA_basal*y(2);

%OA-AR2 -> OA-AR2 + a (max transcription 2)
r10 = TA*y(6);
% r10=0;
% r10 = WA*y(6);

%OA-2R2 -> OA-2R2 + a (max transcription 2)
r27 = TA*y(17);
% r27=0;

%OA-R2 -> OA-R2 + a (max transcription 2)
r43=TA*y(5);
% r43=0;

%OA-R2-AR2 -> OA-R2-AR2 + a (max transcription 2)
r44=TA*y(18);
% r44=0;

%OA-2R2-AR2 -> OA-2R2-AR2 + a (max transcription 2)
r45=TA*y(19);
% r45=0;

%a + r <-> r-a
% r11 = (k_ar/Kar).*y(7).*y(8)-k_ar.*y(9);
r11=0;

%r -> r + R
r12 = PR.*y(7);

%a -> a + A
r13 = PA.*y(8);

%2R <-> R2
r14 = (k_6/kd6).*(y(10).^2)-k_6.*y(11);

%r ->
r15 = dmR.*y(7)+u.*y(7);

%R ->
r16 = dR.*y(10)+u.*y(10);

%R2 ->
r17 = dR2.*y(11)+u.*y(11);

%a ->
r18 = dmA.*y(8)+u.*y(8);
```

```
%A ->
r19 = dA.*y(12)+u.*y(12);

%C ->
r20 = dC.*y(13)+u.*y(13);

%C2-R2 ->
r21 = dCR.*y(15)+u.*y(15);

%AR2 ->
r22 = dAR.*y(14)+u.*y(14);
% r22=0;

%r-a ->
% r23 = dmRA.*y(9)+u.*y(9);
r23=0;

%Ce ->
r24 = dC.*y(16);

%C -> Ce
r25=D.*(y(16)-y(13));

%Ce -> C
r26=r.*D.*(y(13)-y(16));

%OR-2R2 -> OR-2R2 + OR
r29=u.*y(3);
% r29=0;

%OR-R2 -> OR-R2 + OR
r30=u.*y(4);
% r30=0;

%OA-R2 -> OA-R2 + OA
r31=u.*y(5);
% r31=0;

%OA-AR2 -> OA-AR2 + OA
r32=u.*y(6);
% r32=0;

% OA-R2 + R2 <-> OA-2R2
r33 = (k_8/kd8).*y(5).*y(11)-k_8.*y(17);
% r33=0;

% OA-R2 + AR2 <-> OA-R2-AR2
r34 = (k_9/kd9).*y(5).*y(14)-k_9.*y(18);
% r34=0;

% OA-2R2 + AR2 <-> OA-2R2-AR2
r35 = (k_10/kd10).*y(17).*y(14)-k_10.*y(19);
% r35=0;

% OA-AR2 + R2 <-> OA-R2-AR2
r36 = (k_11/kd11).*y(6).*y(11)-k_11.*y(18);
% r36=0;

% OA-R2-AR2 + R2 <-> OA-2R2-AR2
r37 = (k_12/kd12).*y(18).*y(11)-k_12.*y(19);
```

```
% r37=0;

% OA-2R2 -> OA-2R2 + OA
r38 = u.*y(17);
% r38=0;

% OA-R2-AR2 -> OA-R2-AR2 + OA
r39 = u.*y(18);
% r39=0;

% OA-2R2-AR2 -> OA-2R2-AR2 + OA
r40 = u.*y(19);
% r40=0;

% Correction for Ce: Negative dilution due to the total cells volume growth
r42=r.*u.*y(16);

%Differential equations:
dydt=[r30-r1+r29;... %OR
r31+r32+r38+r39+r40-r2-r5;... % OA
r8-r29;... % OR-2R2
r1-r8-r30;... % OR-R2
r2-r31-r33-r34;... % OA-R2
r5-r32-r36;... % OA-AR2
r7-r11-r15;... % r
r9+r10-r11-r18+r27+r43+r44+r45;... % a
r11-r23;... % r-a
r12-r14-r16;... % R
r14-r1-r2-r3-r4-r8-r17-r33-r36-r37;... % R2
r13-r3-r19;... % A
r6-r4-r20+r25;... % C
r3-r5-r22-r34-r35;... % AR2
r4-r21;... % C2-R2
r26-r24+r42;...  % Ce
r33-r35-r38;... % OA-2R2
r34+r36-r37-r39;... % OA-R2-AR2
r35+r37-r40]; %OA-2R2-AR2

end
```

Scenario G

```
function dydt = GBLG(t, y)

% Units: nM, min, fl (um^3)
%Conversion coefficient molecules per cell -> nM
%nmolesV0 = 1*^9/6.02214*^23  *1/(V0*1*^-15);
%nmolesVext = 1*^9/6.02214*^23  *1/(Vext*1*^-15)
%Conversion of parameters from nM to molecules: 1.66054/V1 (cell),1.66054/Vext↙
(external)

%% first set Parameter values:

global kd1 kd2 kd3 kd4 kd5 kd6 kd7 kd8 kd9 kd10 kd11 kd12
global k_1 k_2 k_3 k_4 k_5 k_6 k_7 k_8 k_9 k_10 k_11 k_12
global konR WR;
global konA konA_basal WA WA_basal;
global kF chi chiset;
global Kar k_ar;
global dmR;
global dmA;
global PR;
global PA;
global dR dR2;
global dA;
global kC;
global dC;
global D;
global dCR;
global dAR;
global dmRA;

%% second set parameters values

global No N K l v m;
global u umax;
global r;
global Vo Vext Vtot Vc_tot Vx;
global TR TA TA_basal thetaR0 thetaR aR thetaA0 aA aA_basal thetaA thetaA0_basal↙
thetaA_basal kFA kFR;

%%

% chiset=1;
F=log(No)+umax.*(t-l+(log(1-exp(-v.*t)+exp(-v.*(t-l)))./v))-(1/m).*log(1+((exp(m.↙
*umax.*(t-l+(log(1-exp(-v.*t)+exp(-v.*(t-l)))./v)))-1)/(exp(m.*(log(K)-log(No))))));
N=exp(F);
u=umax.*((v.*exp(-t.*v) - v.*exp(v.*(l - t)))./(v.*(exp(v.*(l - t)) - exp(-t.*v) + 1))↙
+ 1) - (umax.*exp(m.*umax.*(t - l + log(exp(v.*(l - t)) - exp(-t.*v) + 1)./v))*exp(-m.↙
*(log(K) - log(No))).*((v.*exp(-t.*v) - v.*exp(v.*(l - t)))./(v.*(exp(v*(l - t)) - exp↙
(-t.*v) + 1)) + 1))./(exp(-m.*(log(K) - log(No))).*(exp(m.*umax.*(t - l + log(exp(v.*↙
(l - t)) - exp(-t.*v) + 1)./v)) - 1) + 1);

Vo=1.2;
Vc_tot=N*Vo;
Vtot=1.8e+15;
Vext=Vtot-Vc_tot;
Vx=Vext./Vc_tot;
r=1./Vx;
kFR=kF*chi;
% kFR=kF*chiset;
kFA=kF/chi;
```

```
% kFA=kF/chiset;
thetaR0=konR/(kFR+konR);
WR=kFR*thetaR0;
thetaA0=konA/(kFA+konA);
thetaA0_basal=konA_basal/(kFA+konA_basal);
WA=kFA*thetaA0;
WA_basal=kFA*thetaA0_basal;
aR=thetaR0/(1-thetaR0);
aA=thetaA0/(1-thetaA0);
aA_basal=thetaA0_basal/(1-thetaA0_basal);
thetaR=(aR+1)/(aR+aA+1);
thetaA=(aA+1)/(aR+aA+1);
thetaA_basal=(aA_basal+1)/(aR+aA_basal+1);
TR=kFR*thetaR*thetaR0;
TA=kFA*thetaA*thetaA0;
TA_basal=kFA*thetaA_basal*thetaA0_basal;

% Chemical Reactions and rate laws:
% OR = y(1)
% OA = y(2)
% OR-2R2 = y(3)
% OR-R2 = y(4)
% OA-R2 = y(5)
% OA-AR2 = y(6)
% r = y(7)
% a = y(8)
% r-a = y(9)
% R = y(10)
% R2 = y(11)
% A = y(12)
% C = y(13)
% AR2 = y(14)
% C2-R2 = y(15)
% Ce = y(16)
% OA-2R2 = y(17)
% OA-R2-AR2 = y(18)
% OA-2R2-AR2 = y(19)

%OR + R2 <-> OR-R2
r1 = (k_1/kd1).*y(1).*y(11)-k_1.*y(4);

%OA + R2 <-> OA-R2
r2 = (k_2/kd2).*y(2).*y(11)-k_2.*y(5);

%A + R2 <-> AR2
% r3 = (k_3/kd3).*y(12).*y(11)-k_3.*y(14);
r3=0;

%2C + R2 <-> C2-R2
r4 = (k_4/kd4).*y(11).*(y(13).^2)-k_4.*y(15);
% r4=0;

%OA + AR2 <-> OA-AR2
% r5 = (k_5/kd5).*y(2).*y(14)-k_5.*y(6);
r5=0;

%S (A) -> C
r6 = kC*y(12);

%OR -> OR + r
```

```
r7 = TR.*y(1);
% r7= WR*y(1);

%OR-R2 + R2 <-> OR-2R2
r8 = (k_7/kd7).*y(4).*y(11)-k_7.*y(3);
% r8=0;

%OA -> OA + a (max transcription 1 or basal transcription)
% r9 = TA.*y(2);
% r9 = WA*y(2);
% r9 = 0;
r9 = TA_basal*y(2);
% r9 = WA_basal*y(2);

%OA-AR2 -> OA-AR2 + a (max transcription 2)
% r10 = TA*y(6);
r10=0;
% r10 = WA*y(6);

%OA-2R2 -> OA-2R2 + a (max transcription 2)
r27 = TA*y(17);
% r27=0;

%OA-R2 -> OA-R2 + a (max transcription 2)
r43=TA*y(5);
% r43=0;

%OA-R2-AR2 -> OA-R2-AR2 + a (max transcription 2)
% r44=TA*y(18);
r44=0;

%OA-2R2-AR2 -> OA-2R2-AR2 + a (max transcription 2)
% r45=TA*y(19);
r45=0;

%a + r <-> r-a
r11 = (k_ar/Kar).*y(7).*y(8)-k_ar.*y(9);
% r11=0;

%r -> r + R
r12 = PR.*y(7);

%a -> a + A
r13 = PA.*y(8);

%2R <-> R2
r14 = (k_6/kd6).*(y(10).^2)-k_6.*y(11);

%r ->
r15 = dmR.*y(7)+u.*y(7);

%R ->
r16 = dR.*y(10)+u.*y(10);

%R2 ->
r17 = dR2.*y(11)+u.*y(11);

%a ->
r18 = dmA.*y(8)+u.*y(8);
```

```
%A ->
r19 = dA.*y(12)+u.*y(12);

%C ->
r20 = dC.*y(13)+u.*y(13);

%C2-R2 ->
r21 = dCR.*y(15)+u.*y(15);

%AR2 ->
% r22 = dAR.*y(14)+u.*y(14);
r22=0;

%r-a ->
r23 = dmRA.*y(9)+u.*y(9);
% r23=0;

%Ce ->
r24 = dC.*y(16);

%C -> Ce
r25=D.*(y(16)-y(13));

%Ce -> C
r26=r.*D.*(y(13)-y(16));

%OR-2R2 -> OR-2R2 + OR
r29=u.*y(3);
% r29=0;

%OR-R2 -> OR-R2 + OR
r30=u.*y(4);
% r30=0;

%OA-R2 -> OA-R2 + OA
r31=u.*y(5);
% r31=0;

%OA-AR2 -> OA-AR2 + OA
% r32=u.*y(6);
r32=0;

% OA-R2 + R2 <-> OA-2R2
r33 = (k_8/kd8).*y(5).*y(11)-k_8.*y(17);
% r33=0;

% OA-R2 + AR2 <-> OA-R2-AR2
% r34 = (k_9/kd9).*y(5).*y(14)-k_9.*y(18);
r34=0;

% OA-2R2 + AR2 <-> OA-2R2-AR2
% r35 = (k_10/kd10).*y(17).*y(14)-k_10.*y(19);
r35=0;

% OA-AR2 + R2 <-> OA-R2-AR2
% r36 = (k_11/kd11).*y(6).*y(11)-k_11.*y(18);
r36=0;

% OA-R2-AR2 + R2 <-> OA-2R2-AR2
% r37 = (k_12/kd12).*y(18).*y(11)-k_12.*y(19);
```

```
r37=0;

% OA-2R2 -> OA-2R2 + OA
r38 = u.*y(17);
% r38=0;

% OA-R2-AR2 -> OA-R2-AR2 + OA
% r39 = u.*y(18);
r39=0;

% OA-2R2-AR2 -> OA-2R2-AR2 + OA
% r40 = u.*y(19);
r40=0;

% Correction for Ce: Negative dilution due to the total cells volume growth
r42=r.*u.*y(16);

%Differential equations:
dydt=[r30-r1+r29;... %OR
r31+r32+r38+r39+r40-r2-r5;... % OA
r8-r29;... % OR-2R2
r1-r8-r30;... % OR-R2
r2-r31-r33-r34;... % OA-R2
r5-r32-r36;... % OA-AR2
r7-r11-r15;... % r
r9+r10-r11-r18+r27+r43+r44+r45;... % a
r11-r23;... % r-a
r12-r14-r16;... % R
r14-r1-r2-r3-r4-r8-r17-r33-r36-r37;... % R2
r13-r3-r19;... % A
r6-r4-r20+r25;... % C
r3-r5-r22-r34-r35;... % AR2
r4-r21;... % C2-R2
r26-r24+r42;...  % Ce
r33-r35-r38;... % OA-2R2
r34+r36-r37-r39;... % OA-R2-AR2
r35+r37-r40]; %OA-2R2-AR2

end
```

Scenario H

```
function dydt = GBLH(t, y)

% Units: nM, min, fl (um^3)
%Conversion coefficient molecules per cell -> nM
%nmolesV0 = 1*^9/6.02214*^23  *1/(V0*1*^-15);
%nmolesVext = 1*^9/6.02214*^23  *1/(Vext*1*^-15)
%Conversion of parameters from nM to molecules: 1.66054/V1 (cell),1.66054/Vext↙
(external)

%% first set Parameter values:

global kd1 kd2 kd3 kd4 kd5 kd6 kd7 kd8 kd9 kd10 kd11 kd12
global k_1 k_2 k_3 k_4 k_5 k_6 k_7 k_8 k_9 k_10 k_11 k_12
global konR WR;
global konA konA_basal WA WA_basal;
global kF chi chiset;
global Kar k_ar;
global dmR;
global dmA;
global PR;
global PA;
global dR dR2;
global dA;
global kC;
global dC;
global D;
global dCR;
global dAR;
global dmRA;

%% second set parameters values

global No N K l v m;
global u umax;
global r;
global Vo Vext Vtot Vc_tot Vx;
global TR TA TA_basal thetaR0 thetaR aR thetaA0 aA aA_basal thetaA thetaA0_basal↙
thetaA_basal kFA kFR;

%%

% chiset=1;
F=log(No)+umax.*(t-l+(log(1-exp(-v.*t)+exp(-v.*(t-l)))./v))-(1/m).*log(1+((exp(m.↙
*umax.*(t-l+(log(1-exp(-v.*t)+exp(-v.*(t-l)))./v)))-1)/(exp(m.*(log(K)-log(No))))));
N=exp(F);
u=umax.*((v.*exp(-t.*v) - v.*exp(v.*(l - t)))./(v.*(exp(v.*(l - t)) - exp(-t.*v) + 1))↙
+ 1) - (umax.*exp(m.*umax.*(t - l + log(exp(v.*(l - t)) - exp(-t.*v) + 1)./v))*exp(-m.↙
*(log(K) - log(No))).*((v.*exp(-t.*v) - v.*exp(v.*(l - t)))./(v.*(exp(v*(l - t)) - exp↙
(-t.*v) + 1)) + 1))./(exp(-m.*(log(K) - log(No))).*(exp(m.*umax.*(t - l + log(exp(v.*↙
(l - t)) - exp(-t.*v) + 1)./v)) - 1) + 1);

Vo=1.2;
Vc_tot=N*Vo;
Vtot=1.8e+15;
Vext=Vtot-Vc_tot;
Vx=Vext./Vc_tot;
r=1./Vx;
kFR=kF*chi;
% kFR=kF*chiset;
kFA=kF/chi;
```

```
% kFA=kF/chiset;
thetaR0=konR/(kFR+konR);
WR=kFR*thetaR0;
thetaA0=konA/(kFA+konA);
thetaA0_basal=konA_basal/(kFA+konA_basal);
WA=kFA*thetaA0;
WA_basal=kFA*thetaA0_basal;
aR=thetaR0/(1-thetaR0);
aA=thetaA0/(1-thetaA0);
aA_basal=thetaA0_basal/(1-thetaA0_basal);
thetaR=(aR+1)/(aR+aA+1);
thetaA=(aA+1)/(aR+aA+1);
thetaA_basal=(aA_basal+1)/(aR+aA_basal+1);
TR=kFR*thetaR*thetaR0;
TA=kFA*thetaA*thetaA0;
TA_basal=kFA*thetaA_basal*thetaA0_basal;

% Chemical Reactions and rate laws:
% OR = y(1)
% OA = y(2)
% OR-2R2 = y(3)
% OR-R2 = y(4)
% OA-R2 = y(5)
% OA-AR2 = y(6)
% r = y(7)
% a = y(8)
% r-a = y(9)
% R = y(10)
% R2 = y(11)
% A = y(12)
% C = y(13)
% AR2 = y(14)
% C2-R2 = y(15)
% Ce = y(16)
% OA-2R2 = y(17)
% OA-R2-AR2 = y(18)
% OA-2R2-AR2 = y(19)

%OR + R2 <-> OR-R2
r1 = (k_1/kd1).*y(1).*y(11)-k_1.*y(4);

%OA + R2 <-> OA-R2
r2 = (k_2/kd2).*y(2).*y(11)-k_2.*y(5);

%A + R2 <-> AR2
r3 = (k_3/kd3).*y(12).*y(11)-k_3.*y(14);
% r3=0;

%2C + R2 <-> C2-R2
r4 = (k_4/kd4).*y(11).*(y(13).^2)-k_4.*y(15);
% r4=0;

%OA + AR2 <-> OA-AR2
r5 = (k_5/kd5).*y(2).*y(14)-k_5.*y(6);
% r5=0;

%S (A) -> C
r6 = kC*y(12);

%OR -> OR + r
```

```
r7 = TR.*y(1);
% r7= WR*y(1);

%OR-R2 + R2 <-> OR-2R2
r8 = (k_7/kd7).*y(4).*y(11)-k_7.*y(3);
% r8=0;

%OA -> OA + a (max transcription 1 or basal transcription)
% r9 = TA.*y(2);
% r9 = WA*y(2);
% r9 = 0;
r9 = TA_basal*y(2);
% r9 = WA_basal*y(2);

%OA-AR2 -> OA-AR2 + a (max transcription 2)
r10 = TA*y(6);
% r10=0;
% r10 = WA*y(6);

%OA-2R2 -> OA-2R2 + a (max transcription 2)
r27 = TA*y(17);
% r27=0;

%OA-R2 -> OA-R2 + a (max transcription 2)
r43=TA*y(5);
% r43=0;

%OA-R2-AR2 -> OA-R2-AR2 + a (max transcription 2)
r44=TA*y(18);
% r44=0;

%OA-2R2-AR2 -> OA-2R2-AR2 + a (max transcription 2)
r45=TA*y(19);
% r45=0;

%a + r <-> r-a
r11 = (k_ar/Kar).*y(7).*y(8)-k_ar.*y(9);
% r11=0;

%r -> r + R
r12 = PR.*y(7);

%a -> a + A
r13 = PA.*y(8);

%2R <-> R2
r14 = (k_6/kd6).*(y(10).^2)-k_6.*y(11);

%r ->
r15 = dmR.*y(7)+u.*y(7);

%R ->
r16 = dR.*y(10)+u.*y(10);

%R2 ->
r17 = dR2.*y(11)+u.*y(11);

%a ->
r18 = dmA.*y(8)+u.*y(8);
```

```
%A ->
r19 = dA.*y(12)+u.*y(12);

%C ->
r20 = dC.*y(13)+u.*y(13);

%C2-R2 ->
r21 = dCR.*y(15)+u.*y(15);

%AR2 ->
r22 = dAR.*y(14)+u.*y(14);
% r22=0;

%r-a ->
r23 = dmRA.*y(9)+u.*y(9);
% r23=0;

%Ce ->
r24 = dC.*y(16);

%C -> Ce
r25=D.*(y(16)-y(13));

%Ce -> C
r26=r.*D.*(y(13)-y(16));

%OR-2R2 -> OR-2R2 + OR
r29=u.*y(3);
% r29=0;

%OR-R2 -> OR-R2 + OR
r30=u.*y(4);
% r30=0;

%OA-R2 -> OA-R2 + OA
r31=u.*y(5);
% r31=0;

%OA-AR2 -> OA-AR2 + OA
r32=u.*y(6);
% r32=0;

% OA-R2 + R2 <-> OA-2R2
r33 = (k_8/kd8).*y(5).*y(11)-k_8.*y(17);
% r33=0;

% OA-R2 + AR2 <-> OA-R2-AR2
r34 = (k_9/kd9).*y(5).*y(14)-k_9.*y(18);
% r34=0;

% OA-2R2 + AR2 <-> OA-2R2-AR2
r35 = (k_10/kd10).*y(17).*y(14)-k_10.*y(19);
% r35=0;

% OA-AR2 + R2 <-> OA-R2-AR2
r36 = (k_11/kd11).*y(6).*y(11)-k_11.*y(18);
% r36=0;

% OA-R2-AR2 + R2 <-> OA-2R2-AR2
r37 = (k_12/kd12).*y(18).*y(11)-k_12.*y(19);
```

```
% r37=0;

% OA-2R2 -> OA-2R2 + OA
r38 = u.*y(17);
% r38=0;

% OA-R2-AR2 -> OA-R2-AR2 + OA
r39 = u.*y(18);
% r39=0;

% OA-2R2-AR2 -> OA-2R2-AR2 + OA
r40 = u.*y(19);
% r40=0;

% Correction for Ce: Negative dilution due to the total cells volume growth
r42=r.*u.*y(16);

%Differential equations:
dydt=[r30-r1+r29;... %OR
r31+r32+r38+r39+r40-r2-r5;... % OA
r8-r29;... % OR-2R2
r1-r8-r30;... % OR-R2
r2-r31-r33-r34;... % OA-R2
r5-r32-r36;... % OA-AR2
r7-r11-r15;... % r
r9+r10-r11-r18+r27+r43+r44+r45;... % a
r11-r23;... % r-a
r12-r14-r16;... % R
r14-r1-r2-r3-r4-r8-r17-r33-r36-r37;... % R2
r13-r3-r19;... % A
r6-r4-r20+r25;... % C
r3-r5-r22-r34-r35;... % AR2
r4-r21;... % C2-R2
r26-r24+r42;...  % Ce
r33-r35-r38;... % OA-2R2
r34+r36-r37-r39;... % OA-R2-AR2
r35+r37-r40]; %OA-2R2-AR2

end
```
