# Supplementary material for: Unravelling the γ-butyrolactone network in Streptomyces coelicolor by computational ensemble modelling
Source: PLoS Comput Biol. 2020 Jul 10;16(7):e1008039. doi: 10.1371/journal.pcbi.1008039 (PMC7384680; doi:10.1371/journal.pcbi.1008039)
Supplement: S4 Appendix — (PDF) [file pcbi.1008039.s004.pdf]

S4 Appendix - Supplementary Figures

Simulation Scenarios

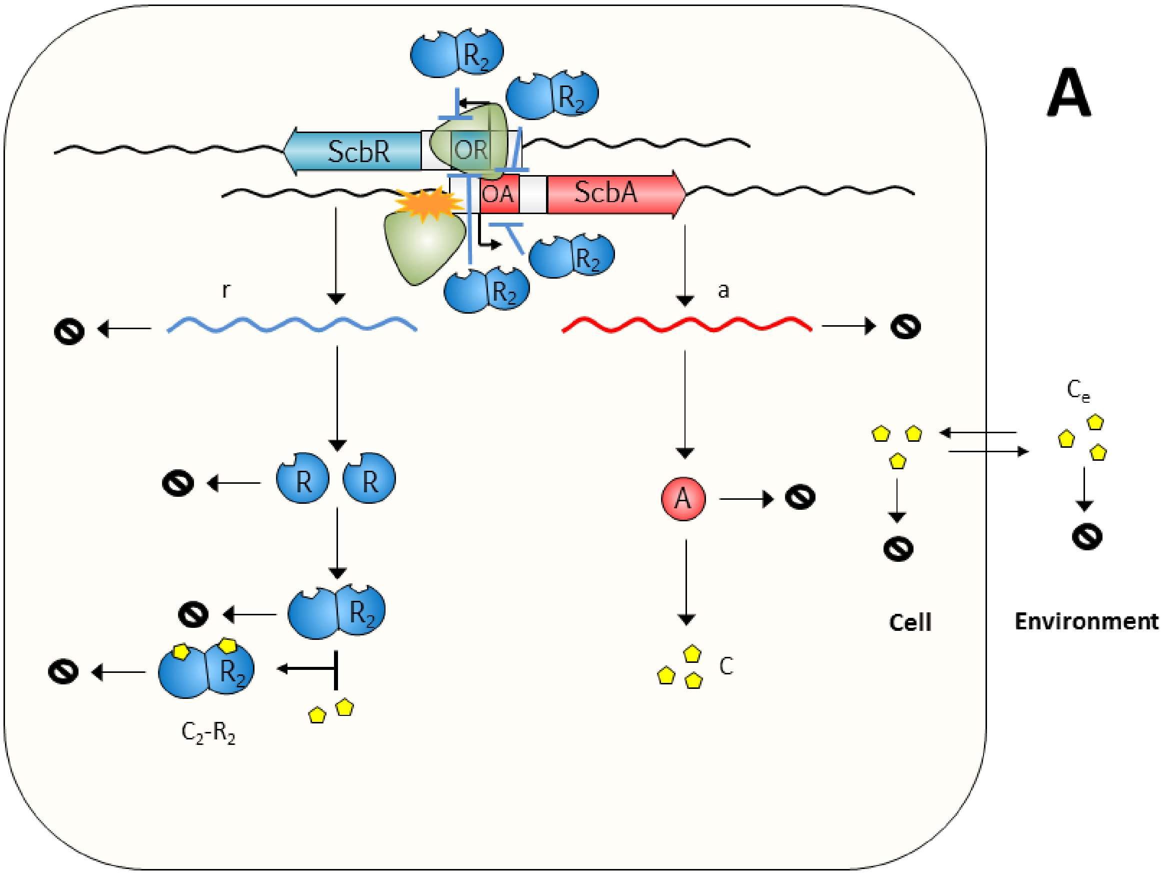

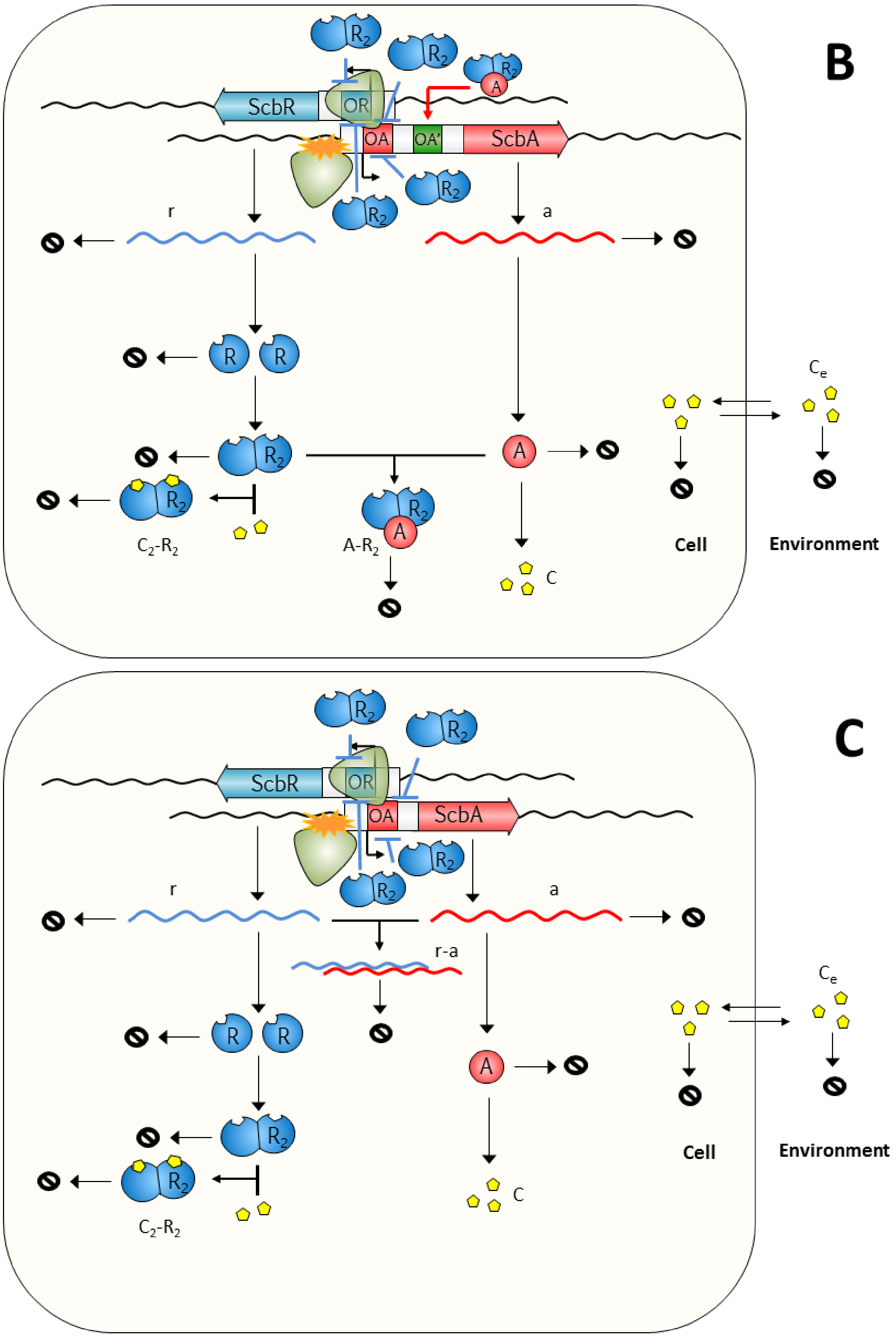
B
ScbR
OR
OA
OA'
ScbA
R2
A
r
a
R
A-R2
C2-R2
C
Ce
Cell
Environment
C
ScbR
OR
OA
ScbA
R2
r
a
r-a
R
A
C2-R2
C
Ce
Cell
Environment

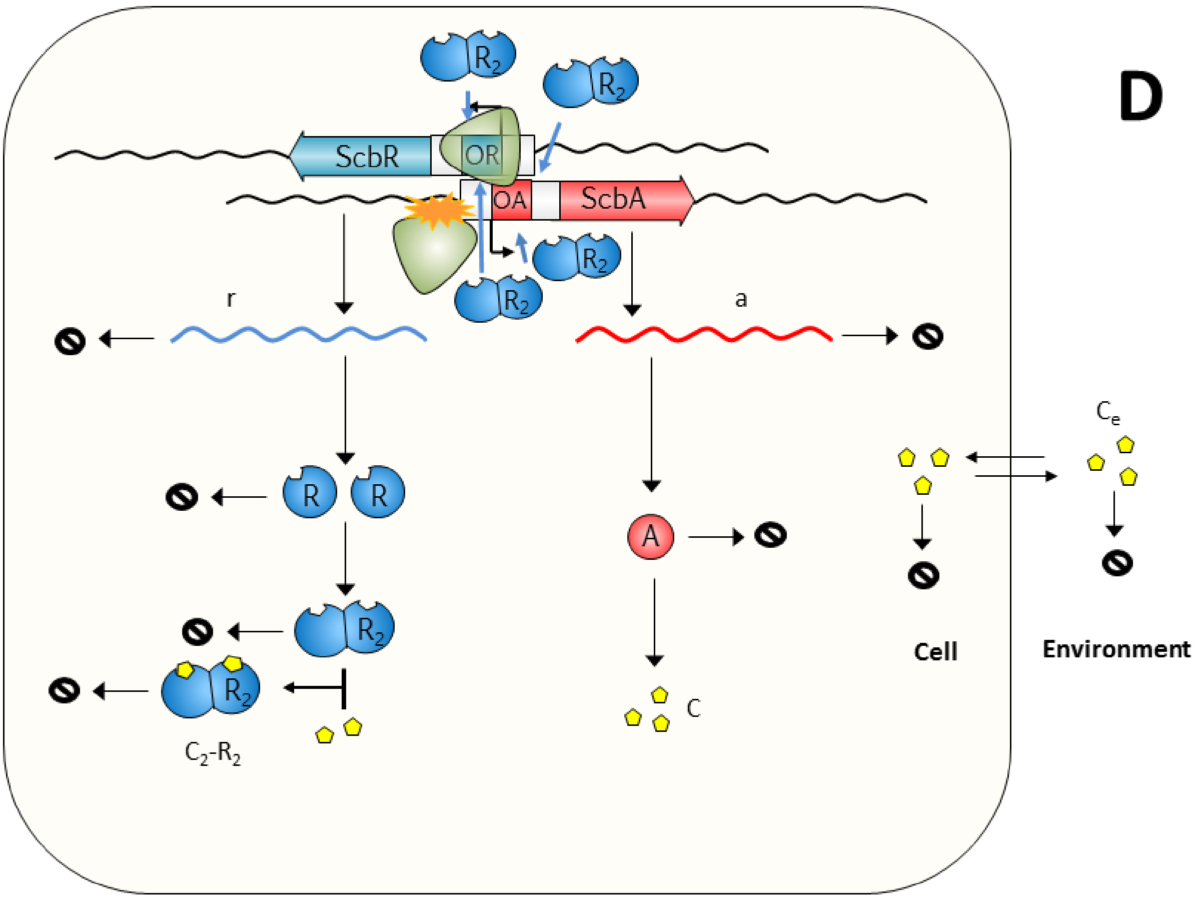
D
ScbR
OR
OA
ScbA
R2
r
a
R
A
C
Ce
C2-R2
Cell
Environment

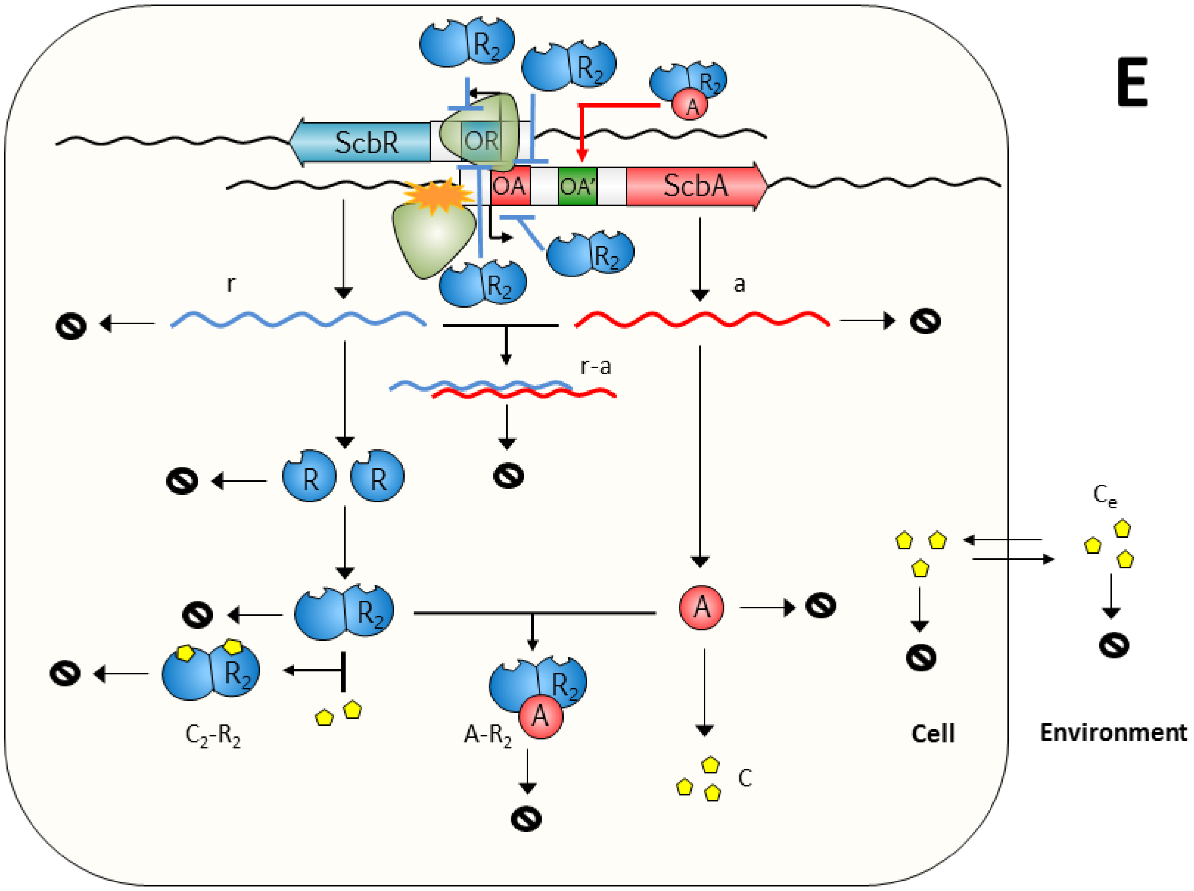
E
ScbR
OR
OA
OA'
ScbA
R2
A
r
a
r-a
R
C
Ce
C2-R2
A-R2
Cell
Environment

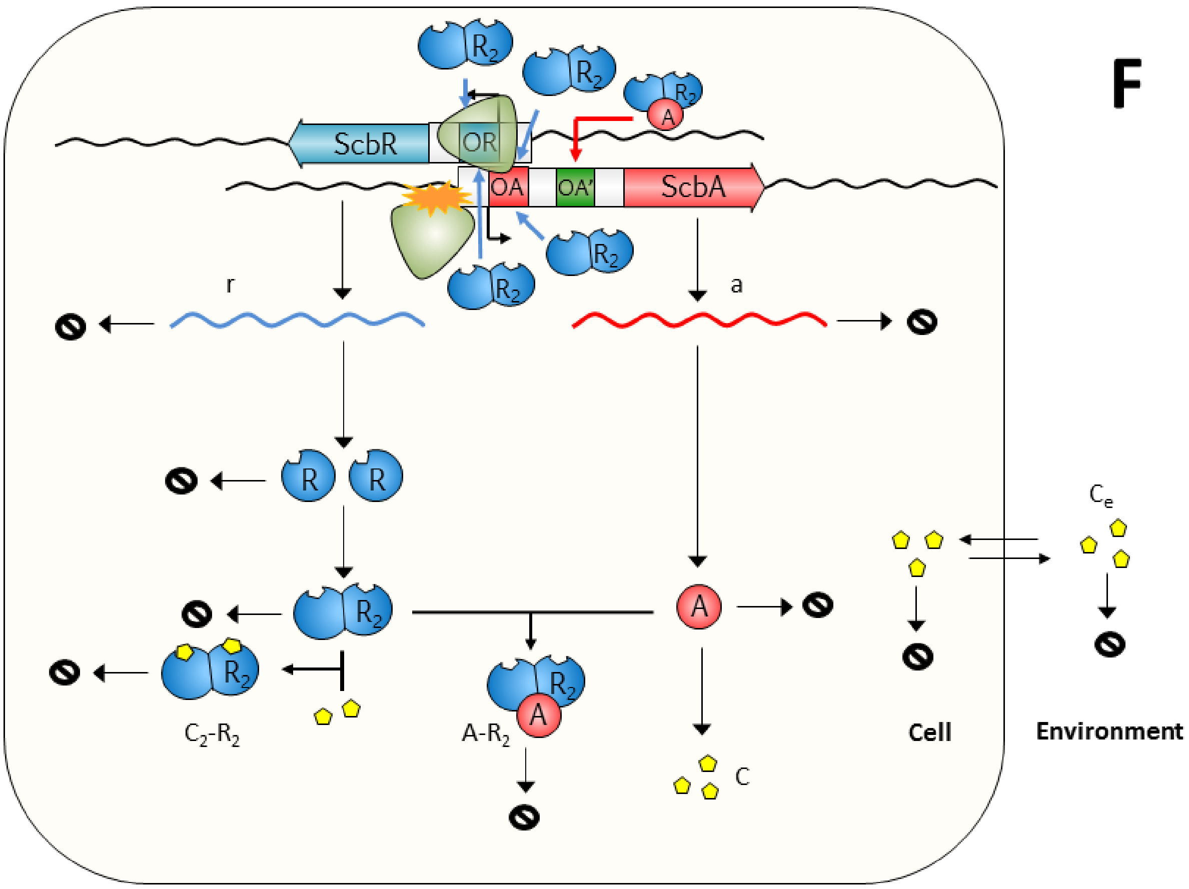
F
ScbR
OR
OA
OA'
ScbA
r
a
R
R
R_2
A
C_2-R_2
A-R_2
C
C_e
Cell
Environment

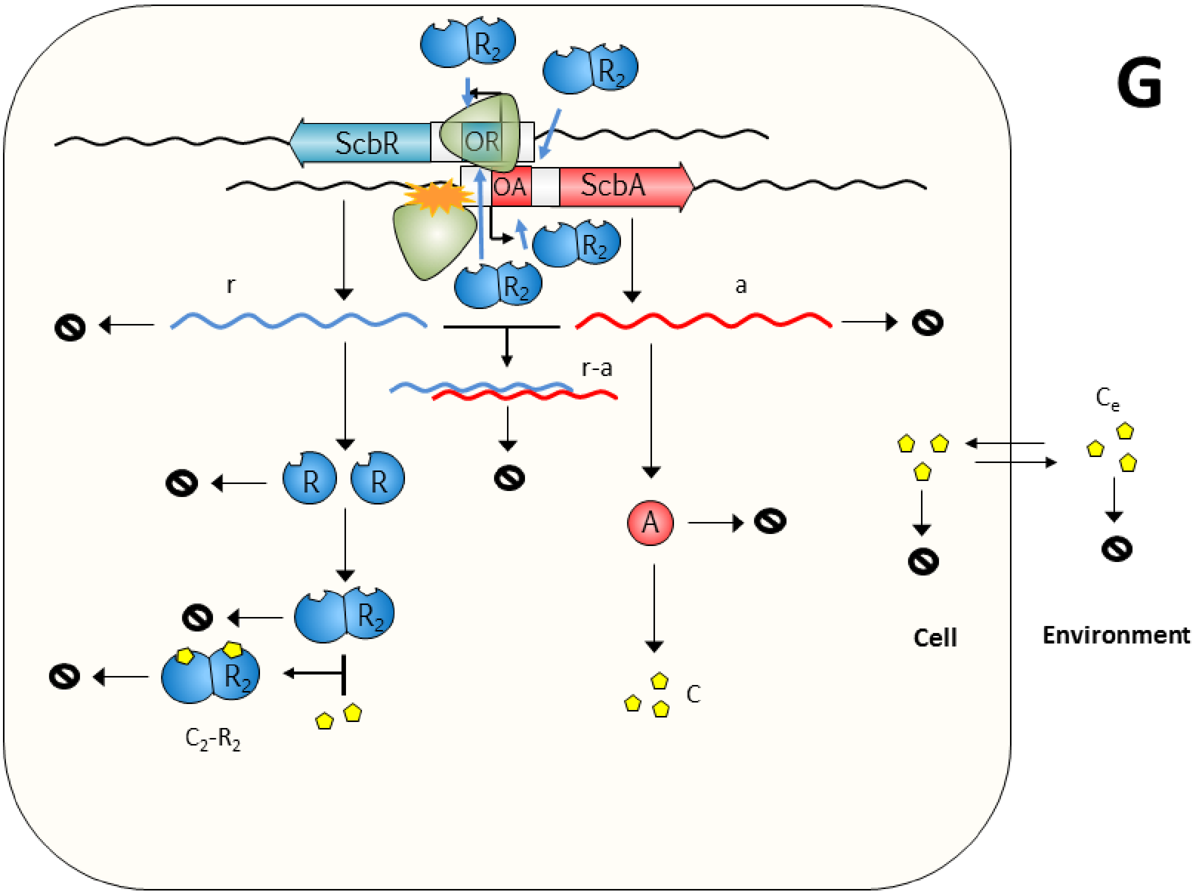
G
ScbR
OR
OA
ScbA
r
a
r-a
R
R
R_2
A
C_2-R_2
C
C_e
Cell
Environment

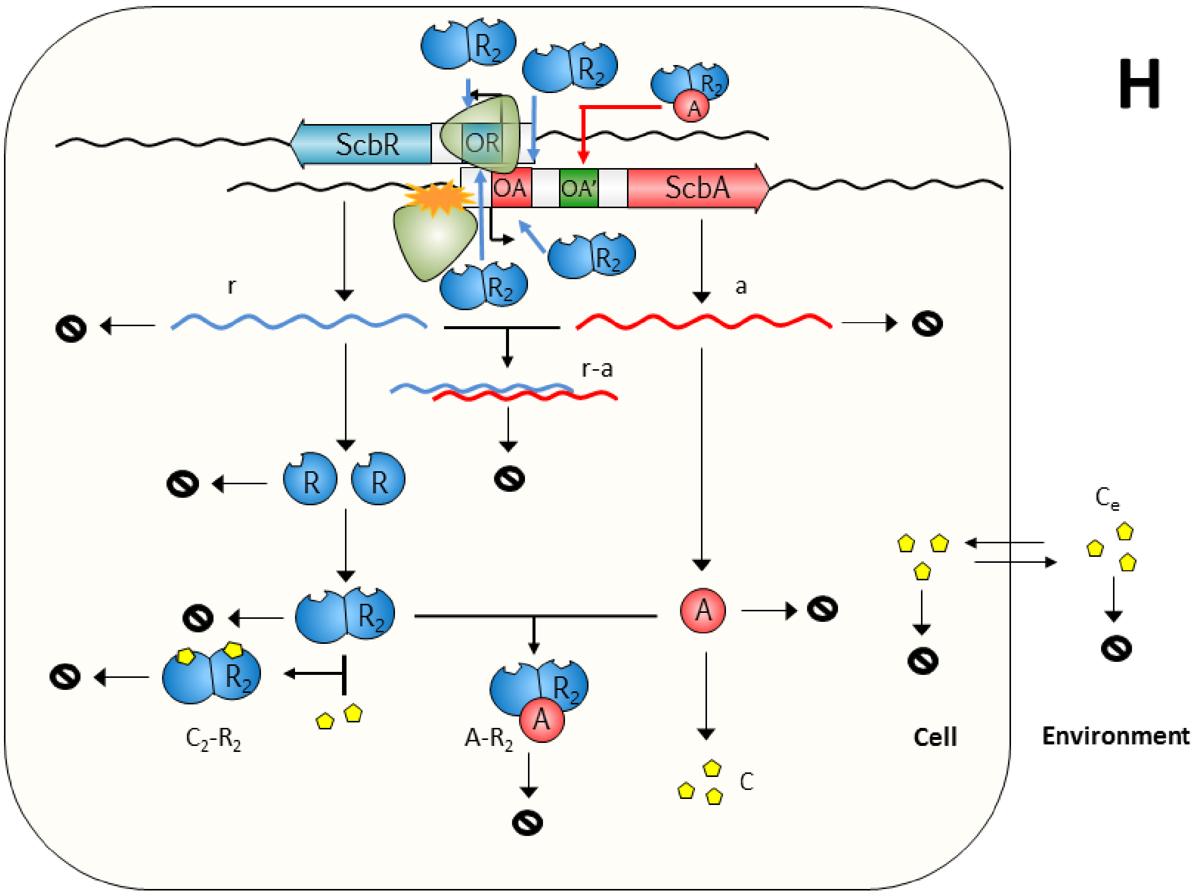

Supplementary Sp1 Fig: Schematic representation of the potential mechanisms of the ScbA/ScbR system for scenarios A-G

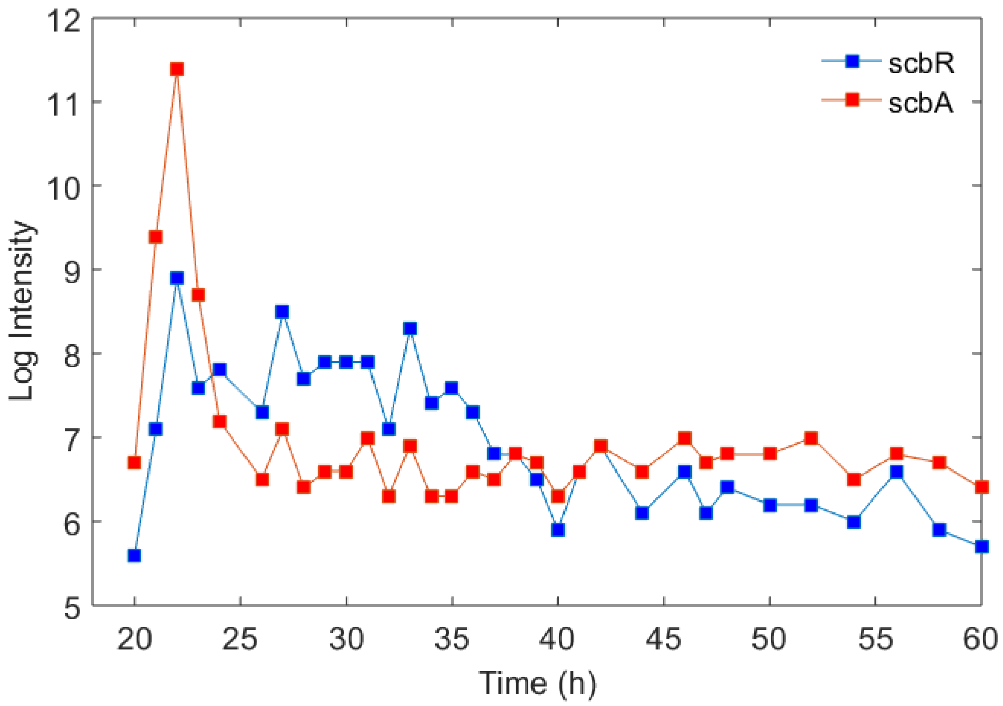

Supplementary Sp2 Fig: Transcriptomics data for *scbR* and *scbA* genes as reported in the publication by Nieselt *et al.* as normalized values of the corresponding microarray probe set for each time point. These data provided the criteria that were used to assess the quality of the models.

Complete log-likelihood profiles

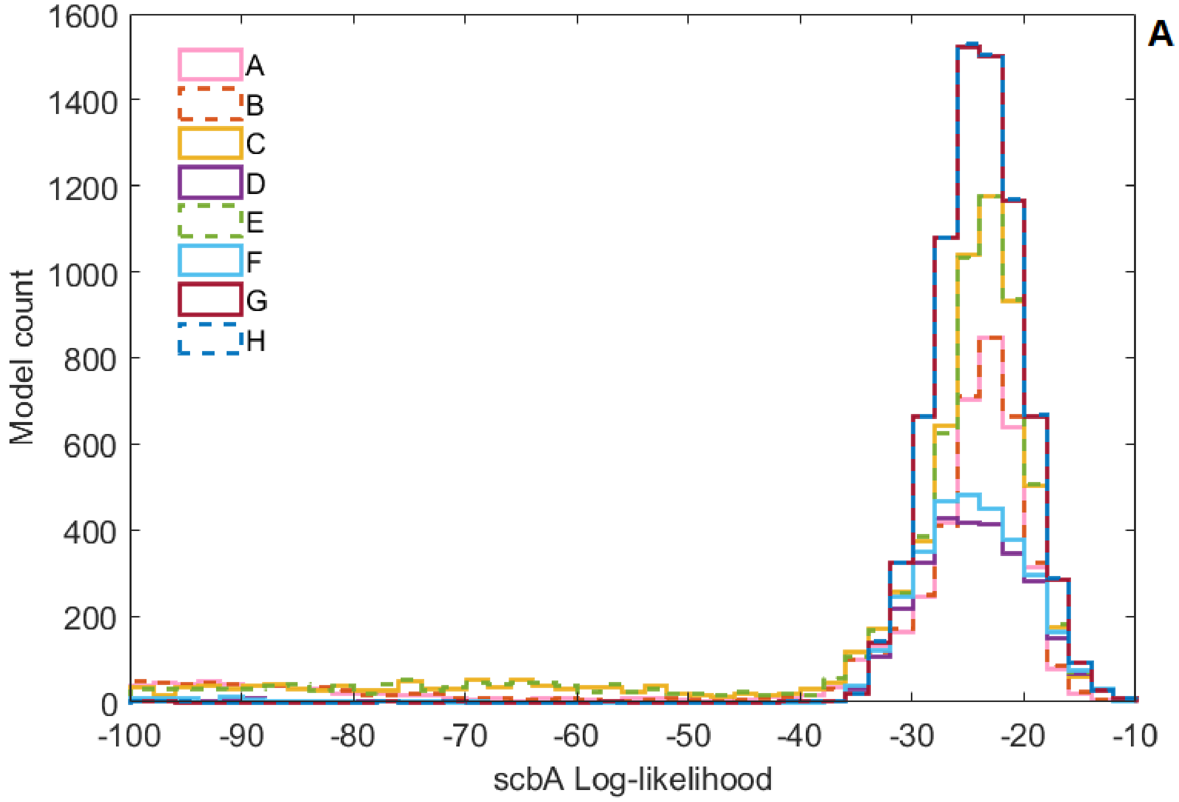

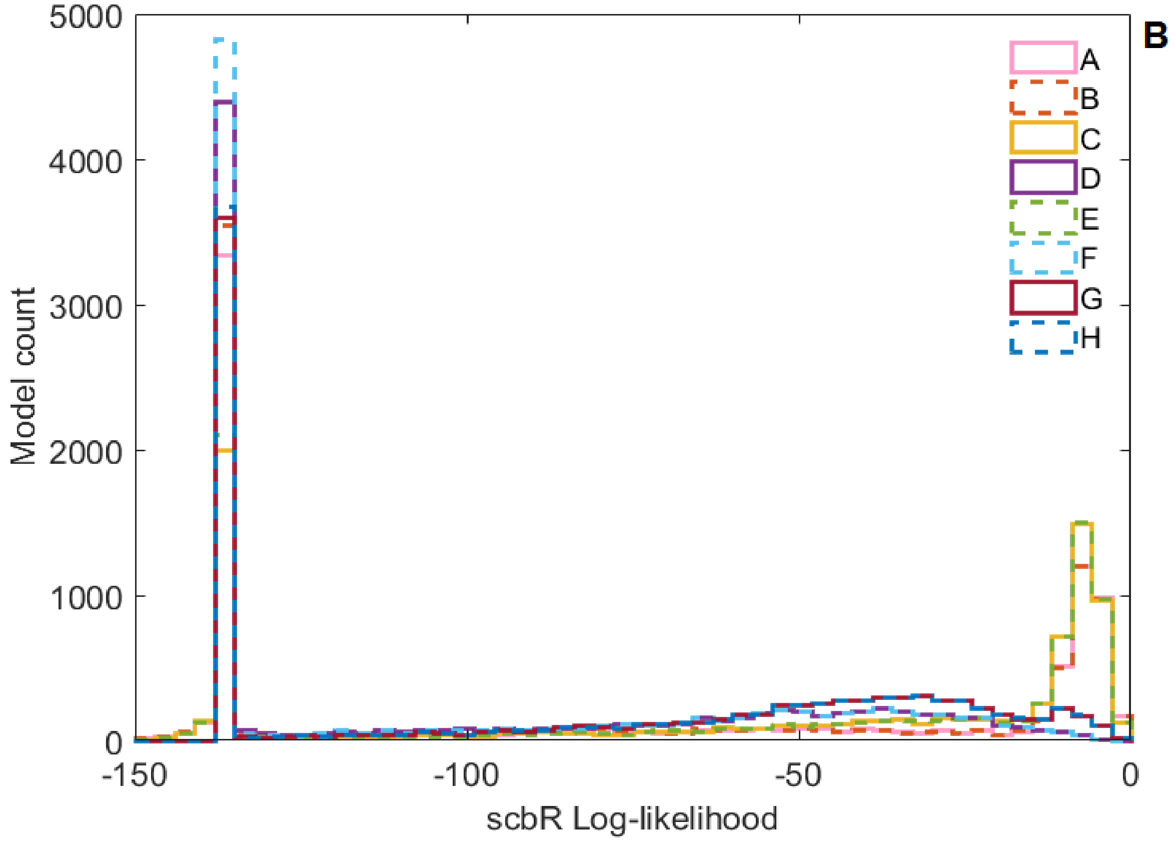

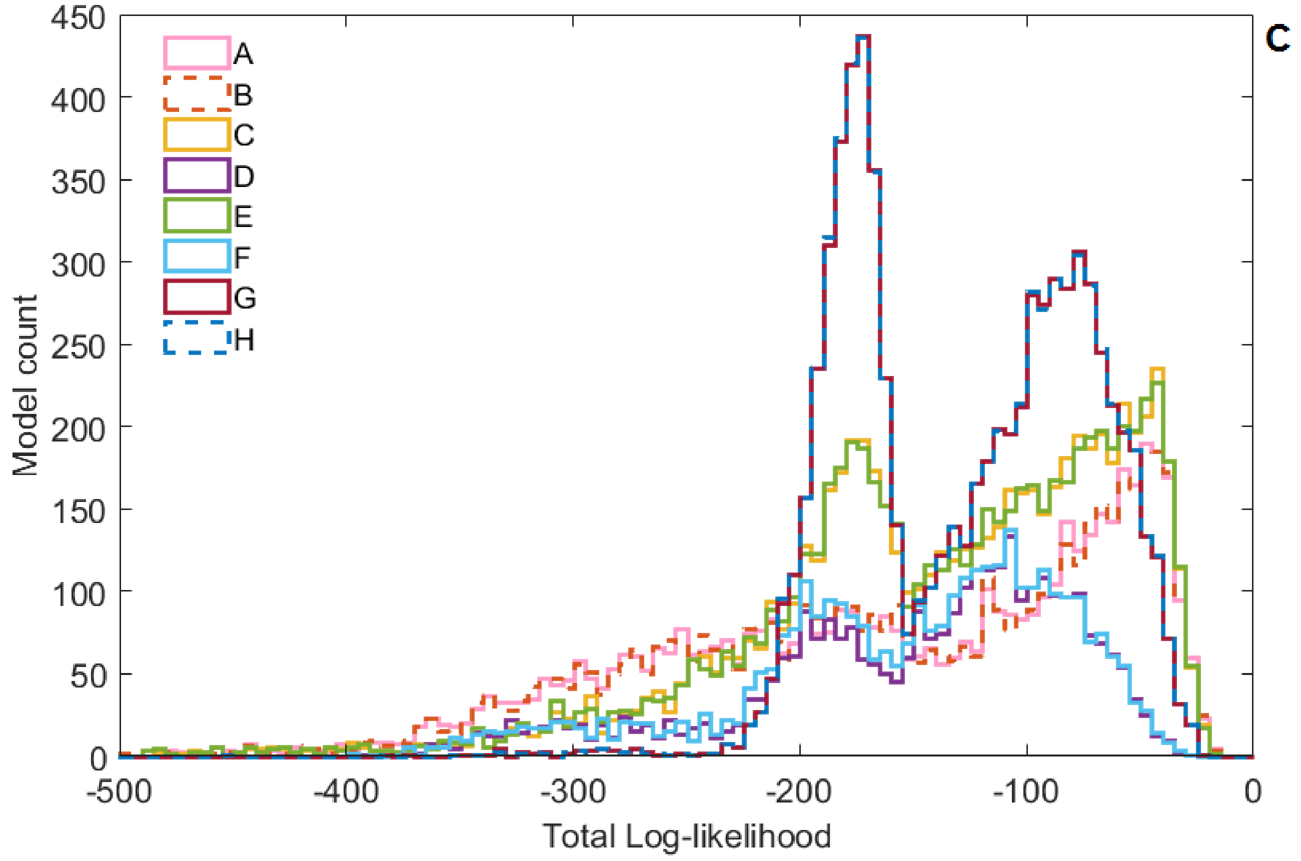

Supplementary Sp3 Fig: Complete log-likelihood profiles (*scbA* (A), *scbR* (B) and total (C)) for the 8 scenarios of the GBL model, with promoters having equal strength (x = 1).

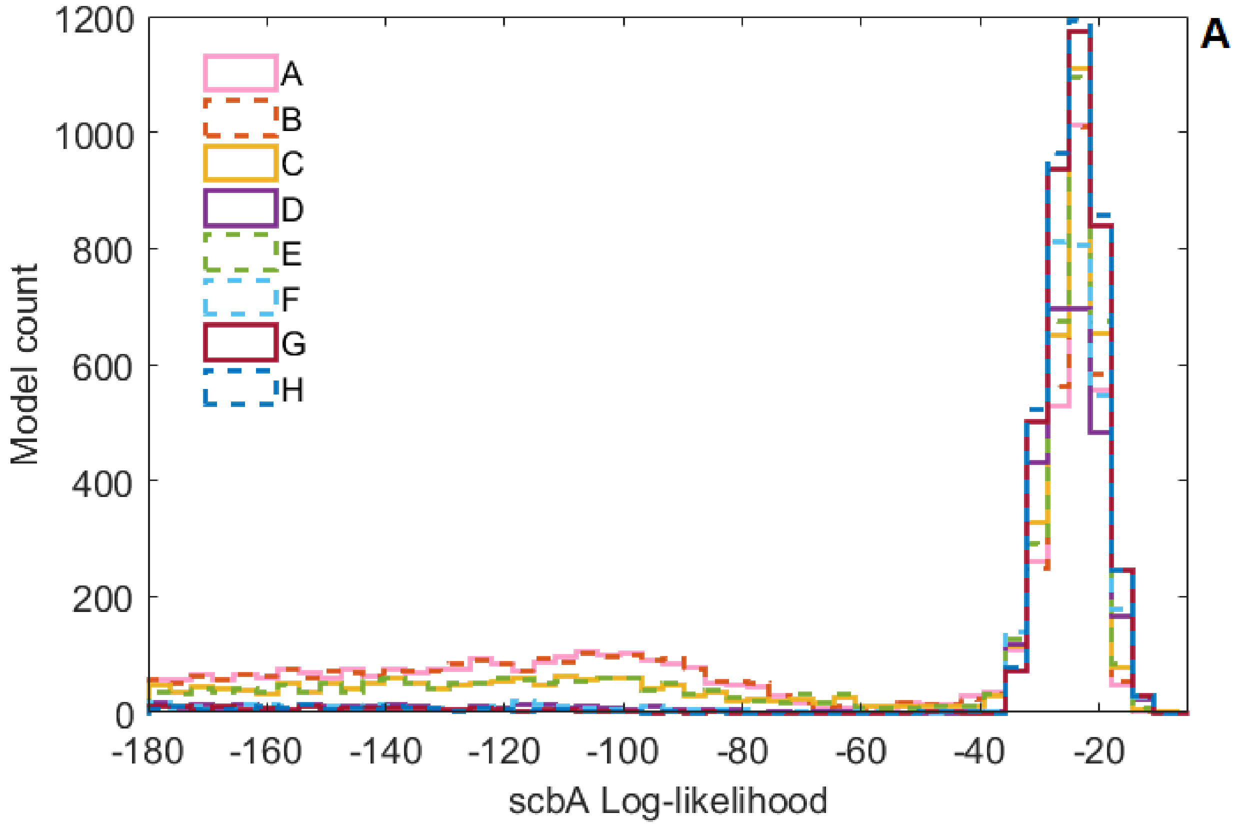

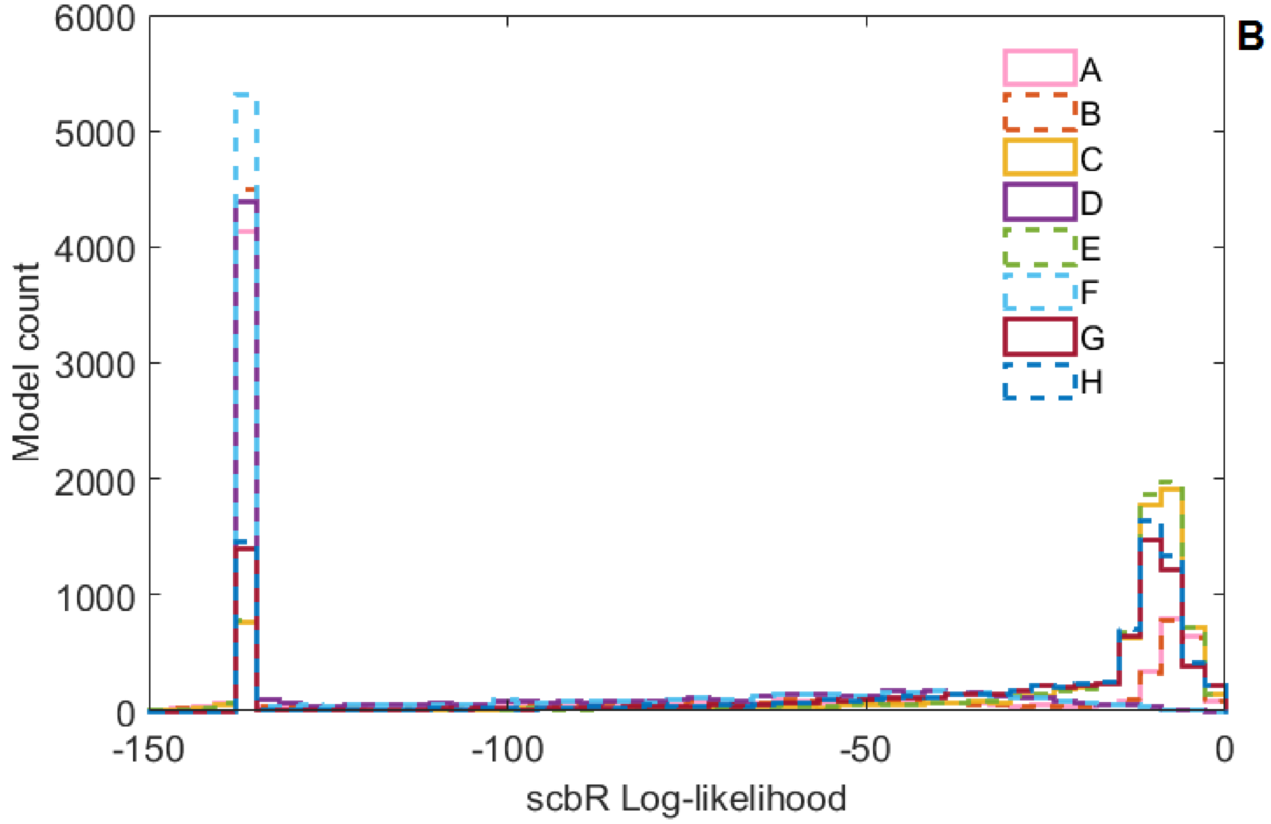

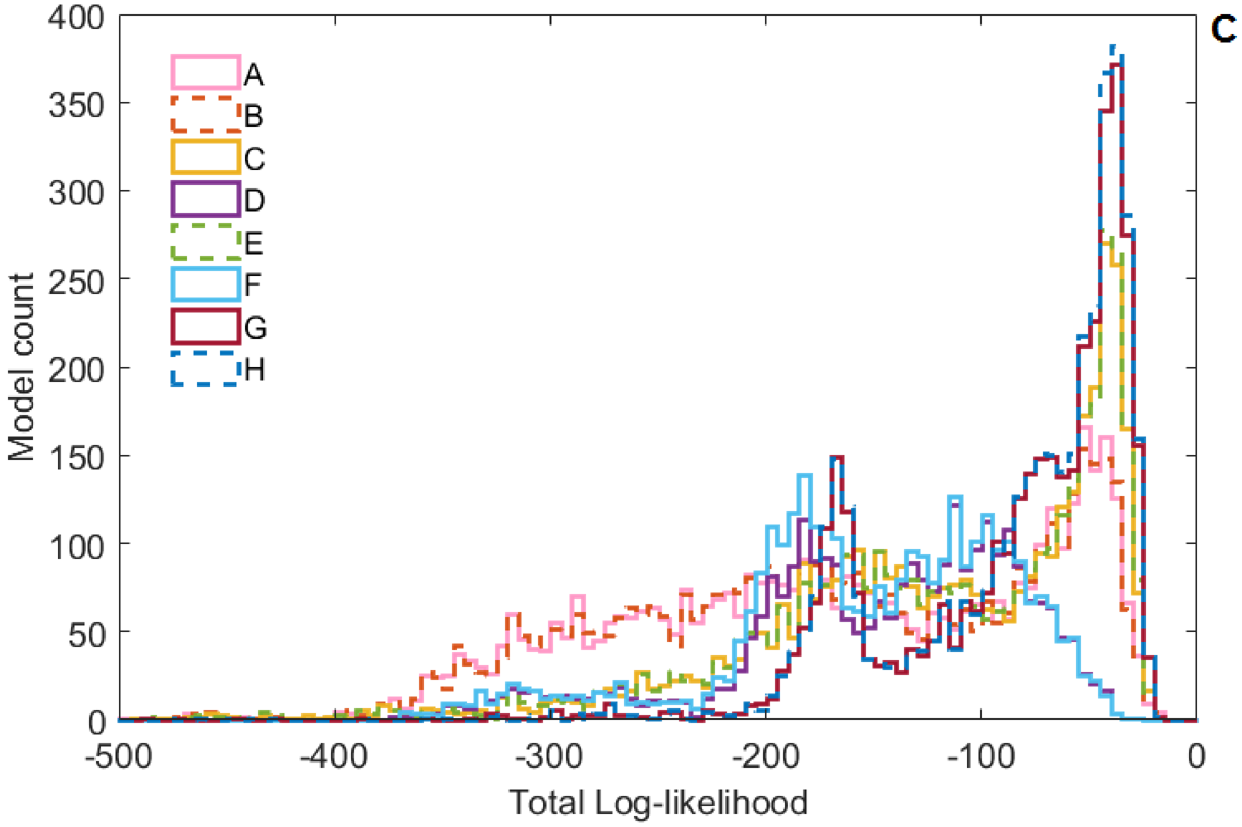

Supplementary Sp4 Fig: Complete log-likelihood profiles (*scbA* (A), *scbR* (B) and total (C)) for the eight scenarios of the GBL model, with *scbA* promoter being stronger (x= 0.1 – 0.9).

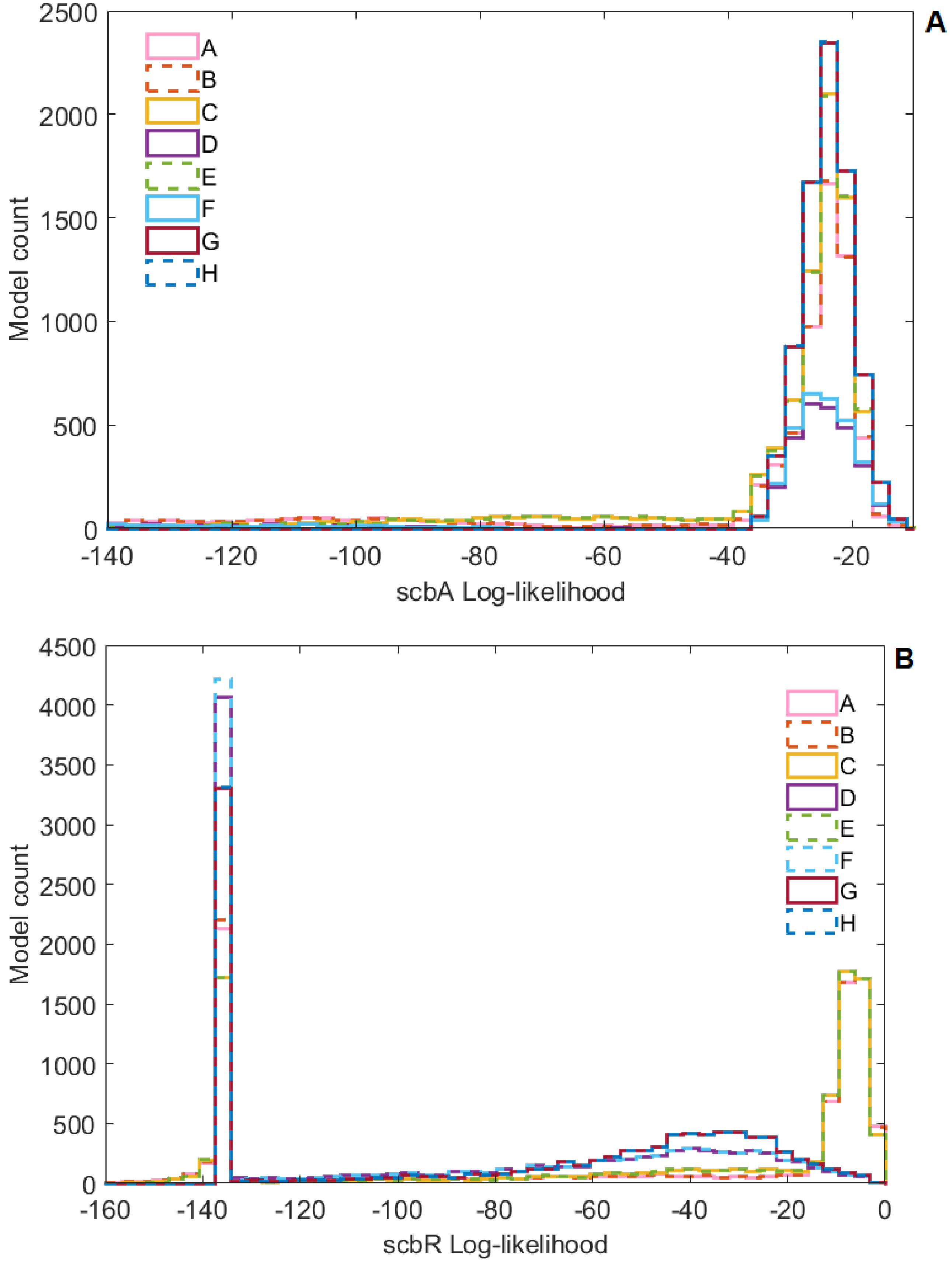
A
Model count
scbA Log-likelihood
A
B
C
D
E
F
G
H
B
Model count
scbR Log-likelihood
A
B
C
D
E
F
G
H

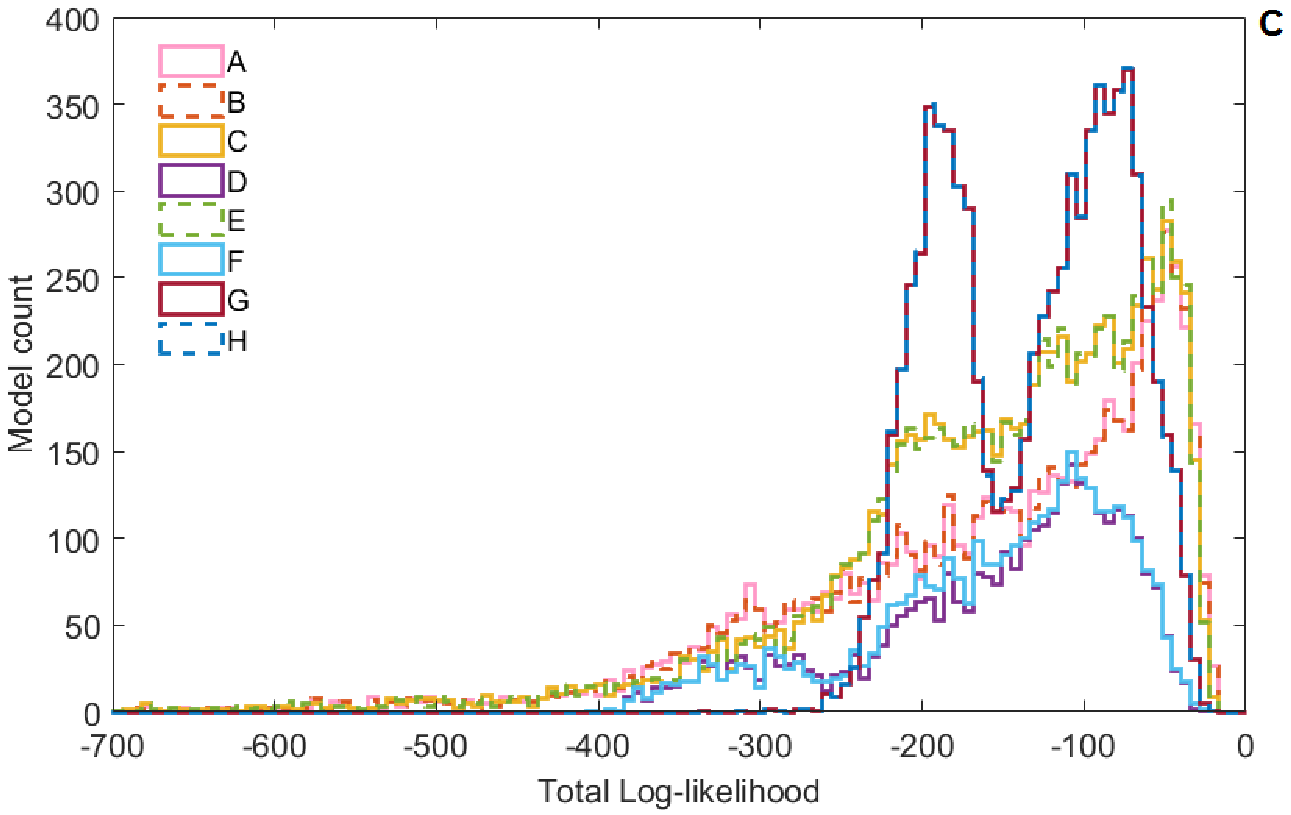

Supplementary Sp5 Fig: Complete log-likelihood profiles (*scbA* (A), *scbR* (B) and total (C)) for the eight scenarios of the GBL model, with *scbR* promoter being stronger (x= 1 - 10).

Parameter analysis

Scenario A (k_{FR}=K_{FA})

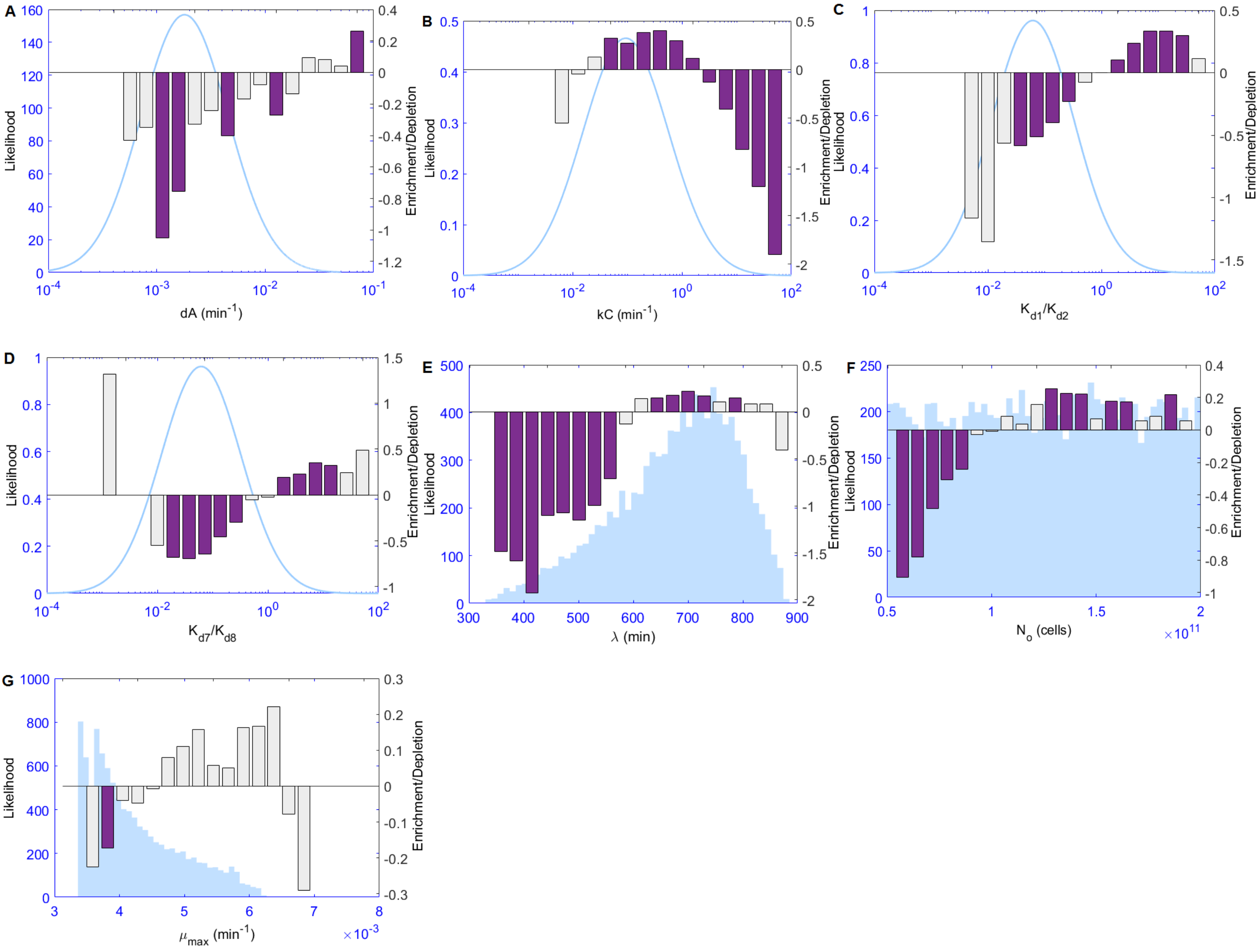

Supplementary Sp6 Fig: Comparison between the expected parameter values according to the defined priors and the actual parameters of the best models (TLL > −140) for Scenario A with promoters of equal strength.

Scenario A (k_{FR}>K_{FA})

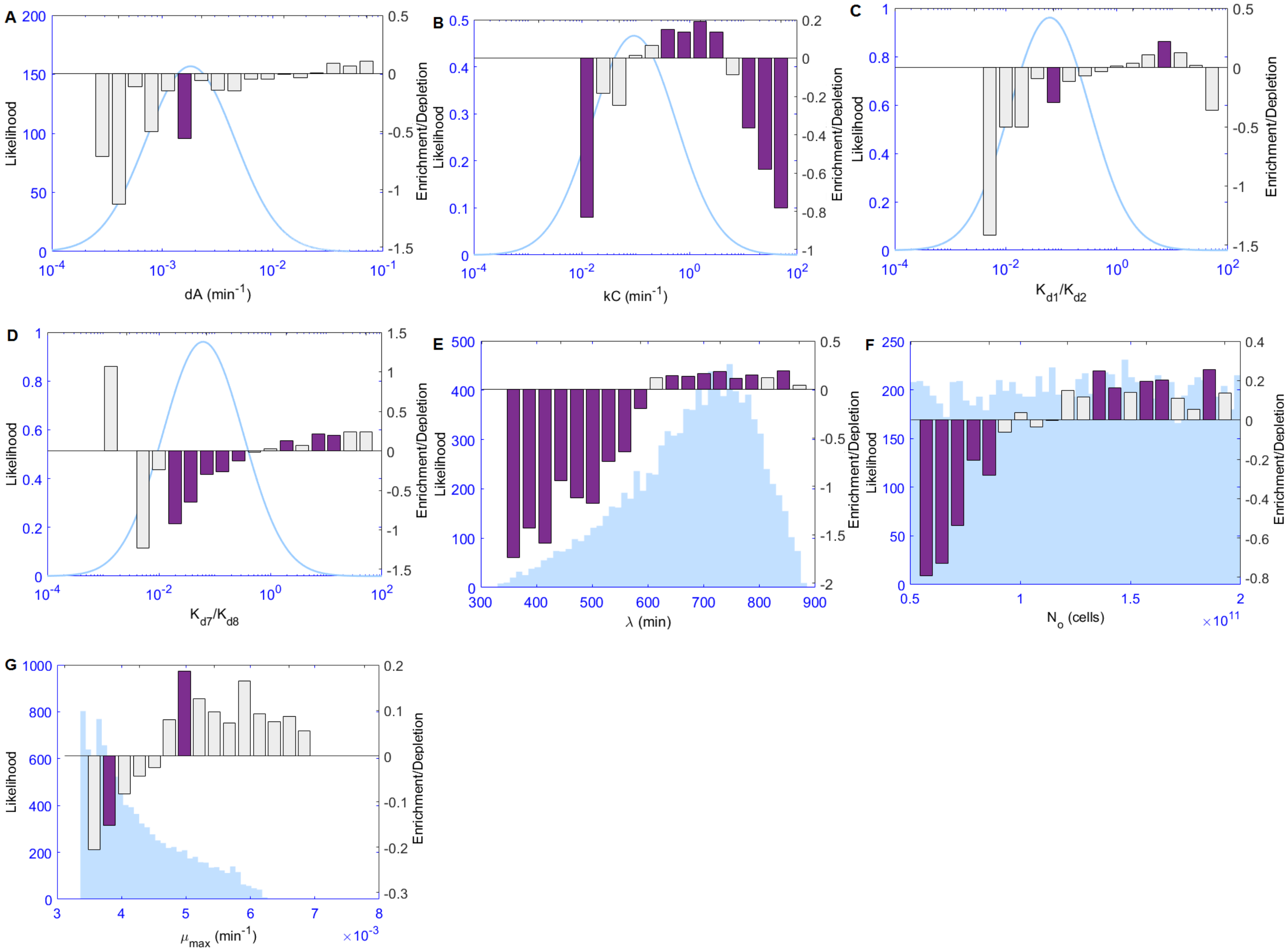

Supplementary Sp7 Fig: Comparison between the expected parameter values according to the defined priors and the actual parameters of the best models (TLL > −140) for Scenario A with *scbR* promoter being stronger than *scbA*.

Scenario A (k_{FR}<K_{FA})

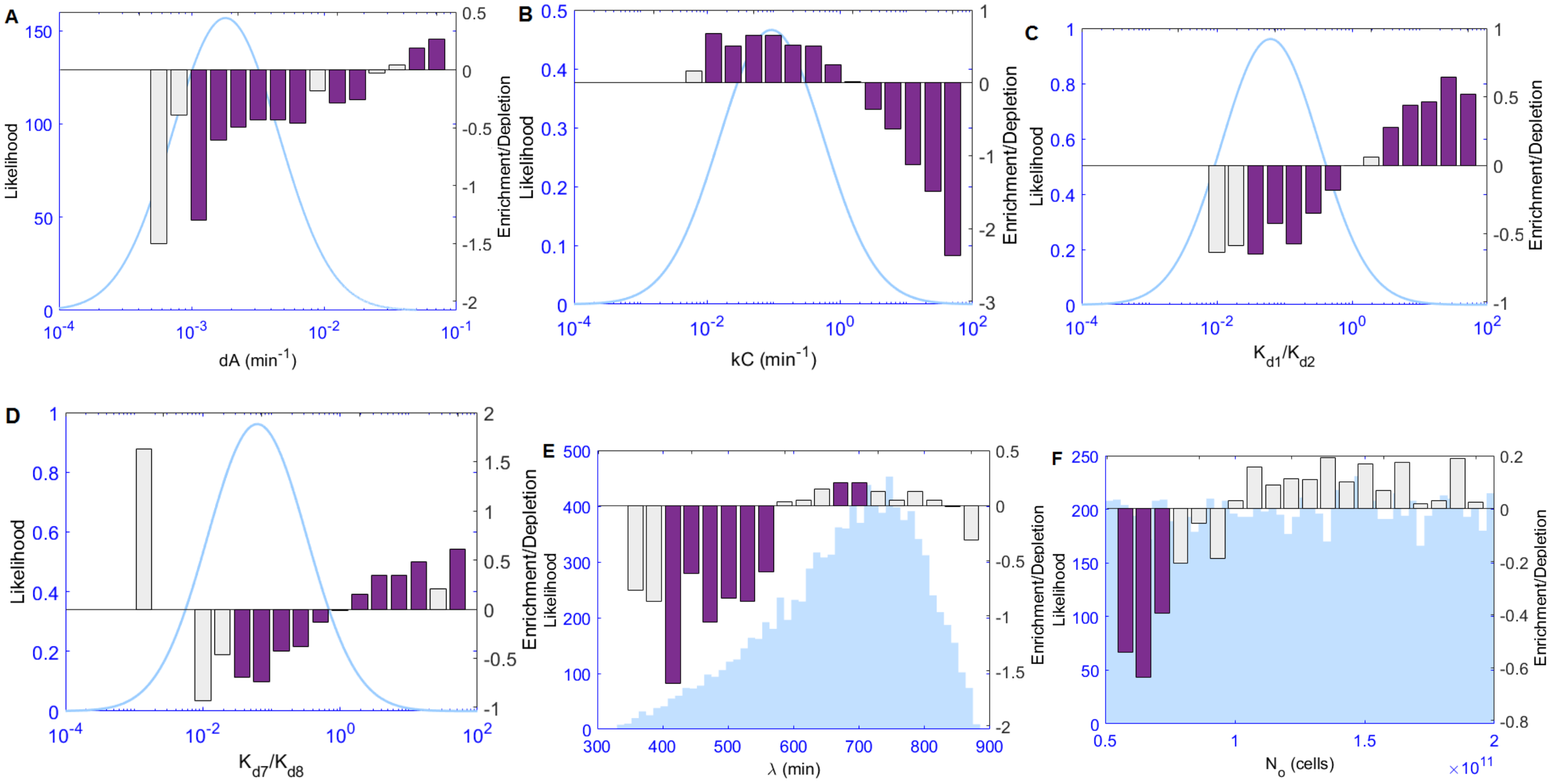

Supplementary Sp8 Fig: Comparison between the expected parameter values according to the defined priors and the actual parameters of the best models (TLL > −140) for Scenario A with *scbA* promoter being stronger than *scbR*.

Scenario B (k_{FR}=K_{FA})

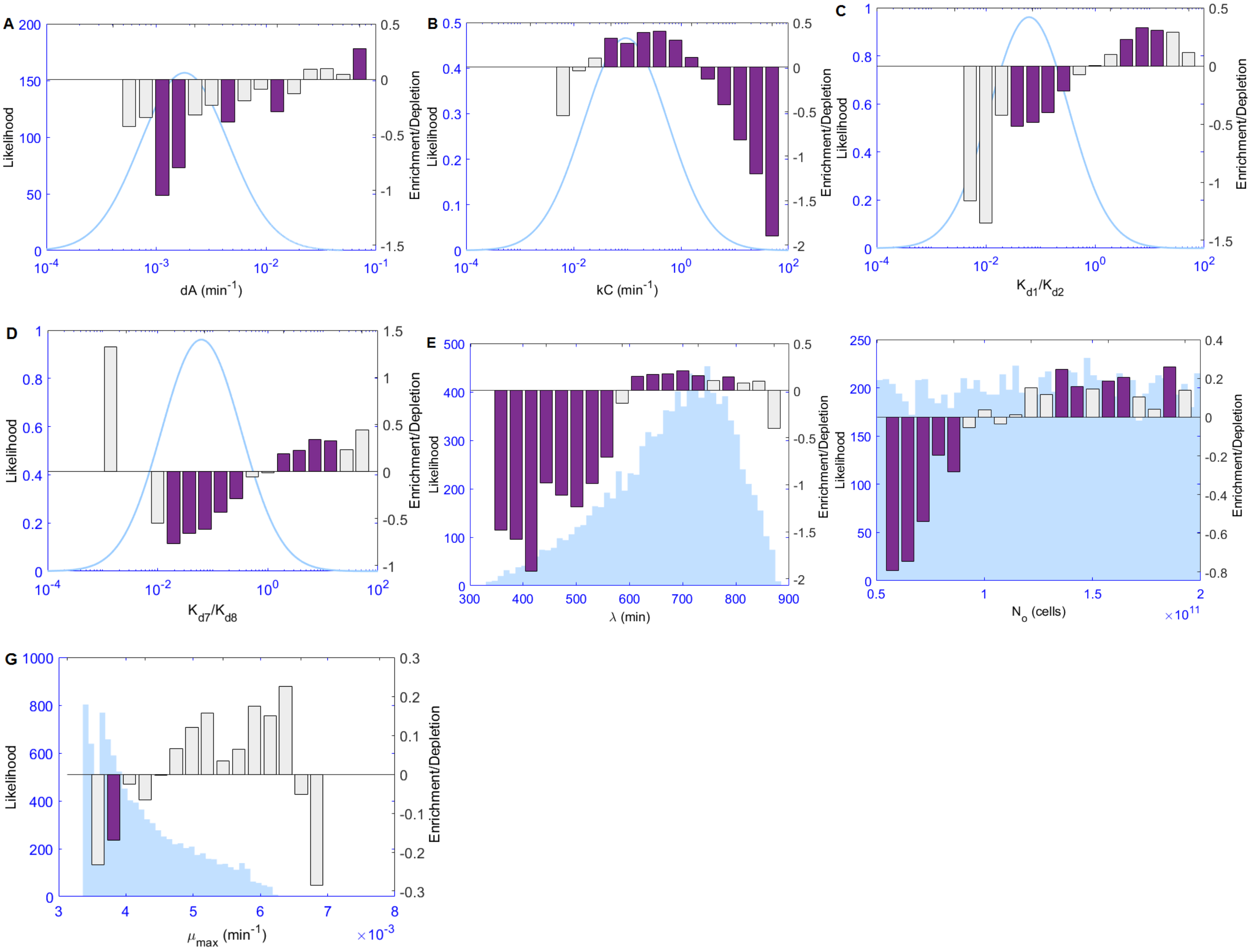

Supplementary Sp9 Fig: Comparison between the expected parameter values according to the defined priors and the actual parameters of the best models (TLL > −140) for Scenario B with promoters of equal strength.

Scenario B (k_{FR}>K_{FA})

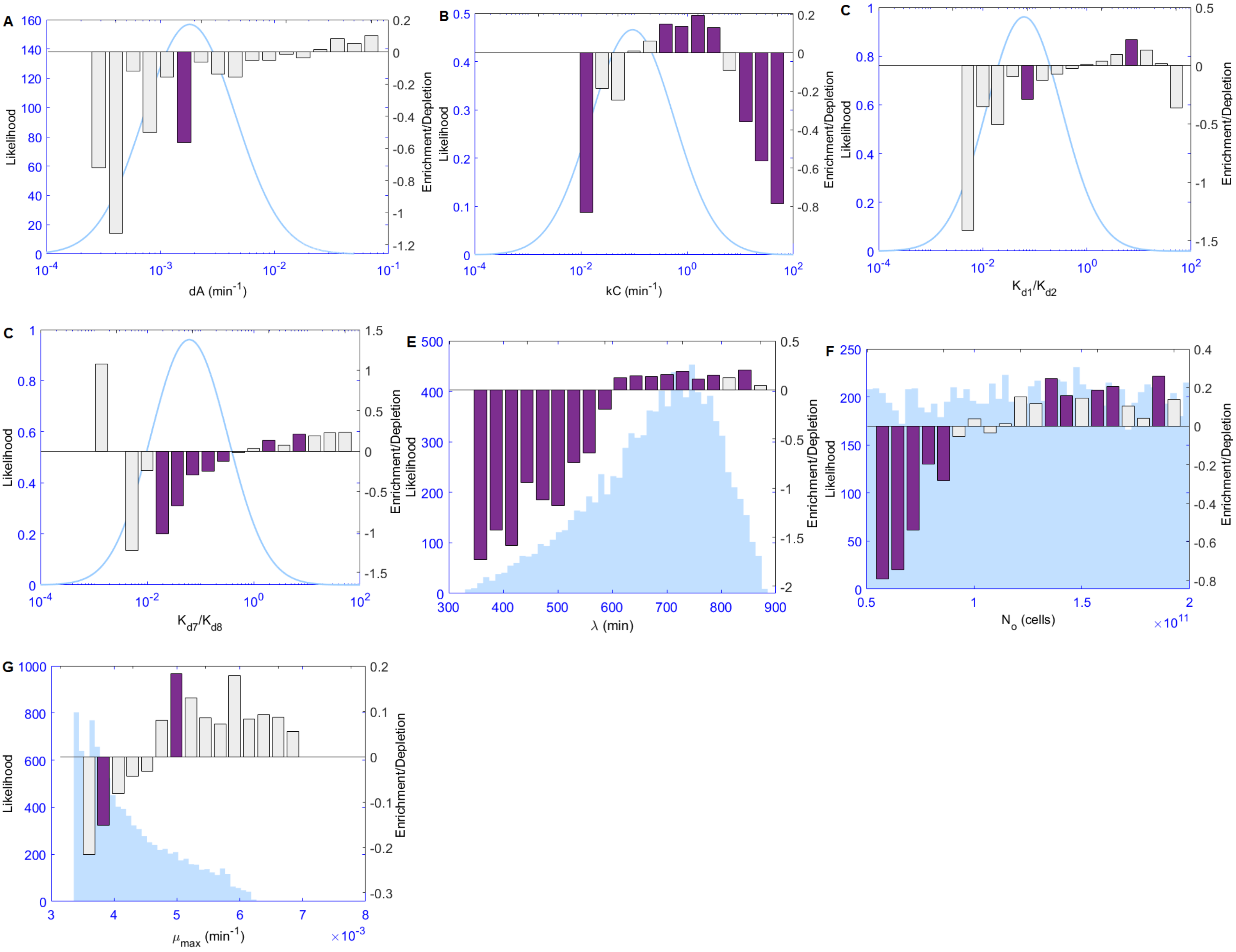

Supplementary Sp10 Fig: Comparison between the expected parameter values according to the defined priors and the actual parameters of the best models (TLL > −140) for Scenario B with *scbR* promoter being stronger than *scbA*.

Scenario B ($k_{FR}<K_{FA}$)

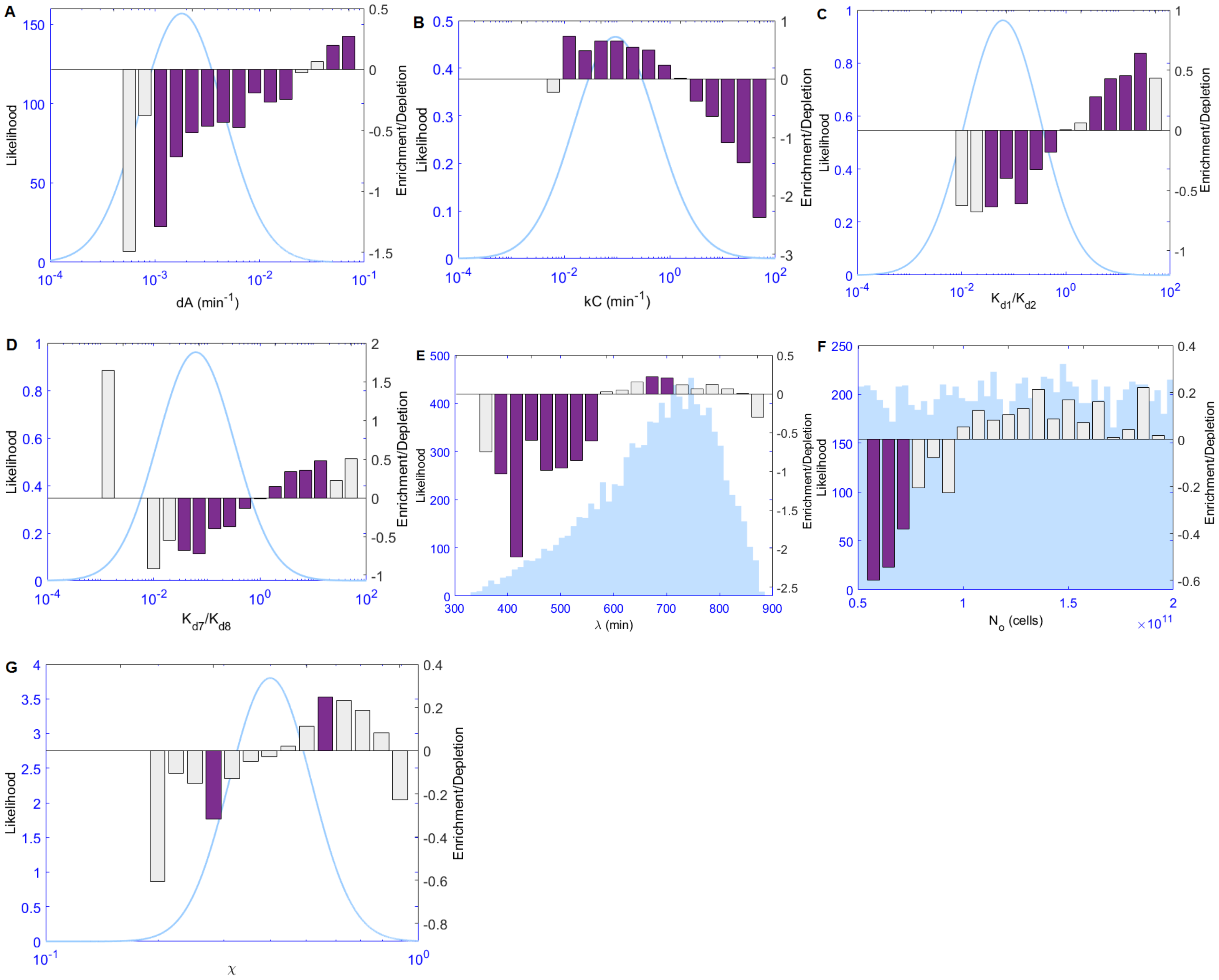

Supplementary Sp11 Fig: Comparison between the expected parameter values according to the defined priors and the actual parameters of the best models (TLL > −140) for Scenario B with *scbA* promoter being stronger than *scbR*.

Scenario C (k_{FR}=K_{FA})

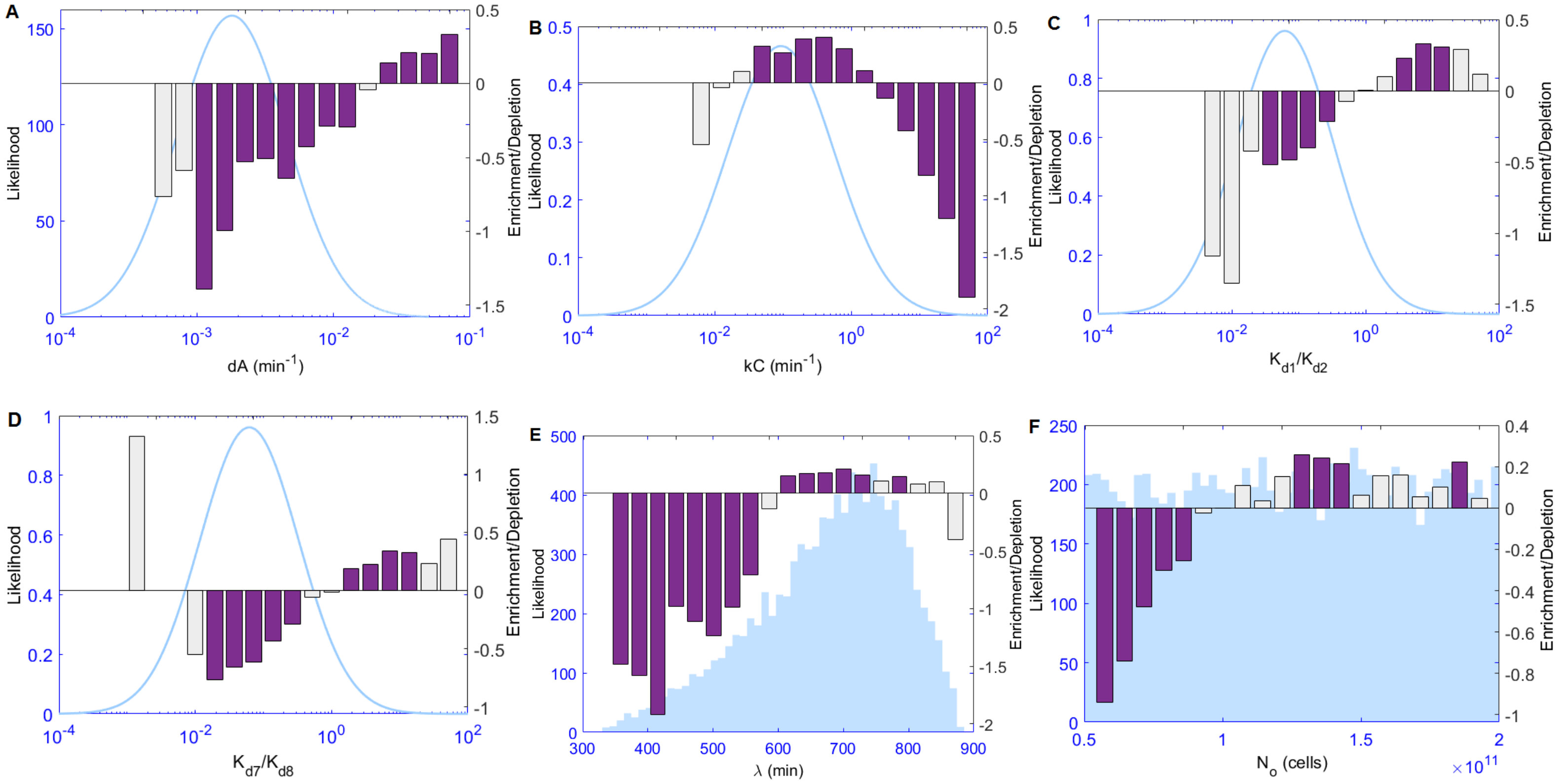

Supplementary Sp12 Fig: Comparison between the expected parameter values according to the defined priors and the actual parameters of the best models (TLL > −140) for Scenario C with promoters of equal strength.

Scenario C (k_{FR}>K_{FA})

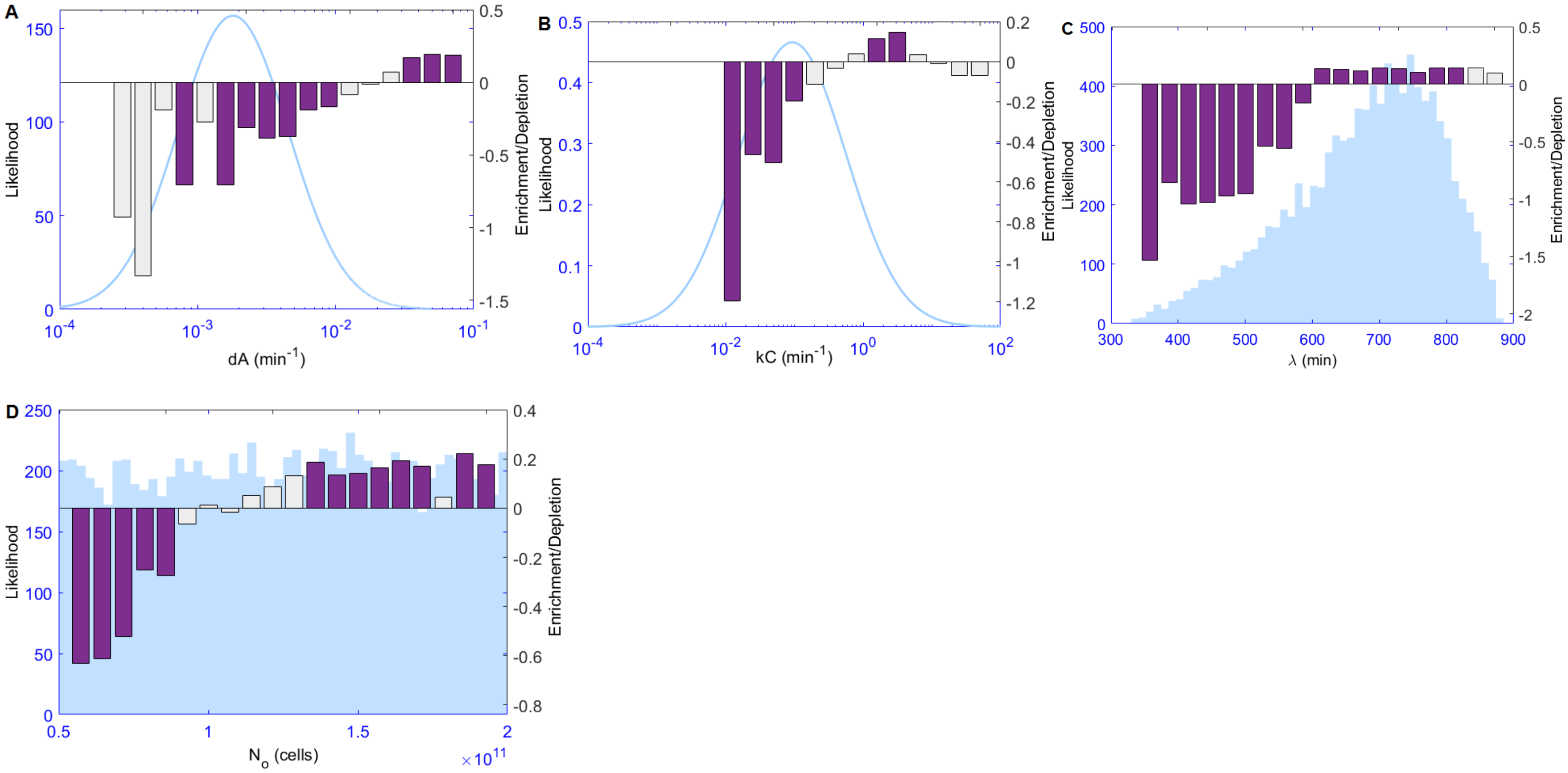

Supplementary Sp13 Fig: Comparison between the expected parameter values according to the defined priors and the actual parameters of the best models (TLL > −140) for Scenario C with *scbR* promoter being stronger than *scbA*.

Scenario C (k_{FR}<K_{FA})

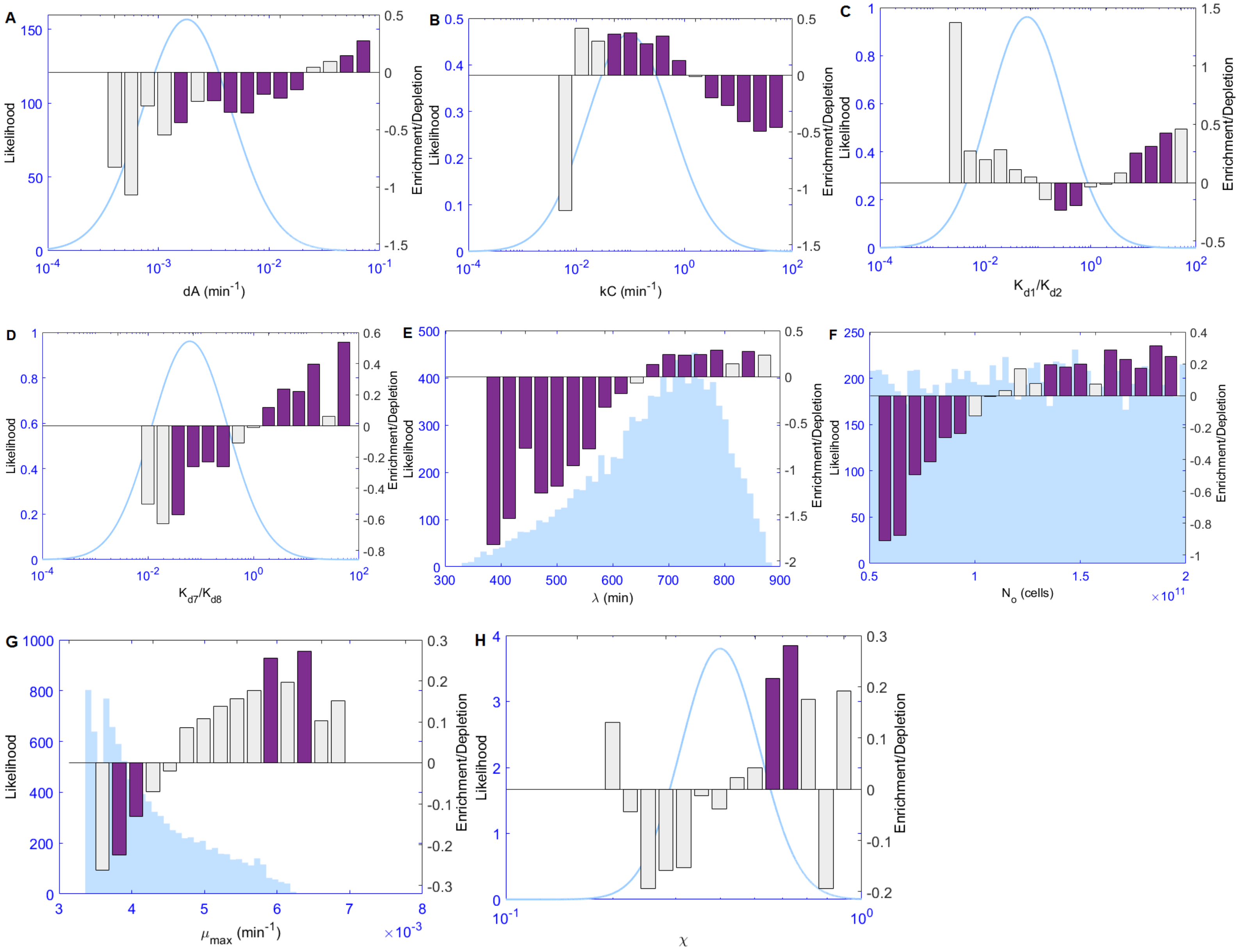

Supplementary Sp14 Fig: Comparison between the expected parameter values according to the defined priors and the actual parameters of the best models (TLL > −140) for Scenario C with *scbA* promoter being stronger than *scbR*.

Scenario D (k_{FR}=K_{FA})

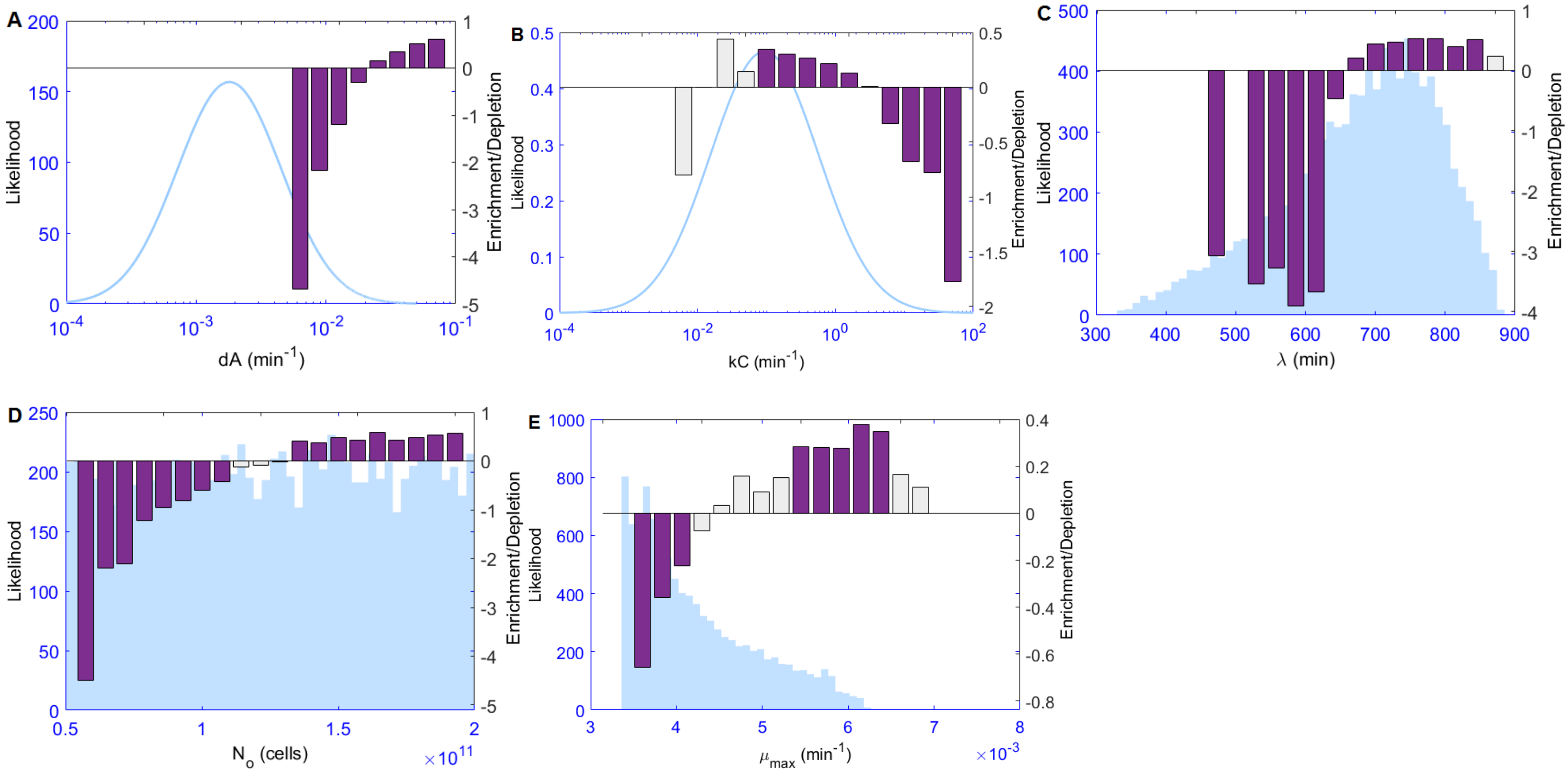

Supplementary Sp15 Fig: Comparison between the expected parameter values according to the defined priors and the actual parameters of the best models (TLL > −140) for Scenario D with promoters of equal strength.

Scenario D (k_{FR}>K_{FA})

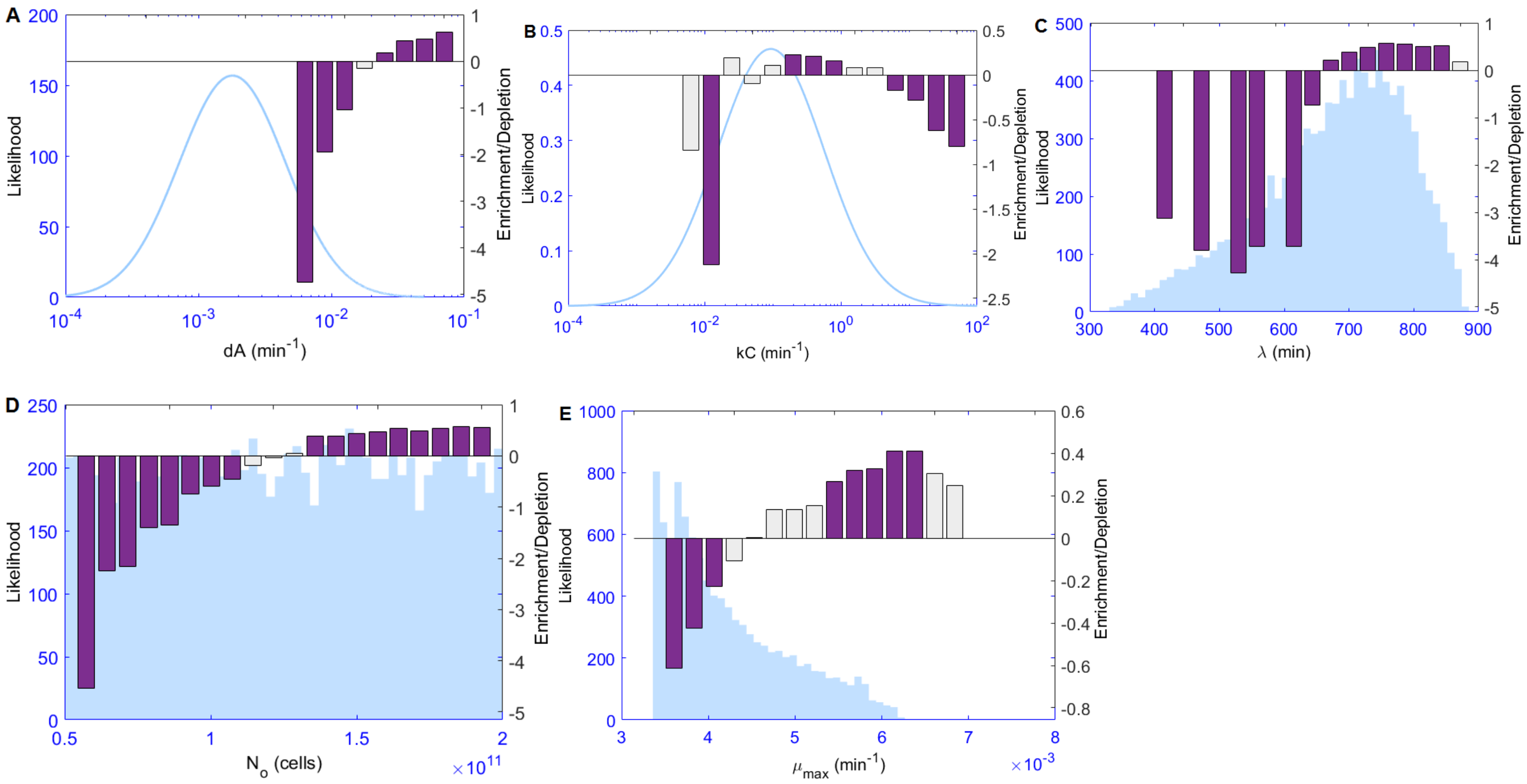

Supplementary Sp16 Fig: Comparison between the expected parameter values according to the defined priors and the actual parameters of the best models (TLL > −140) for Scenario D with *scbR* promoter being stronger than *scbA*.

Scenario D (k_{FR}<K_{FA})

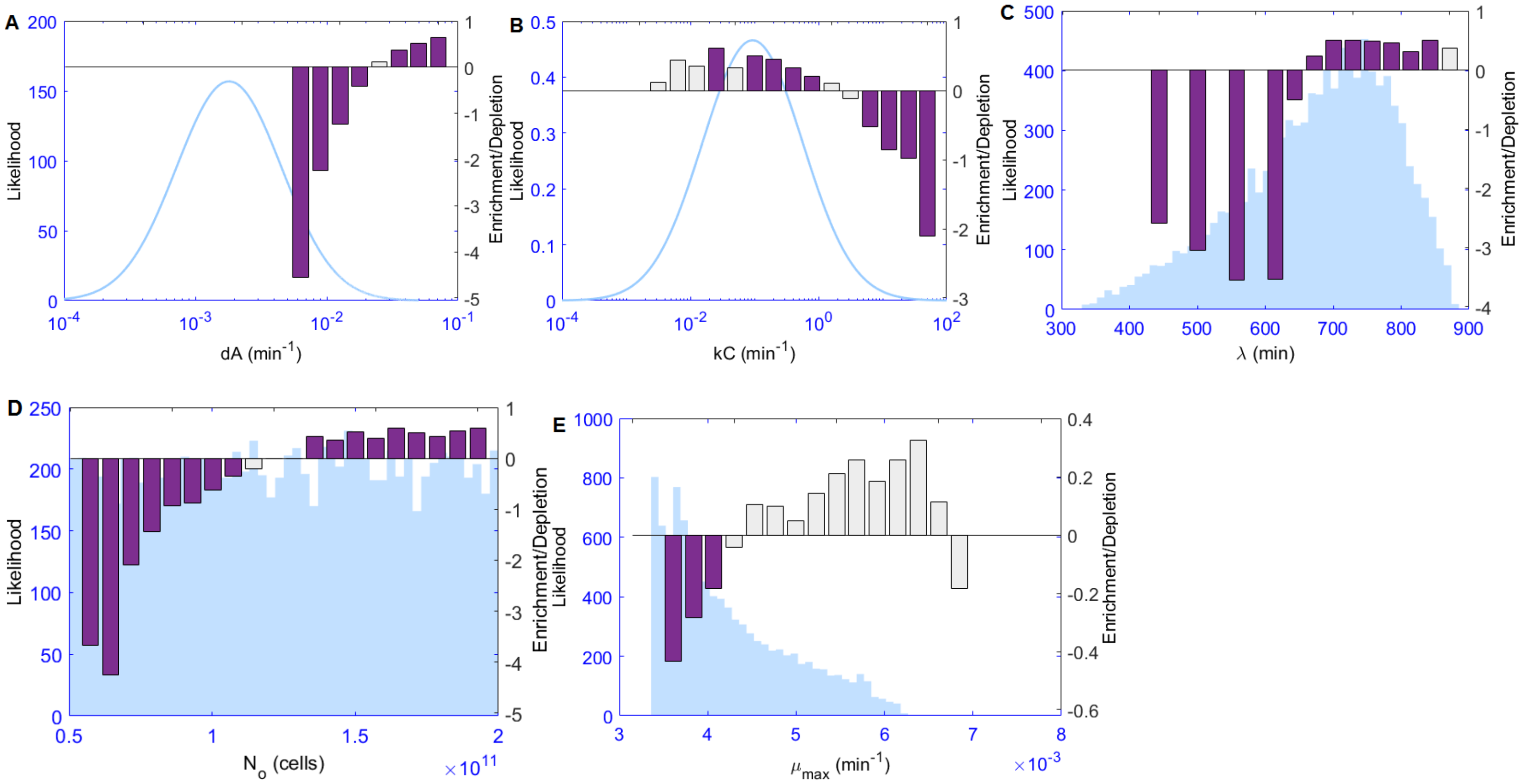

Supplementary Sp17 Fig: Comparison between the expected parameter values according to the defined priors and the actual parameters of the best models (TLL > −140) for Scenario D with *scbA* promoter being stronger than *scbR*.

Scenario E (k_{FR}=K_{FA})

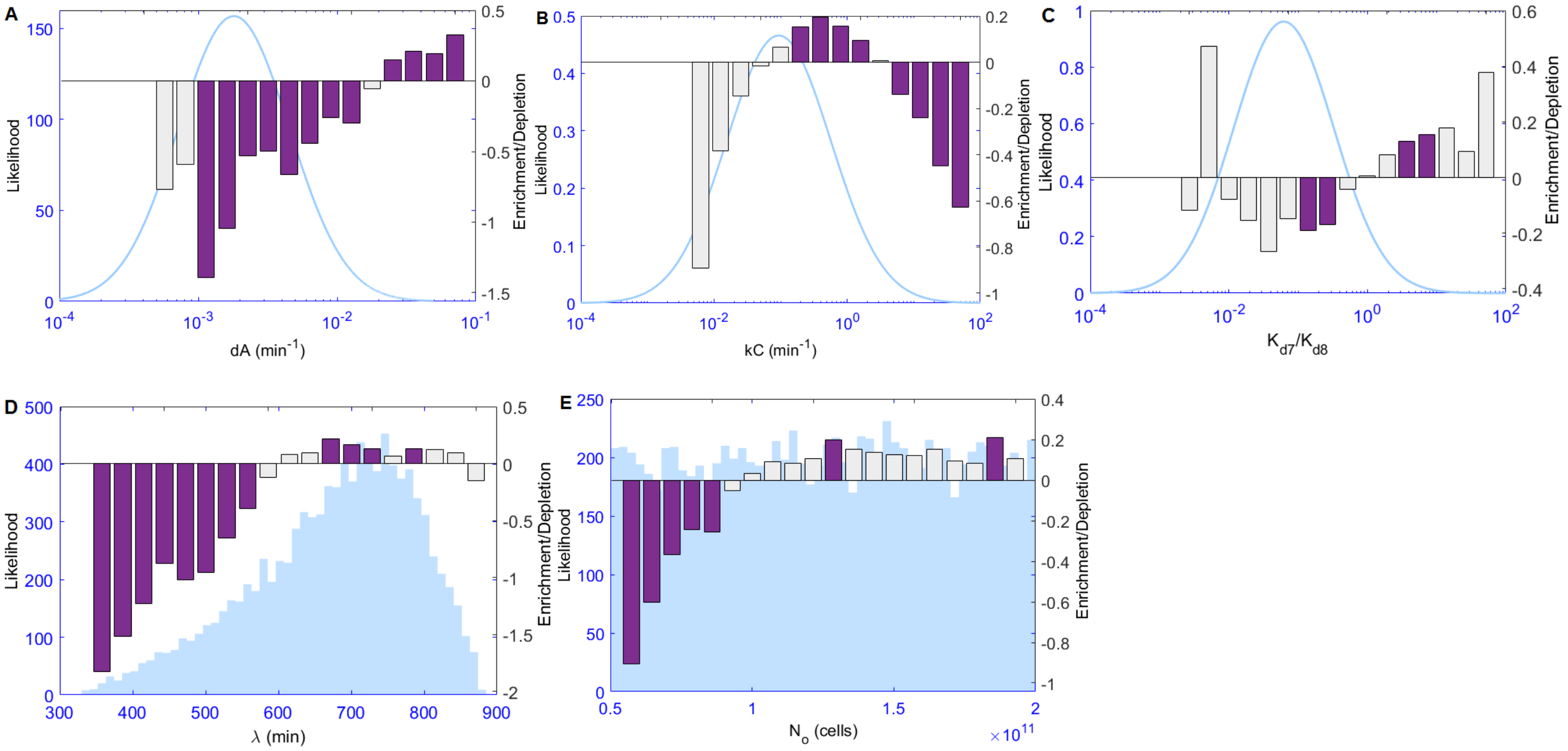

Supplementary Sp18 Fig: Comparison between the expected parameter values according to the defined priors and the actual parameters of the best models (TLL > −140) for Scenario E with promoters of equal strength.

Scenario E (k_{FR}>K_{FA})

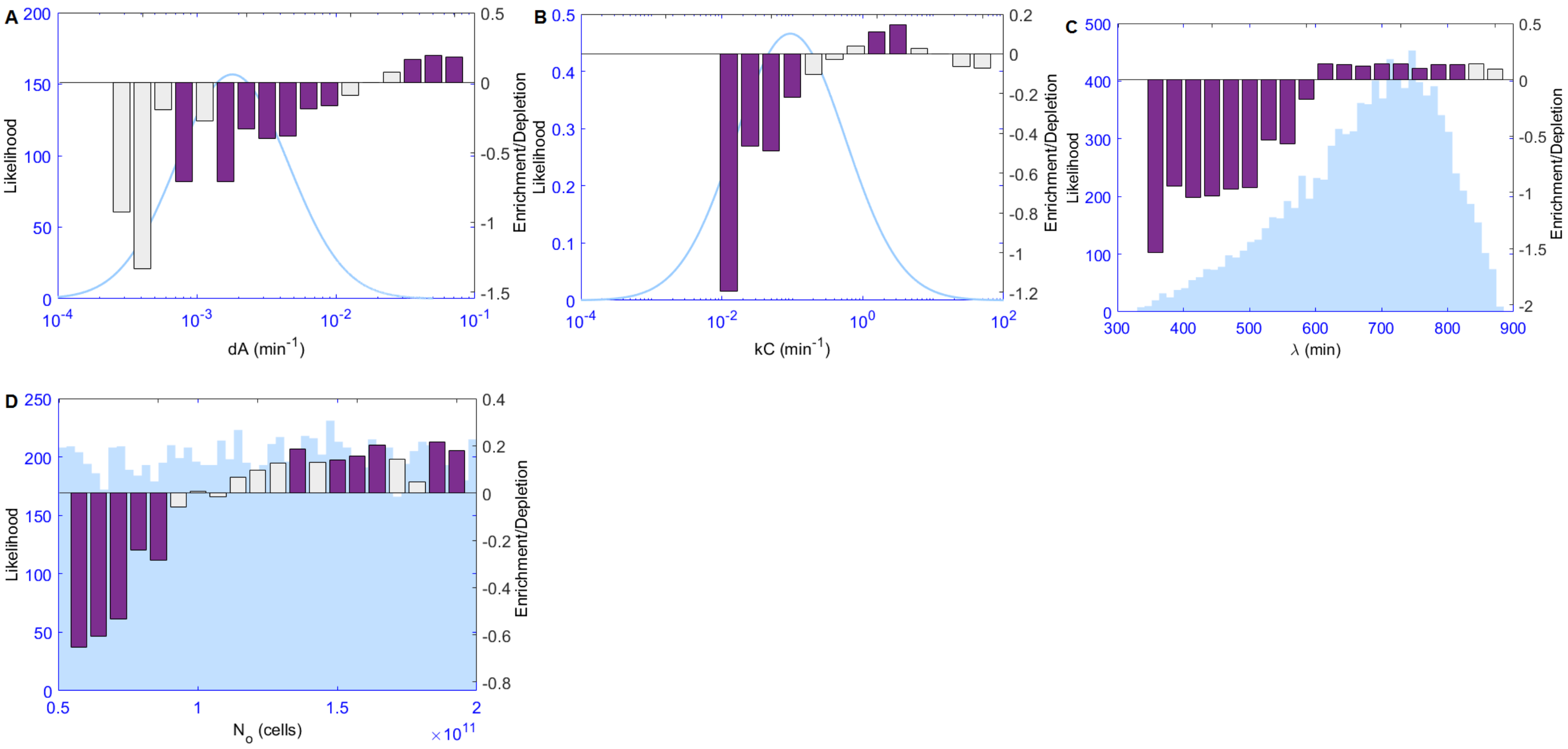

Supplementary Sp19 Fig: Comparison between the expected parameter values according to the defined priors and the actual parameters of the best models (TLL > −140) for Scenario E with *scbR* promoter being stronger than *scbA*.

Scenario E (k_{FR}<K_{FA})

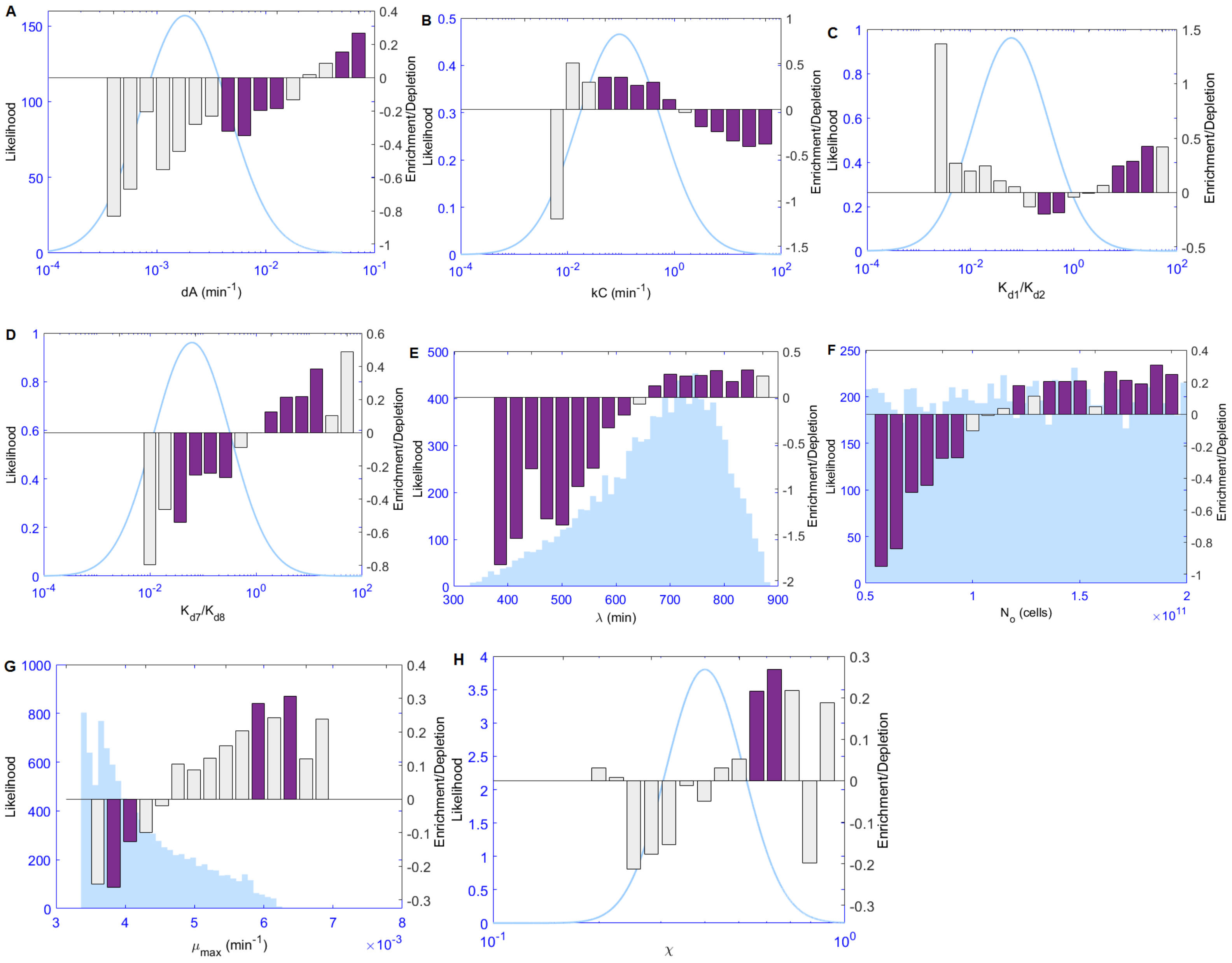

Supplementary Sp20 Fig: Comparison between the expected parameter values according to the defined priors and the actual parameters of the best models (TLL > −140) for Scenario E with *scbA* promoter being stronger than *scbR*.

Scenario F (k_{FR}=K_{FA})

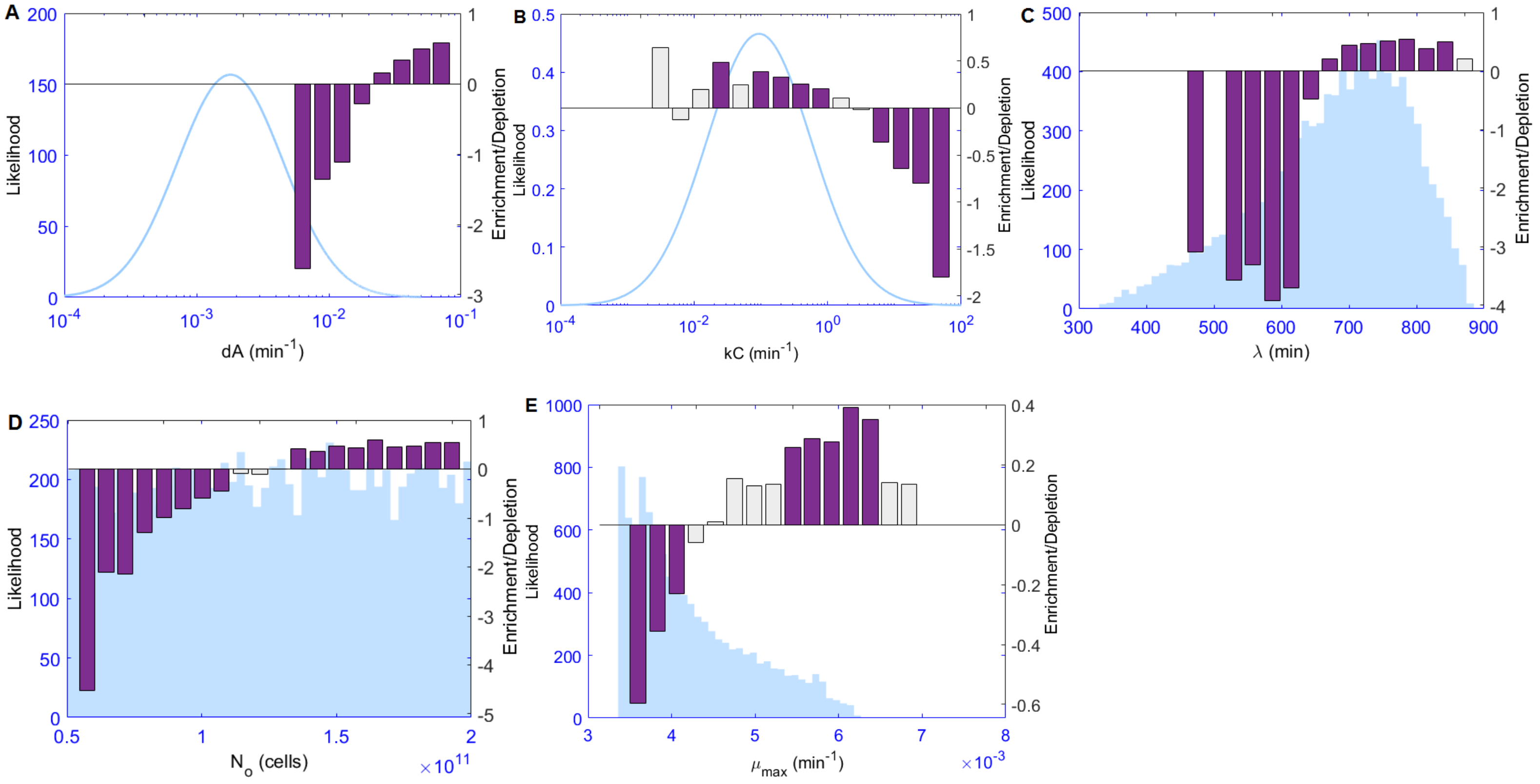

Supplementary Sp21 Fig: Comparison between the expected parameter values according to the defined priors and the actual parameters of the best models (TLL > −140) for Scenario F with promoters of equal strength.

Scenario F (k_{FR}>K_{FA})

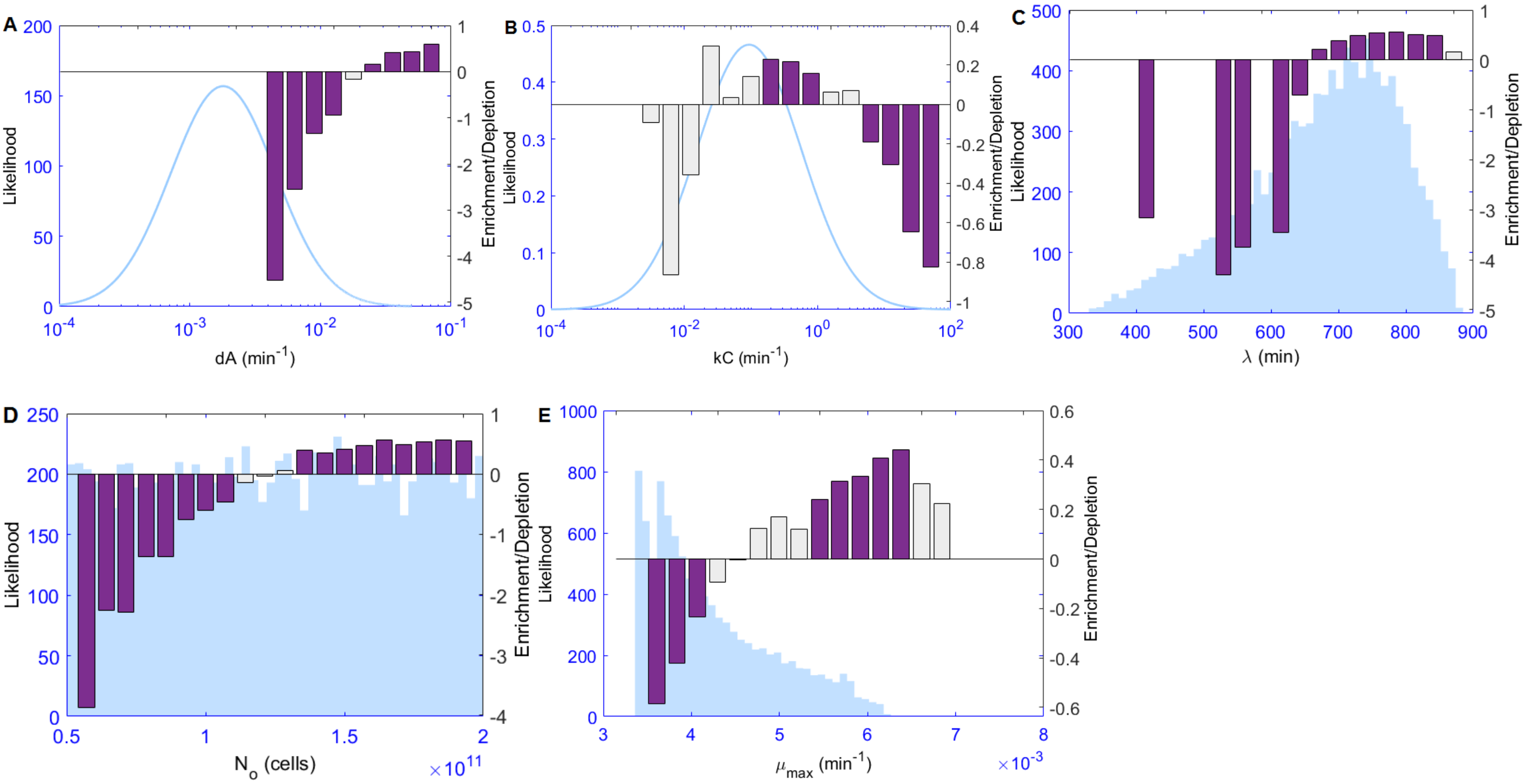

Supplementary Sp22 Fig: Comparison between the expected parameter values according to the defined priors and the actual parameters of the best models (TLL > −140) for Scenario F with *scbR* promoter being stronger than *scbA*.

Scenario F (k_{FR}<K_{FA})

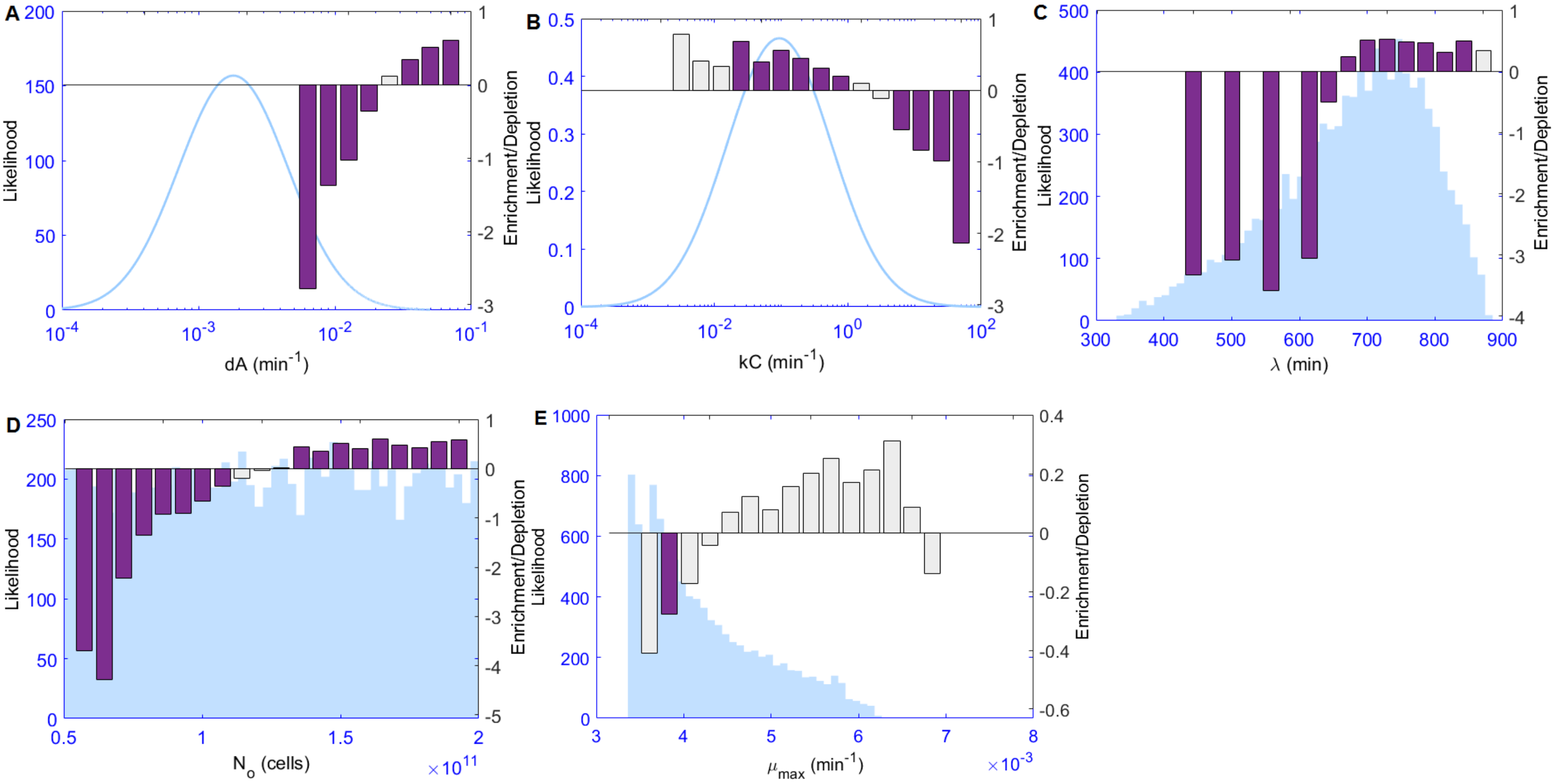

Supplementary Sp23 Fig: Comparison between the expected parameter values according to the defined priors and the actual parameters of the best models (TLL > −140) for Scenario F with *scbA* promoter being stronger than *scbR*.

Scenario G (k_{FR}=K_{FA})

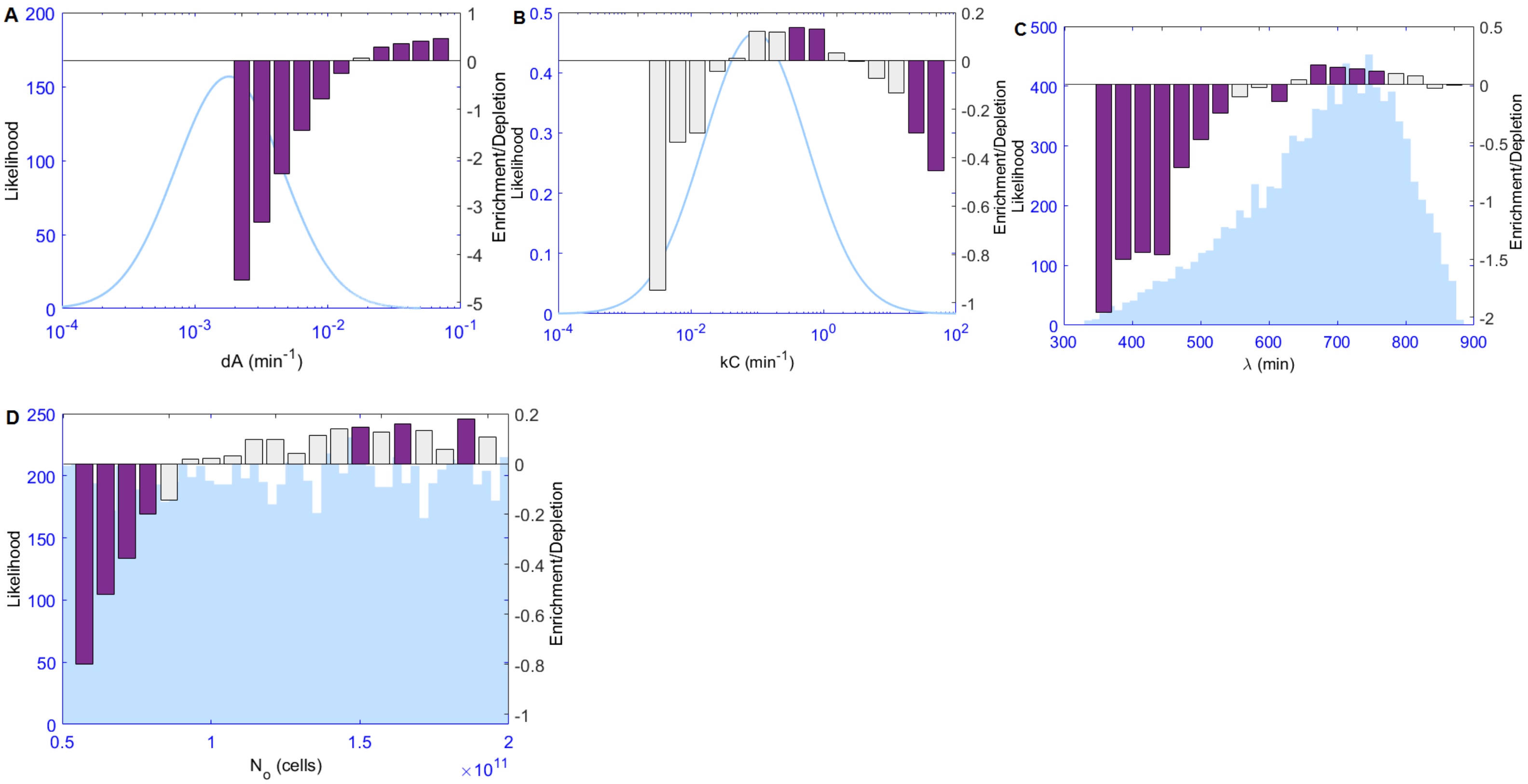

Supplementary Sp24 Fig: Comparison between the expected parameter values according to the defined priors and the actual parameters of the best models (TLL > −140) for Scenario G with promoters of equal strength.

Scenario G (k_{FR}>K_{FA})

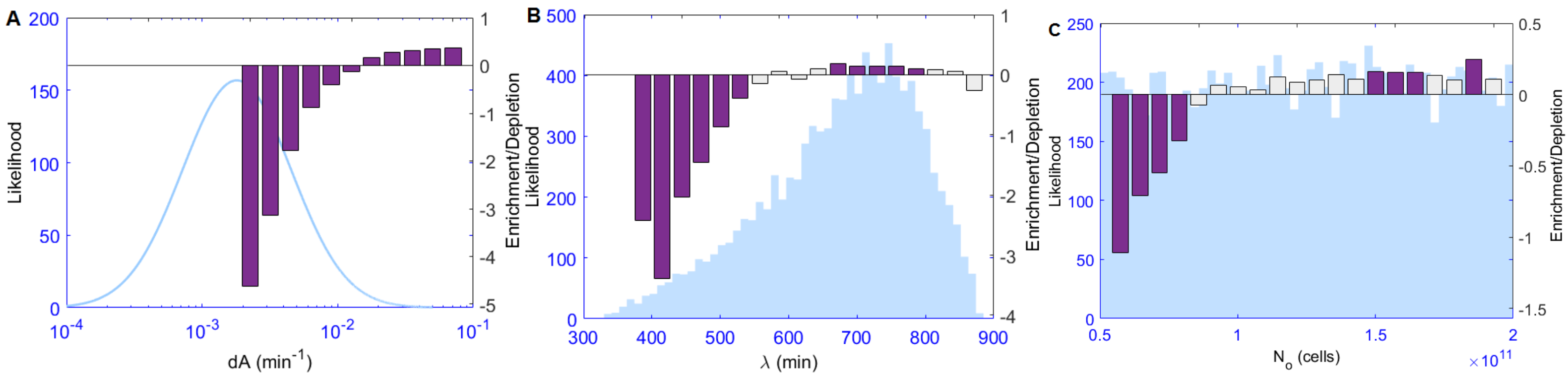

Supplementary Sp25 Fig: Comparison between the expected parameter values according to the defined priors and the actual parameters of the best models (TLL > −140) for Scenario G with *scbR* promoter being stronger than *scbA*.

Scenario G (k_{FR}<K_{FA})

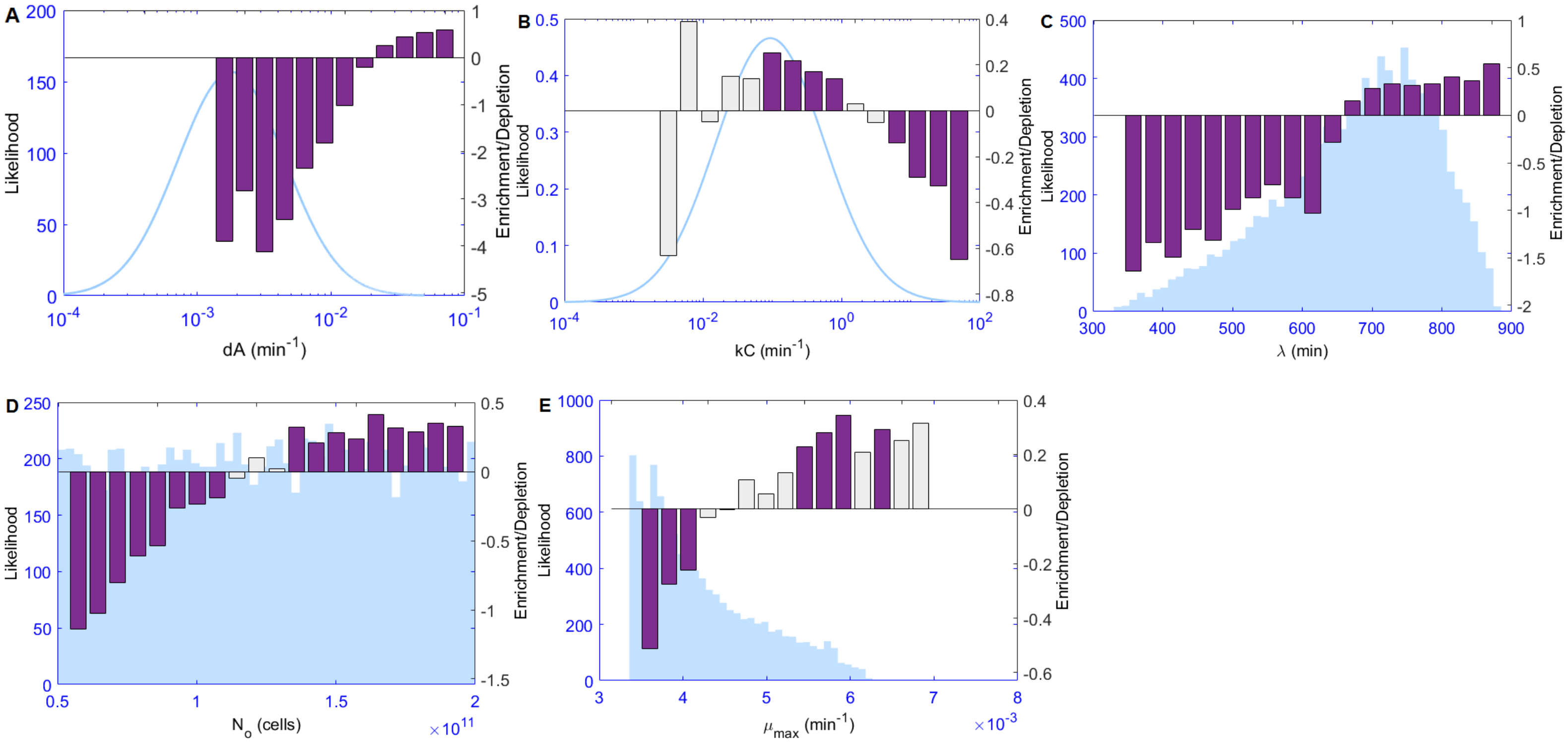

Supplementary Sp26 Fig: Comparison between the expected parameter values according to the defined priors and the actual parameters of the best models (TLL > −140) for Scenario G with *scbA* promoter being stronger than *scbR*.

Scenario H (k_{FR}=K_{FA})

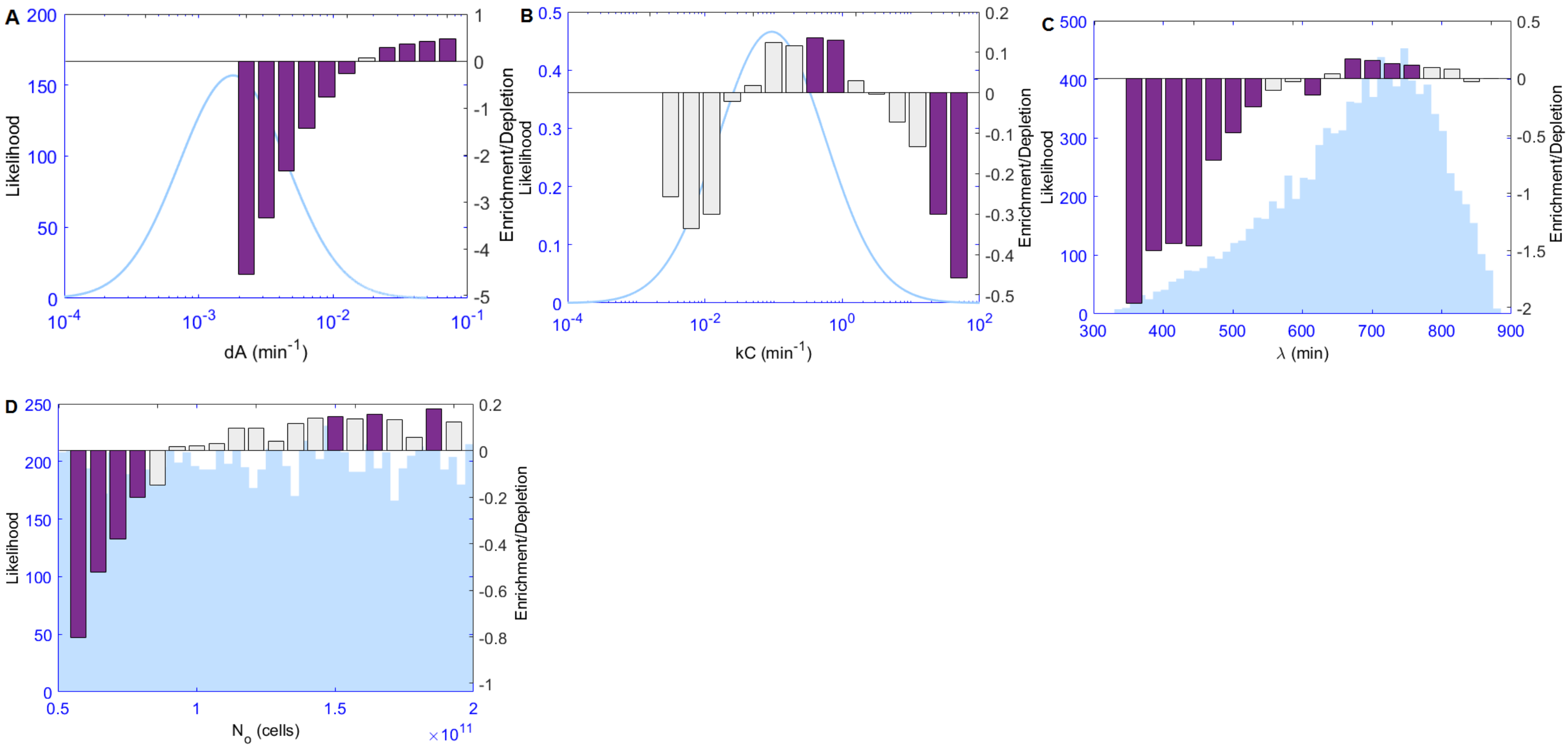

Supplementary Sp27 Fig: Comparison between the expected parameter values according to the defined priors and the actual parameters of the best models (TLL > −140) for Scenario H with promoters of equal strength.

Scenario H (k_{FR}>K_{FA})

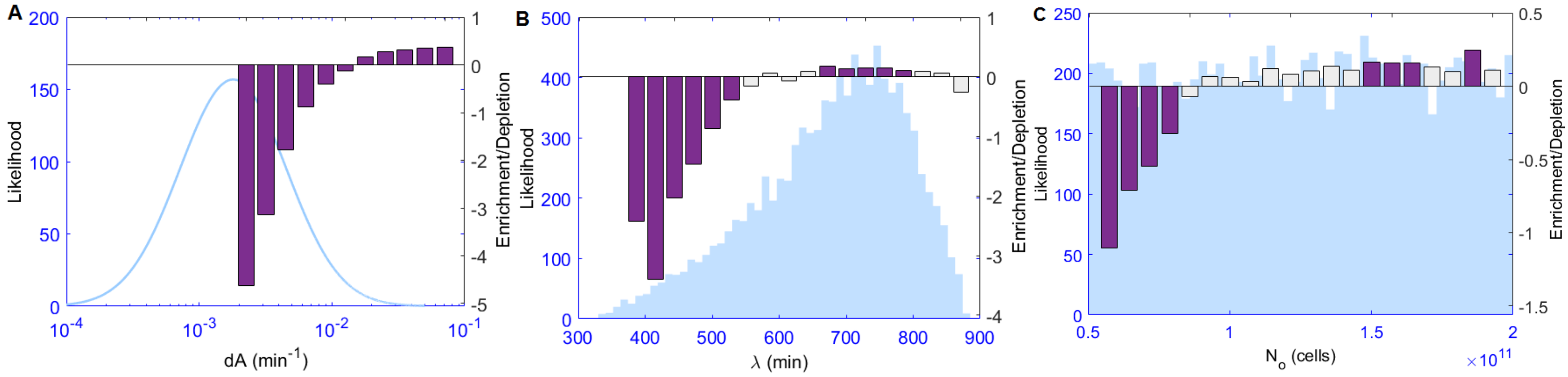

Supplementary Sp28 Fig: Comparison between the expected parameter values according to the defined priors and the actual parameters of the best models (TLL > −140) for Scenario H with *scbR* promoter being stronger than *scbA*.

Scenario H (k_{FR}<K_{FA})

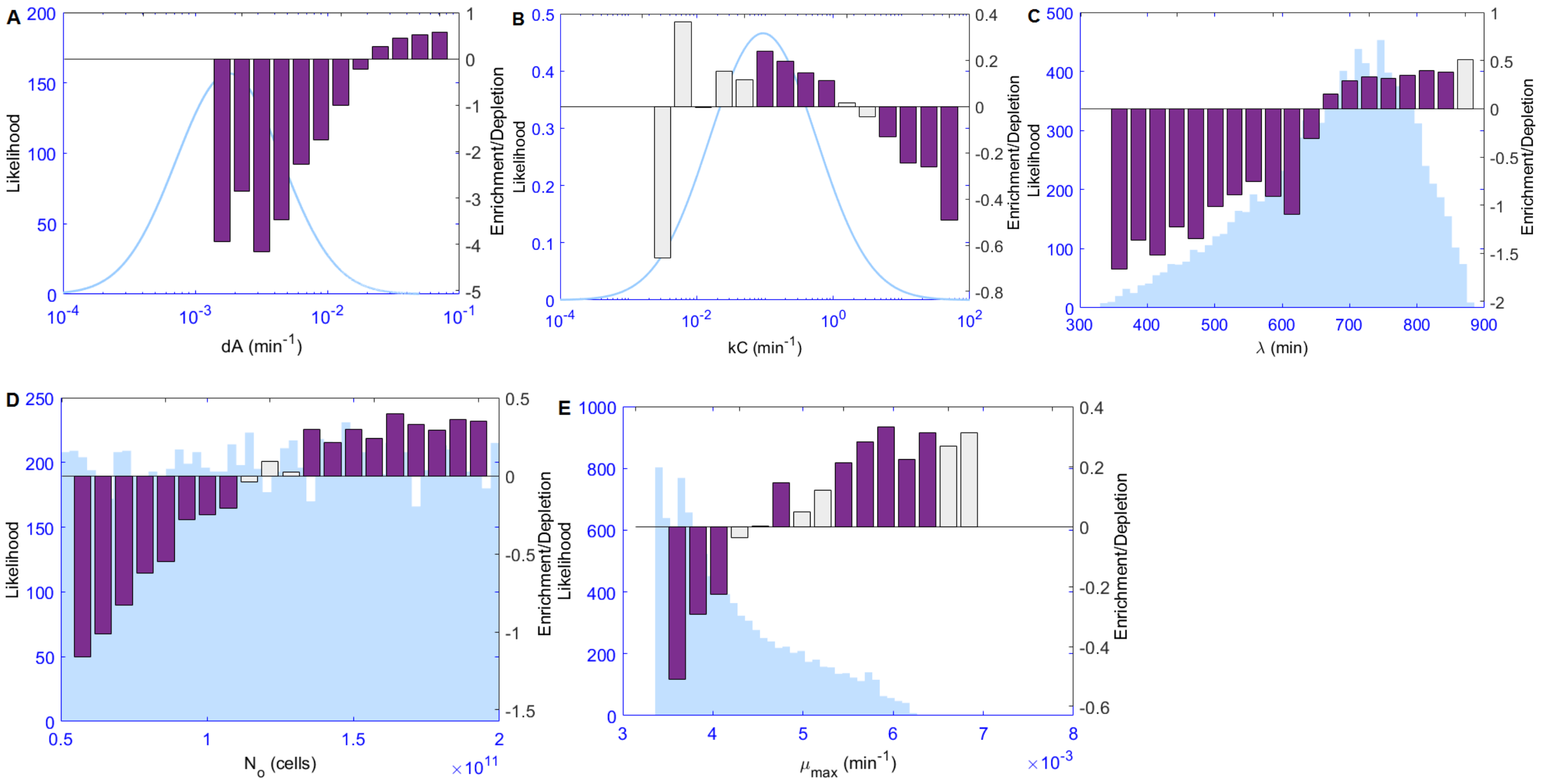

Supplementary Sp29 Fig: Comparison between the expected parameter values according to the defined priors and the actual parameters of the best models (TLL > −140) for Scenario H with *scbA* promoter being stronger than *scbR*.
