# Supplementary material for: Unravelling the γ-butyrolactone network in Streptomyces coelicolor by computational ensemble modelling
Source: PLoS Comput Biol. 2020 Jul 10;16(7):e1008039. doi: 10.1371/journal.pcbi.1008039 (PMC7384680; doi:10.1371/journal.pcbi.1008039)
Supplement: S5 Appendix — (PDF) [file pcbi.1008039.s005.pdf]

S5 Appendix – Model uncertainty in sloppy models

Like most computational models in systems biology, our models are certainly "sloppy" in the statistical sense of the word, as first highlighted by Gutenkunst *et al.* (2007) [1]; this means that even with very abundant and accurate data, it would not be possible to estimate individual parameters with sufficient accuracy to allow well-constrained predictions. The ensemble modelling approach applied here is one of the methods to address this inherent limitation. Instead of trying to fit individual parameters, we compare different models in their ability to predict systems behaviour, based on ensembles of parameter sets. This allows identifying a "collective fit", rather than trying to identify a specific fit for each individual parameter. This approach was first suggested by Gutenkunst *et al.* (2007) [1] as the appropriate general strategy to overcome the limitations imposed by the universal sloppiness of mechanistic models in molecular biology.

While we do not calculate the posterior distribution of individual parameters, the (sub)ensemble of highest-ranking models (i.e., the collectively best fitting parameter sets) for each mechanistic scenario describe how our beliefs should be updated based on the "collective fit", with associated well-founded uncertainty estimates. The procedure, based on a small amount of available experimental data, will not result in tight constraints for any of the individual parameter values, most of which will not have substantially different posterior distributions, compared to our prior knowledge. Nevertheless, as demonstrated by Gutenkunst *et al.* (2007) [1], their entirety, represented by the parameter combinations in the models with the highest log-likelihood, collectively will "enable usefully tight quantitative predictions" of systems behaviour. More importantly, they also allow distinguishing model topologies (scenarios) and their predictive ability, based on a predictive check as proposed by Box (1980) [2], considering that the parameters for all scenarios are sampled from the same distributions (and, in fact, the exact same parameter sets are used for all of the model scenarios).

The figures below illustrate and explain how our ensemble modelling approach corresponds to the suggestions of Gutenkunst *et al.* (2007) [1], based on their Fig 2 Sloppiness and Uncertainties.

References

1. Gutenkunst RN, Waterfall JJ, Casey FP, Brown KS, Myers CR, et al. Universally Sloppy Parameter Sensitivities in Systems Biology Models. PLOS Computational Biology. 2007; 3(10): e189. https://doi.org/10.1371/journal.pcbi.0030189
2. George EPB. Sampling and Bayes' Inference in Scientific Modelling and Robustness. Journal of the Royal Statistical Society Series A (General). 1980;143(4):383-430.

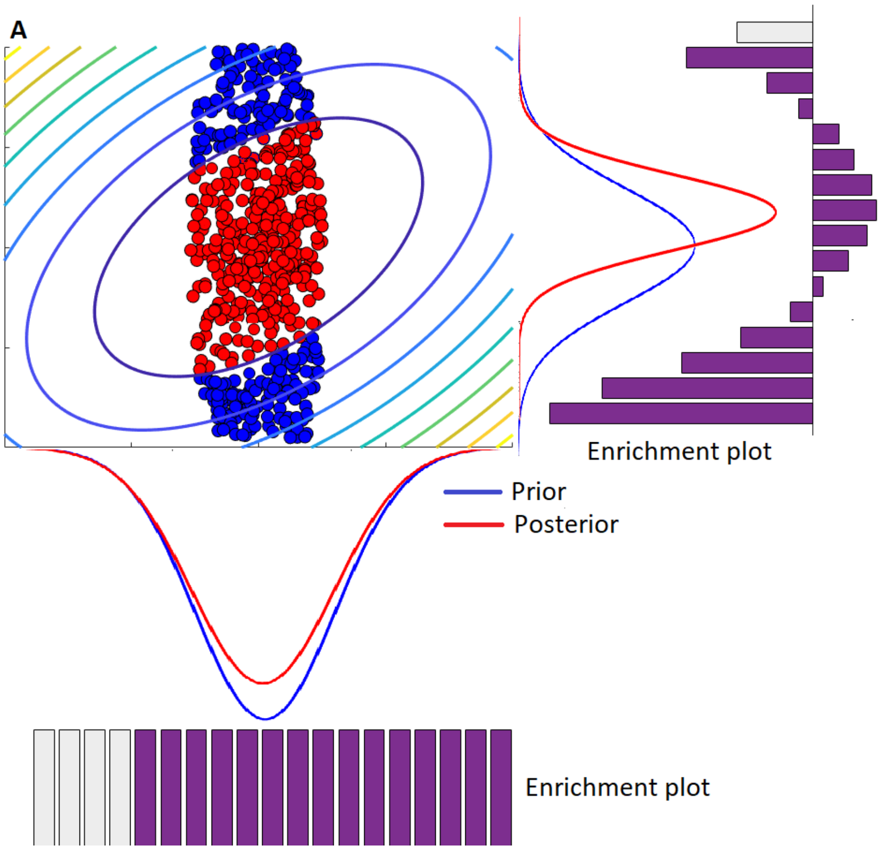

Fig A: Change in posterior distributions based on enrichment analysis. Contour lines indicate the likelihood surface of a sloppy model with two relevant parameters. The cloud of dots corresponds to our ensemble of sampled models. Red dots indicate well fitting models, blue dots poorly fitting models. One of the parameters (horizontal axis) was certain before; therefore, no further improvement of posterior is indicated by the enrichment analysis. The other parameter (vertical axis) was very uncertain; therefore the enrichment plot indicates a narrowed posterior.

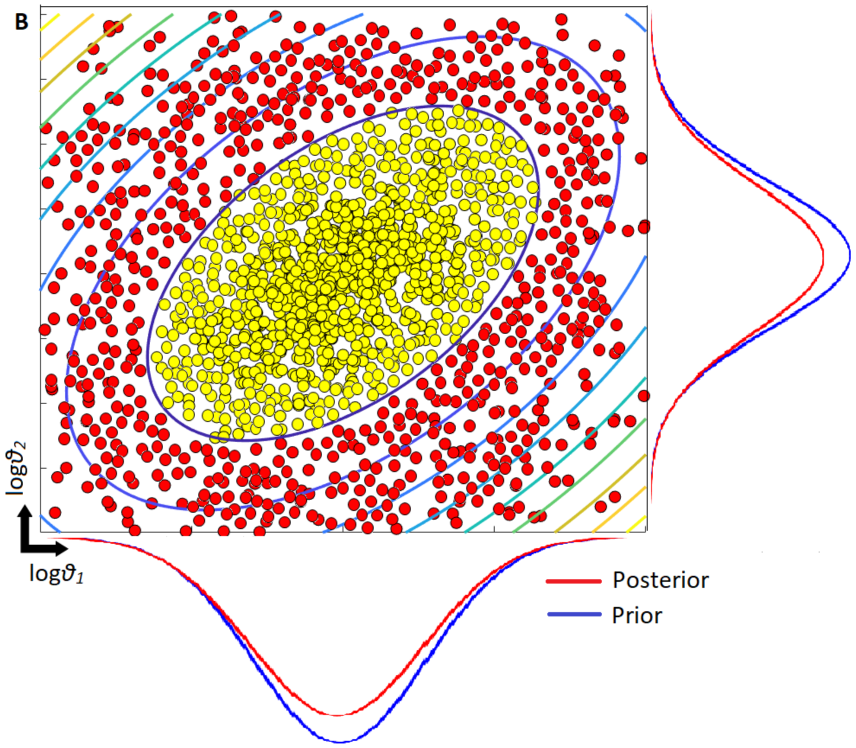

Fig B: Both parameters were (a priori) uncertain, and because the model is sloppy, their posteriors are not much more constrained than the priors. However, the ensemble modelling would still find the parameter combinations that allow good predictions (i.e, allow a collective fit, as described by Gutenkunst et al.).

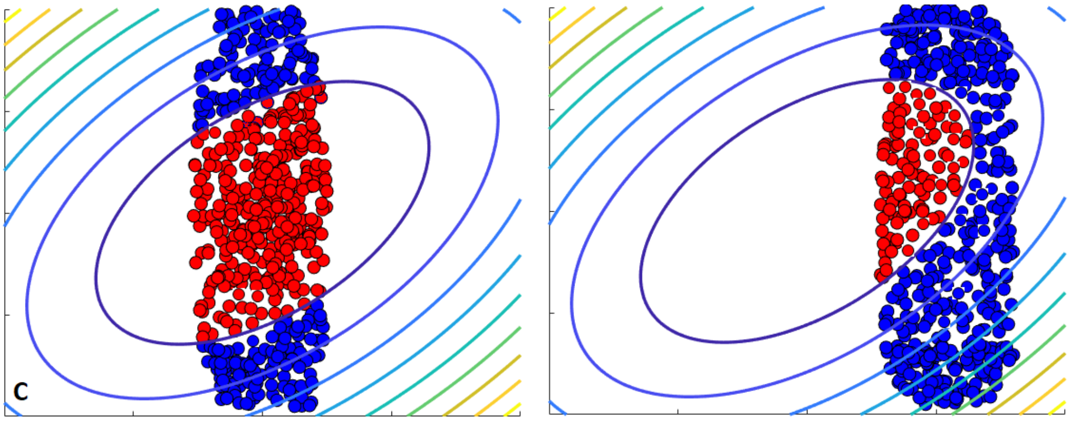

Fig C: A good ensemble model (left) differs from an implausible model (right) in the number of ensemble members that yield good predictions. This is independent of whether we calculate posteriors (which might not be very helpful, given the sloppiness of the models and the paucity of the new data). We still can compare model performance rigorously, comparing the collective fit (sensu Gutenkunst et al.), i.e., performing a prior predictive check, as introduced by Box (1980).
